# Perm-seq: Mapping Protein-DNA Interactions in Segmental Duplication and Highly Repetitive Regions of Genomes with Prior-Enhanced Read Mapping

**DOI:** 10.1371/journal.pcbi.1004491

**Published:** 2015-10-20

**Authors:** Xin Zeng, Bo Li, Rene Welch, Constanza Rojo, Ye Zheng, Colin N. Dewey, Sündüz Keleş

**Affiliations:** 1 Department of Statistics, University of Wisconsin, Madison, Wisconsin, United States of America; 2 California Institute for Quantitative Biosciences, University of California, Berkeley, California, United States of America; 3 Department of Biostatistics and Medical Informatics, University of Wisconsin, Madison, Wisconsin, United States of America; Rutgers University, UNITED STATES

## Abstract

Segmental duplications and other highly repetitive regions of genomes contribute significantly to cells’ regulatory programs. Advancements in next generation sequencing enabled genome-wide profiling of protein-DNA interactions by chromatin immunoprecipitation followed by high throughput sequencing (ChIP-seq). However, interactions in highly repetitive regions of genomes have proven difficult to map since short reads of 50–100 base pairs (bps) from these regions map to multiple locations in reference genomes. Standard analytical methods discard such multi-mapping reads and the few that can accommodate them are prone to large false positive and negative rates. We developed Perm-seq, a prior-enhanced read allocation method for ChIP-seq experiments, that can allocate multi-mapping reads in highly repetitive regions of the genomes with high accuracy. We comprehensively evaluated Perm-seq, and found that our prior-enhanced approach significantly improves multi-read allocation accuracy over approaches that do not utilize additional data types. The statistical formalism underlying our approach facilitates supervising of multi-read allocation with a variety of data sources including histone ChIP-seq. We applied Perm-seq to 64 ENCODE ChIP-seq datasets from GM12878 and K562 cells and identified many novel protein-DNA interactions in segmental duplication regions. Our analysis reveals that although the protein-DNA interactions sites are evolutionarily less conserved in repetitive regions, they share the overall sequence characteristics of the protein-DNA interactions in non-repetitive regions.

## Author Summary

Chromatin immunoprecipitation followed by high throughput sequencing (ChIP-Seq) is widely used for studying *in vivo* protein-DNA interactions genome-wide. The applicability of this method for profiling repetitive regions of the genome is limited due to short read sizes dominating ChIP-seq applications. We present Perm-seq, which implements a novel generative model for mapping short reads to repetitive regions of genomes. Perm-seq introduces a new class of read alignment algorithms that can combine data from multiple sources. We show with both computational experiments and the analysis of large volumes of ENCODE ChIP-seq data that utilizing DNase-seq derived priors in Perm-seq is especially powerful in mapping protein-DNA interactions in segmental duplication regions. This general approach enables the use of any number of histone ChIP-seq data alone or together with DNase data to supervise read allocation. Our large scale analysis reveals that although the protein-DNA interactions sites are evolutionarily less conserved in repetitive regions, they share the overall sequence characteristics of the protein-DNA interactions in non-repetitive regions.

This is a *PLOS Computational Biology* Methods paper

## Introduction

Chromatin immunoprecipitation followed by sequencing (ChIP-seq) has become a versatile high throughput assay for profiling of transcription factor (TF) binding and histone modifications. A typical ChIP-seq experiment generates millions of short reads (50–100 bps). The first step of any standard ChIP-seq data analysis pipeline involves mapping reads to a reference genome. In any given ChIP-seq experiment, a considerable fraction (5–30%) [1] of the reads can align to multiple locations on the genome (multi-reads) thereby creating ambiguity regarding their true origin. Although there have been some prior efforts in developing ChIP-seq specific mappers that can allocate multi-mapping reads to one of their mapping positions based on local counts of uniquely mapping reads [1–5] (uni-reads), the standard practice for ChIP-seq experiments is to either use only uniquely mapping reads or retain a conservative set of multi-mapping reads (e.g., with at most 2–3 mapping positions) and utilize one of the mapping positions randomly [6]. This bottleneck has serious downstream effects when characterizing regulatory elements common or specific to distinct cell types where, for example, cell-type specific characteristics that reside in repetitive regions are grossly under-represented. Similar observations have been made for MeDIP-seq analysis [7], where repetitive elements were severely underestimated in the traditional alignment and analysis of sequencing based data. This is a highly critical barrier especially to the advancement of analysis of large consortia (e.g., Encyclopaedia of DNA Elements (ENCODE)) data because significant fractions of eukaryotic genomes are composed of repetitive regions, e.g., more than half of the human genome. Genomic repeats play important roles in function and evolution of transcriptional regulatory networks [8, 9] making their functional annotation of highest biological importance. Segmental duplications played roles in creating new primate genes and contributed to human genetic variation [10]. Therefore, utilization of multi-mapping reads is especially important for characterizing regulatory activity in segmental duplications or LINE elements that harbor near-identical DNA sequences.

Current state-of-the-art approaches for allocating multi-mapping reads in both RNA-seq [11–17] and ChIP-seq [1–5] studies rely on utilizing read counts of the local neighbourhoods of the mapping positions. Therefore, these approaches have critical limitations when the local neighbourhood read counts of the mapping positions are highly similar. In order to resolve this bottleneck and improve specificity of multi-read allocation in ChIP-seq studies, we develop a novel strategy that utilizes data from DNase I hypersensitive sites sequencing (DNase-seq) experiments to inform read allocation in ChIP-seq. DNase-seq is a high-resolution assay for mapping active *cis*-regulatory elements across the genome [18, 19]. DNase-seq identifies broader regions of open chromatin which often exhibit transcription factor occupancy. Although these experiments do not provide specificity as to which regulatory factors occupy the captured accessible regions of the genome, there is a growing literature indicating their high predictive ability for identifying protein-DNA binding sites [20, 21]. Utilizing a large number of ENCODE ChIP-seq datasets from GM12878 and K562 cells, we show that DNase-seq has significant power for discriminating between the mapping locations of multi-reads with similar local ChIP-seq read counts. We develop a probabilistic model that utilizes DNase-seq derived priors (Perm-seq) and maps reads originating from highly repetitive regions with high accuracy. The Perm-seq framework is highly versatile and enables incorporation of multiple sources of data such as histone ChIP-seq for read allocation.

Our reanalysis of a large collection of ENCODE ChIP-seq datasets with Perm-seq identifies many novel protein-DNA interaction targets in highly repetitive regions of the genome, especially in segmental duplications. Our detailed analysis of these novel targets indicate that repetitive and non-repetitive modes of protein-DNA interactions share similar chromatin signatures. Although protein-DNA interaction sites in the repetitive regions are evolutionarily less conserved, they share sequence characteristics of the protein-DNA interaction regions identified by uniquely mapping reads. Furthermore, our analysis identifies that H2a.z ChIP-seq data performs as well as DNase-seq data for supervising allocation of multi-reads in highly repetitive regions.

## Results

### Discriminative power of DNase-seq for allocating multi-reads in ChIP-seq data

**Fig 1:**
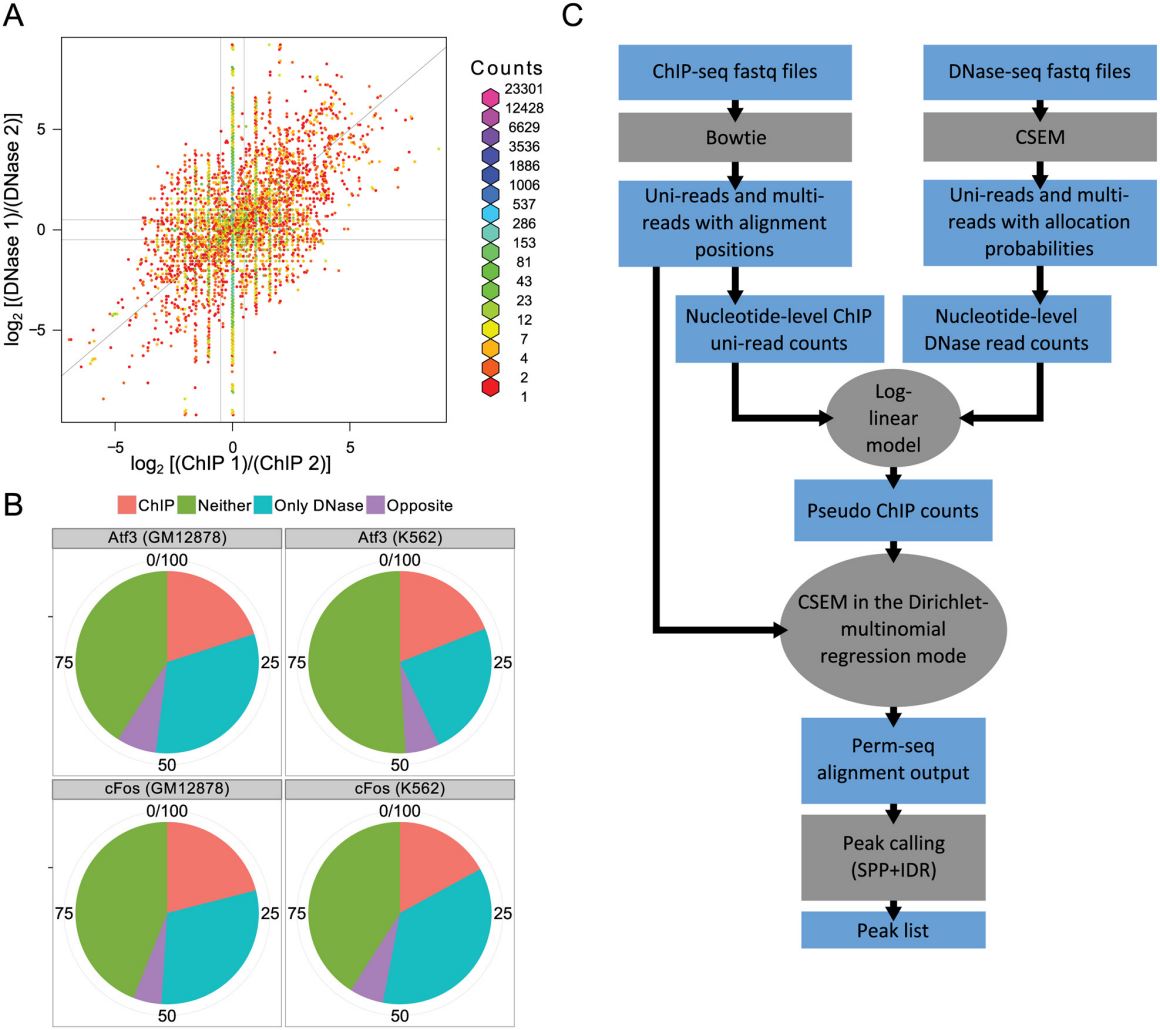
Discriminative power of DNase-seq for mapping locations of multi-reads. (a) Log base two ratios of DNase-seq versus ChIP-seq read counts in the local neighbourhoods of the two mapping locations of each multi-read in Gata2 ChIP-seq dataset (Huvec). The vertical and horizontal lines depict boundaries with the log base two ratios equal to 0.5. The proportion of read pairs in categories: ChIP: ChIP or both ChIP and DNase discriminates; Neither: Neither ChIP nor DNase discriminates; Only DNase: Only DNase discriminates; Opposite: ChIP and DNase log base 2 ratios have different signs are 23%, 44%, 26%, and 7%, respectively. (b) Classification of multi-reads with two mapping locations based on their local DNase-seq and ChIP-seq read counts into 4 groups as ChIP, Neither, Only DNase, and Opposite. (c) Overall summary of the Perm-seq pipeline.

In order to explore the discriminating power of DNase-seq data for allocating multi-mapping ChIP-seq reads, we identified all the reads that map to two different locations on chromosome 2 from an ENCODE Gata2 ChIP-seq experiment performed in Huvec cells [22]. Since all the available multi-read allocation methods utilize uni-read counts in local neighbourhoods of the mapping locations, we evaluated how different the local neighbourhoods are for the two locations that each read maps to. In addition, we also counted the number of DNase-seq reads in these neighbourhoods. This investigation revealed that the ChIP uni-read counts of the local neighbours of the two mapping locations are very similar with log base 2 fold changes smaller than 0.5, i.e., | log_2_(ChIP counts at location 1/ChIP counts at location 2)| < 0.5, for majority of the reads (70%, Fig 1(a)). However, DNase read counts together with ChIP read counts can discriminate between the two mapping locations with a log based 2 fold change of at least 0.5 for 49% of the reads, resulting in a 26% increase in the discrimination power. Similar trends in discriminative power are observed for a range of fold change thresholds (Table S1 in S1 Text).

We performed a similar analysis for four additional datasets (Fig 1(b), Fig. S1 in S1 Text): Atf3 and cFos in GM12878 and K562 cells, and observed that utilizing local ChIP read counts helps to distinguish between the two mapping locations of only 17–21% of the reads whereas DNase read counts alone were able to discriminate between two mapping locations for an additional 24–36% of the reads. For a small percentage of the reads (≤ 7%), the fold changes of DNase and ChIP read counts of the two mapping positions appear to be in opposite directions, i.e., DNase read counts in position 1 > DNase read counts in position 2, while ChIP read counts in position 1 < ChIP read counts in in position 2. This is attributable to the fact that signal measured by DNase-seq is not regulatory factor specific as well as to heterogeneity of cells the ChIP- and the DNase-seq experiments are conducted on.

### Analytical framework of the Perm-seq algorithm

Perm-seq models observed ChIP-seq read alignments conditional on DNase-seq data and significantly extends our prior work, CSEM [1] on multi-read allocation. Specifically, DNase-seq read counts are incorporated into a multinomial read generating distribution with a Dirichlet-multinomial regression model. The Dirichlet-multinomial regression model includes a log-linear prior that is a function of DNase-seq read counts. The Perm-seq framework involves three major components (Fig 1(c)). First, DNase-seq read counts are mapped to the reference genome with CSEM by taking into account multi-reads. In this step, each multi-mapping read is allocated to one of its mapping positions if the allocation probability is at least 0.9. This is quite a conservative mapping strategy for multi-reads; however it avoids generation of spurious DNase signals. Then, ChIP-seq reads are aligned to the reference genome with Bowtie [23] (using parameters -q -v 2 -a -m 99) while retaining both uni- and multi-reads. The next step involves building a log-linear model by regressing ChIP-seq uni-read counts on DNase-seq read counts. We utilize a data aggregation strategy to build such a model. Exploratory analyses of multiple ChIP-Seq datasets indicate that B-spline models provide very good fits (Fig. S2 in S1 Text) and capture how ChIP-seq read counts vary with DNase-seq signal. We use this estimated B-spline model to generate prior parameters for the Dirichlet-multinomial model and allocate multi-mapping reads to each of the mapping locations by weighting the evidence from ChIP-seq read counts of the local neighbourhoods and the corresponding DNase-seq priors. The output from Perm-seq is a file with all the uni- and multi-reads and includes final allocation probabilities for each of the mapping locations of the multi-reads. Perm-seq also provides functionality to convert this file into a BED file where each multi-read is allocated to its best mapping position with the largest allocation probability. Such a BED file can then be used by many standard peak calling methods. We used ENCODE’s uniform ChIP-seq processing pipeline [24], which is built on the peak caller SPP [25] and irreproducible discovery rate (IDR) [26] for determining the optimal number of peaks, in the analyses presented in this paper.

### Perm-seq accurately discriminates between multiple mapping locations of multi-reads

**Fig 2:**
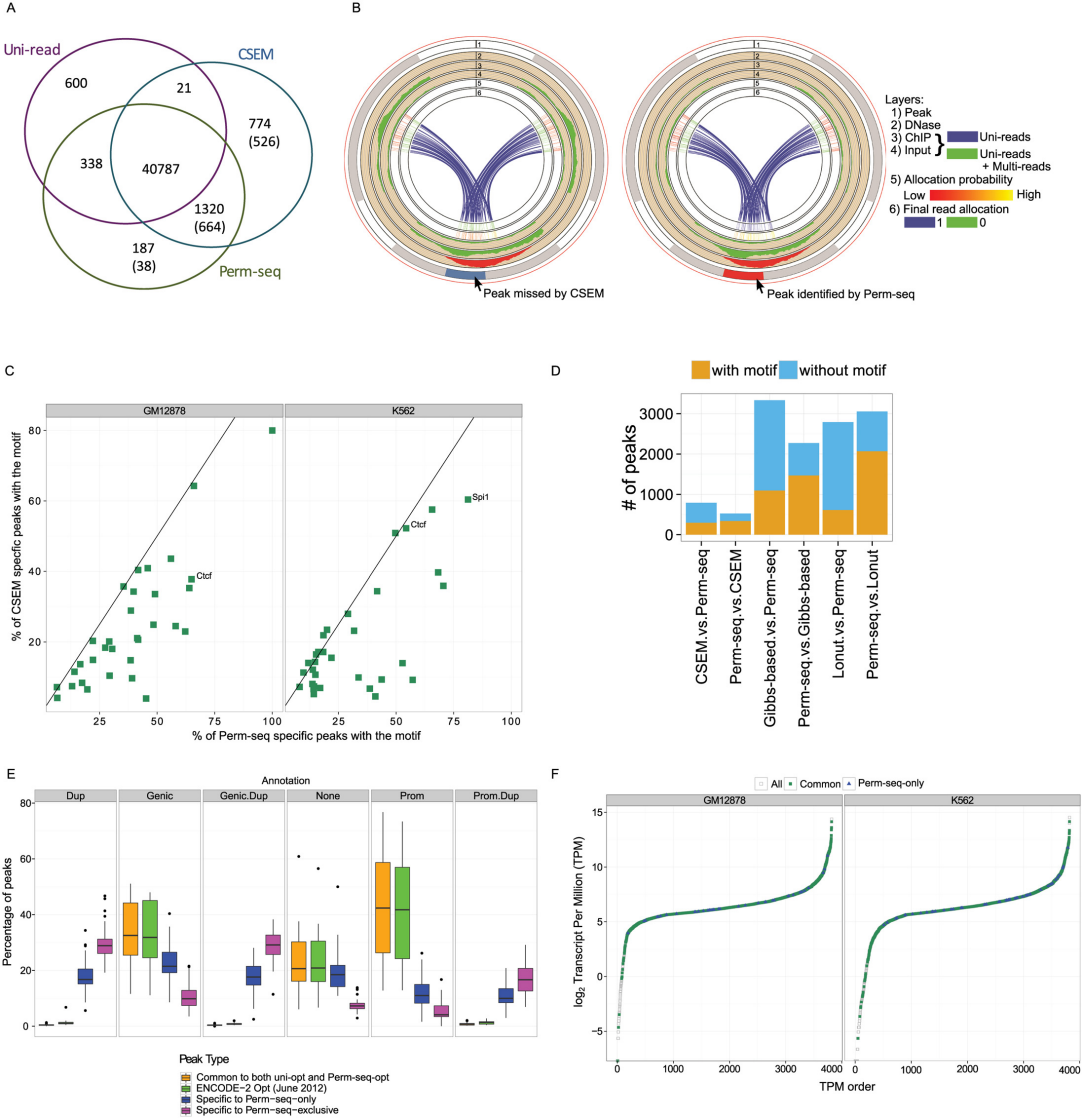
Comparison of uni-read, CSEM, and Perm-seq analysis. (a) Comparison of Ctcf optimal peak lists from uni-read, CSEM, and Perm-seq analyses. Numbers in parentheses denote comparisons of the optimal peak lists with the relaxed peak lists. For example, there are 1320 peaks identified by the Perm-seq and CSEM analyses and missed by the uni-read analyses. 664 of these peaks are still missed by the uni-read analysis even if we consider comparison of the Perm-seq and CSEM optimal peak lists with the uni-read relaxed lists. (b) Circos plots of CSEM (left) and Perm-seq (right) read allocation for reads mapping to four segmental duplication regions with coordinates chr1:143,880,003–143,978,943, chr1:206,072,707–206,171,611, and chr1:143,880,003–144,005,301, chr1:120,872,119–249,250,621. (c) Percentages of Perm-seq specific and CSEM specific peaks with the most significant motifs identified from the *de novo* sequence analysis of the intersection peaks, i.e., peaks common to uni-read, CSEM, and Perm-seq analysis. (d) Comparison of the Ctcf peak sets from GM12878 between Perm-seq, CSEM, Gibbs-based [2], and Lonut [4]. x.vs.Perm-seq denotes optimal peaks of method “x” not identified by Perm-seq. Similarly, Perm-seq.vs.x denotes optimal peaks of Perm-seq not identified by method “x”. (e) Annotation of the K562 peaks with respect to segmental duplications. Categories are: Prom.Dup: peaks that are in promoter regions (± 2500 bps of TSS) of RefSeq genes that reside in segmental duplications; Prom: Peaks in promoter regions (excludes peaks in Prom.Dup); Genic.Dup: peaks that are within [-10000 bps of TSS, +1000 bps of TES] of RefSeq genes that are in segmental duplications (excludes peaks in Prom.Dup); Genic: peaks that are within [-10000 bps of TSS, +1000 bps of TES] of RefSeq genes (excludes peaks in Genic.Dup, Prom.Dup); Dup: peaks that are in segmental duplications (excludes Prom.Dup and Genic.Dup); None: peaks that do not fall into any of the other defined categories. (f) Genes are ordered with respect to RNA-seq transcripts per million (TPM) values. Genes with a Common Pol2 peak in their promoters are depicted with green whereas genes with only Perm-seq-only peaks are depicted in blue.

The overall effect of using prior information in ChIP-seq multi-read allocation with Perm-seq is an increase in the number of detected peaks compared to analyses that utilize only uni-reads (uni-read analysis) (Fig. S3 and Tables S2 and S3 in S1 Text) and a decrease in the number of peaks compared to analyses using multi-reads without prior information (CSEM). Analysis of 64 ChIP-seq datasets from GM12878 and K562 cells resulted in an average increase of 8.0% (with a standard deviation of 13.1%) in the number of peaks when comparing uni-read analysis with Perm-seq and a decrease of 4.6% (with standard deviation 9.8%) when comparing Perm-seq with CSEM. Fig 2(a) summarizes the numbers of peaks and peaks overlapping across the three different peak classes, namely, uni-read, CSEM, and Perm-seq in both the optimal and the relaxed mode of peak calling for transcription factor Ctcf. Optimal peak sets are obtained at an IDR of 2%. The relaxed peak sets are super sets of the optimal peaks and include both high signal peaks and regions that do not show any ChIP enrichment. We included comparisons with the relaxed peak sets to ensure that our results hold irrespective of the specific IDR threshold used for peak calling. We observed that 1320 peaks are identified by both Perm-seq and CSEM analyses, whereas Perm-seq identified 187 peaks that are not part of CSEM optimal list, and similarly, CSEM identified 774 peaks that are not part of Perm-seq optimal peak list. We evaluated peaks specific to CSEM and Perm-seq to assess whether utilizing the prior information is eliminating false positive and false negative peaks. Fig. S4, Fig. S5, and Fig. S6 in S1 Text display examples of each type of peak. We also provide circos plots of read allocation by Perm-seq and CSEM for a Perm-seq peak with reads distributed over four segmental duplication regions to elucidate how DNase information is guiding read allocation (Fig 2(b)). The three regions depicted in the circos plots span partially overlapping segmental duplications chr1:143,880,003–143,978,943, chr1:206,072,707–206,171,611, and chr1:143,880,003–144,005,301, chr1:120,872,119–249,250,621. Multi-read allocation without prior information distributes the set of multi-reads over these three regions because of the similarities in their local uni-read ChIP counts and fails to identify a peak in any of them. However, as depicted with the DNase-seq track, only one of these three regions has considerable DNase-seq signal indicating regulatory activity. Utilizing DNase-seq prior allocates most of the multi-reads to the region with high DNase-signal and successfully identifies a peak with a canonical Ctcf motif. Segmental duplication regions are defined as regions in which at least 1000 bps of the total sequence (containing at least 500 bps of non-repeat masked sequence) align with a sequence identity of at least 90% (percentage of matching bases out of the aligning bases) [27]. However, high sequence identity does not necessarily imply large fraction of non-unique, i.e., not uniquely mappable, sub-sequences (e.g., 50mers) within the aligning region. Even though ChIP-reads can map to non-unique regions within the segmental duplication, DNase reads can cover more of the uniquely mappable regions and thereby discriminate between two high sequence identity regions. Fig. S7 in S1 Text displays the maximum and the median of the run lengths of adjacent non-unique 50mers across all the segmental duplications as a function of the alignment sequence identity. Overall, only 14.7% of the segmental duplications have a maximum run length larger than 300 bps indicating that for a majority of the segmental duplication regions, there are many mappable positions within and in the immediate vicinity of the 300 bps window a ChIP-seq peak might cover. DNase reads aligning to such bases boost the signal for the ChIP-seq peak and discriminate between highly similar regions.

We further compared the peak sets from Perm-seq and CSEM with sequence analysis. We learnt the most enriched motif in the top 500 peaks of the intersection of the three peak sets (peaks from the uni-read, CSEM, and Perm-seq analyses) using the MEME suite [28] and evaluated the occurrence of this motif in the Perm-seq specific and the CSEM specific peak sets. Fig 2(c) displays motif occurrences in these two types of peak sets across 32 factors in GM12878 and K562 cells, respectively (Table S4 in S1 Text). On average, 13% more of the Perm-seq specific peaks have a motif occurrence compared to CSEM specific peak sets. Furthermore, 13 of the 64 Perm-seq specific peak sets exhibit at least 25% more motif occurrence compared to CSEM specific peak sets, indicating that much larger fractions of the Perm-seq only peak sets are supported by the sequence analysis. Overall, the differences in motif occurrences of the two methods in both cell types are significant with Wilcoxon-rank-sum test (p-values < 0.001). Fig 2(d) extends this comparison on the Ctcf ChIP-seq data from GM12878 to include a Gibbs-based approach [2], which is another multi-read allocation method similar to CSEM, and Lonut [4], which is the most recently published multi-read allocation method. Overall, we observe that Perm-seq reduces numbers of peaks without motif and increases the numbers of peaks with the motif.

#### Data-driven computational experiments evaluating the improvement in multi-read allocation due to Perm-seq

In addition to evaluating the performance of Perm-seq on a large collection of ENCODE datasets, we also performed data-driven simulations to asses the improvement due to utilization of DNase-seq prior (Section 5 of S1 Text). These simulations used the actual DNase-seq data and generated reads using the read density estimated from the Atf3 ChIP-seq sample in GM12878 cells. Although the simulation model did not induce any model misspecification for Perm-seq, the resulting simulated data captured the mean ChIP versus DNase read count relationship observed in actual data well (data not shown). We first compared Perm-seq to CSEM, Gibbs-based [2], and a random allocation approach that randomly allocates multi-reads to one of their mapping locations. These choices were driven by the ease of the use of the software in the simulations, computational times, and the ability to provide multi-read allocation without relying on peak calling. The latter criterion is especially critical for flexibility in performing downstream peak calling. We simulated read data based on the estimated parameters from the Atf3 ChIP-seq sample from GM12878 cells and evaluated multi-read allocation accuracy for low, medium, and high sequencing depth settings. Since both the random allocation and Gibbs-based approaches assign multi-reads to a single position, we assigned multi-reads allocated by Perm-seq and CSEM to their mapping locations with the maximum allocation probabilities. In the case of two or more positions with the same largest allocation probability, i.e., ambiguous reads, we randomly assigned reads to one of these positions. Fig. S8(a) in S1 Text displays the proportion of correctly allocated reads at different sequencing depths and indicates that Perm-seq performs better than other three approaches at all depths. On average, Perm-seq results in an average increase of 7.7% to 15.4% compared to CSEM and 9.5% and 34.5% compared to the Gibbs-based approach. These improvements are overall much larger than the typical differences between commonly used aligners with good performances, e.g., bowtie vs. bwa [29]. Furthermore, our downstream peak level analysis indicate that these differences in multi-read allocation accuracies translate to higher sensitivity (4.6%-10% higher than CSEM and 3.4%-40.1% higher than the Gibbs-based with all standard errors less than 2% for the three settings) and better positive predictive value (3.7%-5.3% higher than CSEM with all standard errors less than 2% and 4.8%-17.6% higher than Gibbs-based with standard errors up to 2.8% for the three settings) for Perm-seq compared to CSEM and the Gibbs-based approach (Fig. S8(b) in S1 Text).

We next performed a more detailed comparison between Perm-seq and CSEM to understand the added utility of using DNase-seq prior in a more controlled setting where the only difference between Perm-seq and the alternative method is the use of prior. We further stratified the results to evaluate the performances by taking into account the read ambiguity. Overall, we observe up to 20.7% (with a standard error of 0.61%) improvement by Perm-seq compared to CSEM in read allocation accuracy (Fig. S9 in S1 Text). Although the overall discrepancy between the performances of the two approaches decreases with increasing sequencing depth, Perm-seq allocates significantly more reads accurately for the cases where DNase-seq data provides discriminating power (Fig. S10(a–c) in S1 Text). These simulations also support that the reads with Perm-seq allocation probability of at least 0.5 are more accurate and have fewer false positives and hence should lead to better downstream analysis for peak calling.

As a final computational experiment, we compared performances of different approaches based on a read trimming experiment. Specifically, we utilized a GM12878 Ctcf experiment with 101bps paired-end (PE) reads [30] (GEO accession number: GSM1233887) and set uni-read alignments of the original reads (PE101) as the true origin of the aligning reads. Then, we trimmed the reads and sampled one end of each read pair to generate *in silico* single-end datasets with read lengths 36bps, 50bps, 75bps, and 101bps. As we trimmed the reads, a fraction of PE101 uni-reads became multi-reads. Supplementary Table S5 in S1 Text reports the proportion of correctly aligned multi-reads by different methods for each read design. Overall, we observe that Perm-seq and CSEM perform similarly and they outperform both the random allocation approach and the other multi-read mapping methods. In this particular experiment, we did not observe any gain from using prior data. Further investigation of the Perm-seq and CSEM alignment results indicated that majority of the the multi-reads that were wrongly allocated did not have discriminating DNase-seq data at their mapping locations. Although this is an instance where we are not seeing any gain from using prior data, it illustrates that Perm-seq does not lead to additional false positive alignments when DNase-seq is not providing discriminating power, i.e., impact of weak prior.

### Perm-seq complements ENCODE-derived peak sets by identifying protein-DNA interaction sites enriched in segmental duplications

After studying the accuracy of Perm-seq for multi-read allocation, we sought to understand the broader impacts of incorporating multi-mapping reads in ChIP-seq analysis. In what follows, the comparisons are focused on (i) peak sets identifiable by both the uni-read and prior-enhanced multi-read analysis with Perm-seq (Common) and are obtained by intersecting optimal peak lists from uni-read and Perm-seq analyses; (ii) peak sets identifiable only with the prior-enhanced multi-read analysis with Perm-seq (Perm-seq-only), i.e., Perm-seq optimal peaks not overlapping the uni-read optimal peaks; and finally, (iii) the subset of the Perm-seq-only peaks that are not overlapping the uni-read relaxed peak sets (Perm-seq-exclusive). Overall, our Common set is highly comparable to the peak sets from the ENCODE project which utilized Bwa [6] instead of Bowtie [23] for aligning the reads.

We first annotated these peak sets in terms of segmental duplication regions (Fig 2(e) and Fig. S11 in S1 Text) and observed that both Perm-seq-only and Perm-seq-exclusive peak sets identify significantly more peaks in segmental duplication regions compared to the Common peak sets. For example, more than 60% (90%) of the Perm-seq-only (Perm-seq-exclusive) Ctcf peaks reside in segmental duplications in contrast to only 2% of the Common Ctcf peaks in segmental duplications. The largest percentage of Common peaks located in segmental duplications is only 3% across all the TFs. In contrast, for all the TFs except for Usf2, Jund, and Max, more than 30% of the Perm-seq-only peaks reside in segmental duplications. Similarly, at least 50% of all but Nfe2 Perm-seq-exclusive peaks overlap with segmental duplications. These results emphasize that characterizing regulatory activity in segmental duplications requires utilization of multi-reads.

Notably, Perm-seq-exclusive peak sets contain many peaks that reside in promoter regions of genes in segmental duplication regions. Our Common peak set has very similar annotation characteristics to the ENCODE peak sets [24] and that Perm-seq-exclusive peak sets enhance the ENCODE peak sets by the addition of many peaks in segmental duplication regions.

In Fig. S12 and Fig. S13 in S1 Text, we compared enrichment of Common and Perm-seq-exclusive peaks for other classes of repetitive elements including short and long interspersed elements (SINE and LINE retrotransposons), DNA transposons, and long terminal repeat elements (LTRs). In contrast to enrichment for segmental duplications, enrichments of peaks for these other repetitive element classes were comparable between the Common and Perm-seq-exclusive peaks for a majority of the TFs.

Next, we explored Pol2 peaks further to assess whether Perm-seq-only peaks contributed to interpretation of RNA-seq results in GM12878 and K562 cells. Fig 2(f) displays transcripts per million (TPM) on the log base 2 scale versus the rank of 3818 genes with TPM greater than 50 either in GM12878 (2957 genes) or K562 (2921 genes) cells. Genes with a Common peak in their promoters (defined as 5000 bps downstream and 500 bps upstream of the transcription start site) are depicted in green, where as genes with a Perm-seq-only peak are depicted in blue. A total of 38 and 58 genes with low mappable promoters had Pol2 peaks only identifiable with the Perm-seq analysis in the GM12878 and K562 cells, respectively. Notably, CCL4, a chemotactic cytokine, is one of such genes which is specifically expressed in GM12878 (TPM values for K562 and GM12878 are 0 and 707.27, respectively, Fig. S14 in S1 Text). It has been recently shown that CCL4 was induced in EBV-infected B cells and was expressed at high levels in all EBV-immortalized lymphoblastoid cell lines [31] (i.e., GM12787).

### Genes associated with Perm-seq-only peaks exhibit similar expression levels to those of Common peaks

**Fig 3:**
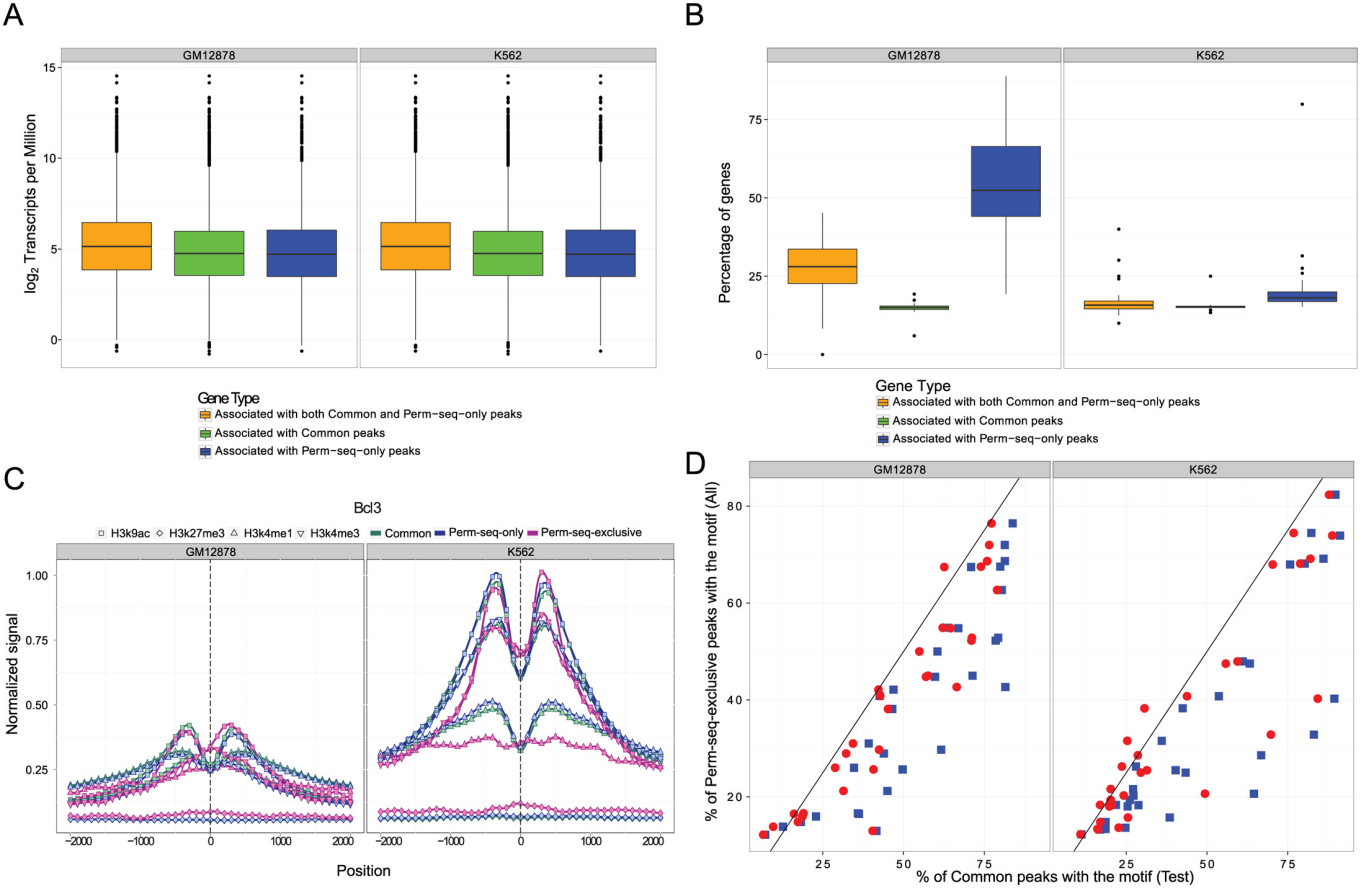
Comparison of uni-read and Perm-seq analysis. (a) Distribution of expression levels of genes with (i) Common peaks; (ii) Common and Perm-seq-only peaks; (iii) Perm-seq-only peaks. (b) Percentage of genes in segmental duplications with (i) Common peaks; (ii) Common and Perm-seq-only peaks; (iii) Perm-seq-only peaks. (c) Histone modification profiles within ± 2000 bps of the summits of the Common, Perm-seq-only, and Perm-seq-exclusive Bcl3 peaks in GM12878 and K562 cells, respectively. (d) Percentages of Perm-seq-exclusive and Common peak sets with the M1 motifs from the *de novo* sequence analysis of the top 500 Common peaks. Common peak set percentages are replaced by the percentages of occurrences in the subset of the Common peak sets (red circles) that is matched to Perm-seq-exclusive peaks in terms of ChIP signal.

We considered a subset of the peaks across all TFs that resided in the vicinity of genes (within -10000 bps upstream of the transcription start site and +10000 bps downstream of the transcription end site) and classified these genes into three classes as having (i) Common peaks, (ii) Common and Perm-seq-only peaks, (iii) Perm-seq-only peaks. The mean numbers of genes in each of the three categories were 4226.5, 289.5, and 217.4 for GM12878 and 4589.4, 403.9, and 190.5 for K562. We then compared RNA-seq data for these three classes of genes (Fig 3(a)) and observed that they have comparable expression levels, indicating that genes with only Perm-seq-only peaks are transcriptionally similar to genes that harbor Common peaks. We further evaluated the percentage of genes residing in segmental duplications within each class (Fig 3(b)) and observed that a significantly larger percentage of the genes with a Perm-seq-only peak resided in segmental duplications.

### Repetitive and non-repetitive modes of protein-DNA interactions share similar chromatin signatures

Fig 3(c) depicts (and S2 Text & S3 Text) average histone modification profiles (H3k9ac, H3k27me3, H3k4me1, H3k4me3) for the [-2000 bps, +2000 bps] window anchored at the peak summit for the three groups of peaks. We observe that Peak-seq-only peaks have almost identical profiles to those of the Common peaks. Peak-seq-exclusive peaks, in general, show lower signal for all the modifications consistent with the fact that ChIP-seq signal for these peaks are also lower; however, their overall average modification profiles match closely to those of the Common and Perm-seq-only peaks.

### Repetitive and non-repetitive modes of protein-DNA interactions share similar canonical motifs that are evolutionarily less conserved in the repetitive mode

We next sought to understand whether protein-DNA interactions followed similar sequence-specific features in non-repetitive (Common peak sets) and repetitive (Perm-seq-exclusive peak sets) modes of binding. We performed *de novo* motif analysis of both sets of peaks using the MEME suite [28] (Fig. S15 in S1 Text). *De novo* analyses on the Perm-seq-exclusive peak sets identified motifs that matched the most significant motifs (denoted as M1 in the MEME analysis) from the *de novo* motif analyses of the Common peaks for 78.1% and 71% of the factors in GM12878 and K562 cells, respectively. The factors for which M1 motifs of the Common peaks were not identified *de novo* in the Perm-seq-exclusive sets are Bclaf1, Chd2, Ets1, Nr2c2, Sin3a, Taf1, and Tbp for GM12878 and Bcl3, Bclaf1, Chd2, Ets1, Nr2c2, Pol3rg, Sin3a, Taf1, and Tbp for K562. The majority of these factors are either chromatin modifiers (Chd2, Sin3a), general Pol2 associated factors (Taf1, Tbp), or the sizes of their Perm-seq-exclusive peak sets are smaller than 500 (Nr2c2, Polr3g, Ets1 (GM12878), Bclaf1 (K562), Bcl3). For 65.6% (GM12878) and 53.1% (K562) of the factors, the most significant motifs (M1s) from the Common peak sets were identified as the M1 motif in the Perm-seq-exclusive analysis.

We next compared the occurrences of the M1 motifs of the non-repetitive mode of each factor in the peak sets. We used all the Perm-seq-exclusive peaks and the subset of the Common peaks that were not utilized in the *de novo* motif analysis, i.e., test peaks from Fig. S15 in S1 Text. Similar results were obtained when Common peaks used in the *de novo* motif analysis were included (data not shown). Overall, we observed that Perm-seq-exclusive peaks tend to have lower enrichment for the M1 motif (Fig 3(d)). To evaluate whether this was a consequence of the overall lower ChIP signal of the Perm-seq-exclusive peaks compared to Common peaks, we considered the subset of Common peaks that matched the signal strength of the Perm-seq-exclusive peaks. Fig 3(d) indicates that when the signal strength is taken into account, the enrichment of the M1 motif tends to become more comparable in the non-repetitive and repetitive modes. We also compared the information contents of the M1 motifs of the Common peaks with their best matching motifs from the Perm-seq-exclusive peaks (Fig. S16 in S1 Text). This comparison revealed that the overall information contents of the *de novo* learnt canonical motifs are highly correlated: 0.74 (0.73) when using Common peaks versus 0.80 (0.94) when using the subset of the Common peaks that match the overall signal of the Peak-seq-exclusive peaks in GM12878 (K562) cells. Correlation calculations excluded Zbtb33 for both cell types and Srf for K562 as outliers. For these factors, the motif logos from Perm-seq-exclusive peaks revealed extended versions of the logos from Common peaks and had high information content flanking regions (Fig. S17 and Fig. S18 in S1 Text).

**Fig 4:**
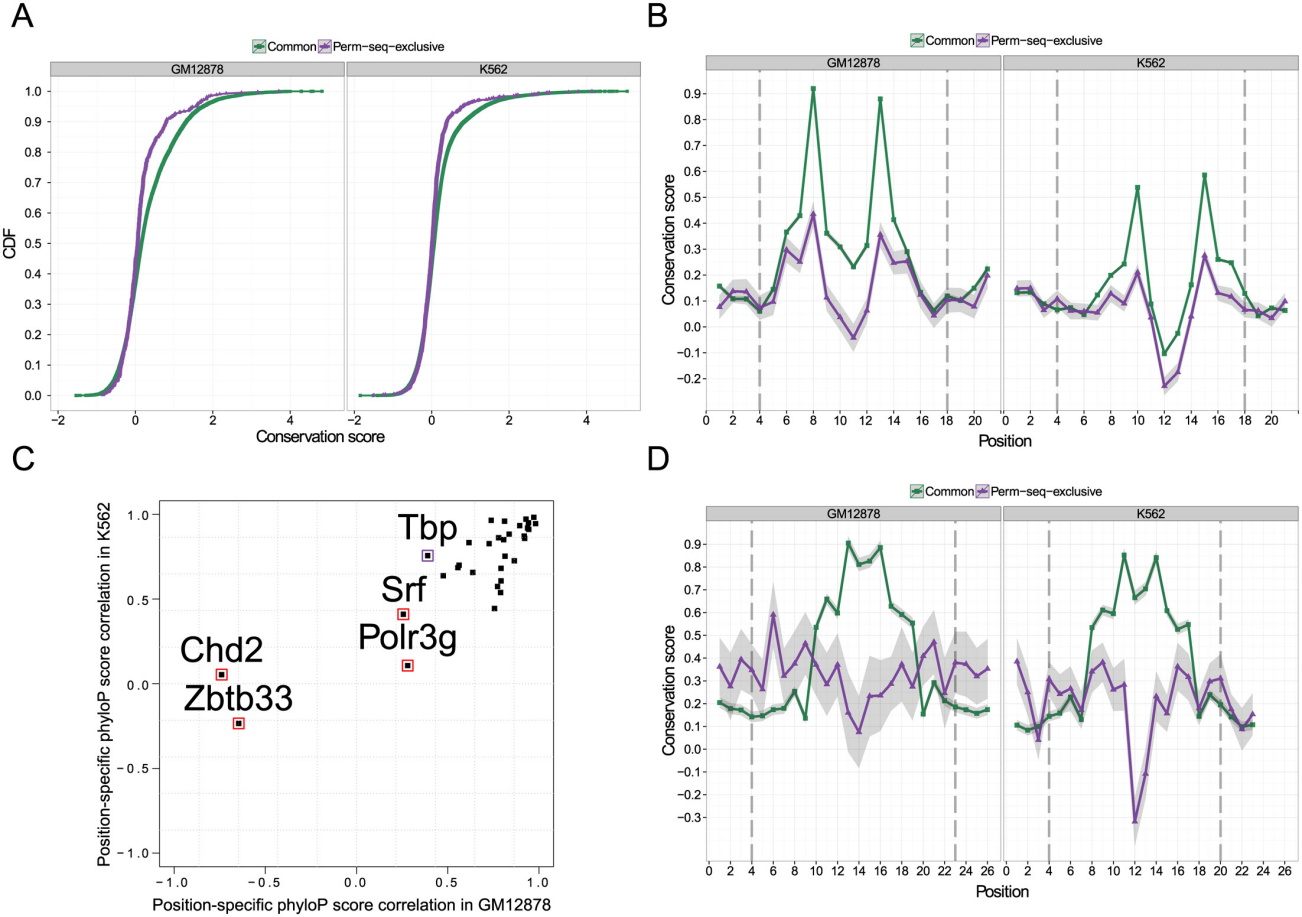
Conservation analysis of the common and Perm-seq-exclusive peak sets. (a) Empirical cumulative distribution functions (CDFs) for average phyloP scores of Usf1 binding sites from the Common and Perm-seq-exclusive Usf1 peaks in GM12878 and K562 cells, respectively. Positive scores indicate conservation and negative scores indicate acceleration. (b) Mean position-specific phyloP scores of the Usf1 binding sites for Common and Perm-seq-exclusive Usf1 peaks in GM12878 and K562, respectively. Shaded areas denote ± one standard error of the mean profile. (c) Pearson correlations between the mean position-specific phyloP scores of the binding sites from the Common and Perm-seq-exclusive peak sets. Purple and red circles indicate that correlations are not significantly different than zero only in GM12878 and in both GM12878 and K562 cells, respectively. (d) Mean position-specific phyloP scores of the Zbtb33 binding sites from the Common and Perm-seq-exclusive Zbtb33 peaks in GM12878 and K562 cells, respectively.

Having established that binding sites from the repetitive mode exhibit similar sequence contents to those of the binding sites in the non-repetitive mode, we evaluated evolutionary conservation of the binding sites from the two groups. Specifically, we took all the best matches to the M1 motif of the Common peak set from both of the Common and Perm-seq-exclusive peak sets and analyzed their phyloP score [32] distributions using the pre-computed phyloP scores from the UCSC Genome Browser. Fig 4(a) compares the phyloP conservation scores averaged over the individual binding sites within each group with an empirical cumulative distribution function plot for transcription factor Usf1. The observed pattern indicates that Perm-seq-exclusive binding sites are overall less conserved. A similar result holds for the majority of the factors (S4 Text & S5 Text). A Wilcoxon-rank sum test for each of the 32 factors revealed that only four (Jund, Spi1, Srf, Tbp) and six (Max, Nfe2, Polr2a, Rest, Sin3a, Spi1) factors do not have significantly different conservation levels (adjusted p-value larger than 0.05 according to the Benjamini-Hocberg false discovery rate (FDR) control [33]) between the Common and Perm-seq-exclusive binding sites in GM12878 and K562 cells, respectively. We next considered position-specific (nucleotide level) phyloP scores. Fig 4(b) displays average position-specific phyloP scores for binding sites of Common and Perm-seq-exclusive peaks of Usf1. Although the overall nucleotide-level conservation scores for the Perm-seq-exclusive sites are lower than those of the Common sites, the patterns of the mean profiles are very similar and highly correlated (Pearson correlations are 0.81 and 0.85 in GM12878 and K562 cells, respectively). Furthermore, nucleotides within the motif (between vertical dashed lines) tend to have higher scores than those in the adjacent non-motif positions for the Perm-seq-exclusive binding sites. We next tested the correlations between average position-specific phyloP profiles of the Common and Perm-seq-exclusive peaks (Fig 4(c)). All but 5 factors (Chd2, Polr3g, Srf, Tbp, Zbtb33) have significantly correlated mean profiles of position-specific phyloP scores between the repetitive and non-repetitive modes of binding in both of the cell lines. Of the five factors with low correlation, Tbp is conserved in moderate levels in both GM12878 and K562 but does not pass the significance cut-off in GM12878 (adjusted p-value of 0.051). Profiles for the other four factors exhibit starker differences for the Common and Perm-seq-exclusive peaks. Neither Chd2 nor Polr3g are sequence-specific factors and their M1 motifs from the Common set are not identified *de novo* from the analyses of the Perm-seq-exclusive peaks. Serum response factor Srf is a sequence-specific transcription factor. The canonical motif for this factor is identified as the forth significant motif in GM12878 and as the first in K562 Perm-seq-exclusive peak sets. Although the correlations between conservation profiles do not achieve the significance cut-off, they exhibit reasonable correlation (0.41 in K562 with an adjusted p-value of 0.06 and 0.26 in GM12878 with an adjusted p-value of 0.17). Furthermore, there are not notable differences between the canonical motifs identified from the repetitive and non-repetitive regions of binding (Fig. S17 in S1 Text). Zbtb33 is a transcriptional regulator with bimodal DNA-binding specificity and is known to bind to methylated CGCG and the non-methylated consensus site TCCTGCNA [34]. The position-specific conservation profiles between the Common and Perm-seq-exclusive peak sets exhibit significant lack of correlation, especially at the CGCG core (Fig 4(d)). The “G” nucleotide of the first CpG in the core shows accelerated evolution with a negative phyloP score in K562 and the sequence logos of both of the motifs from GM12878 and K562 Perm-seq-exclusive peaks reveal a degenerate position at this location of the “CGCG” core (Fig. S18 in S1 Text). This raises the possibility of Zbtb33 interaction with unmethylated or partially methylated CGCG. To assess this, we calculated the fraction of the Common peaks that exhibited methylation at the CGCG core. Comparisons with the ENCODE Reduced Representation Biosulphite Sequencing (RRBS) data revealed that only 26.3% and 17.2% of CGCGs were methylated in the Common peaks with at least one CGCG core, in GM12878 and K562 cells respectively. This result supports Zbtb33 interaction with unmethylated CGCGs and the possibility of a more degenerate CGCG core for the Zbtb33 motif in the repetitive mode.

## Perm-seq with with multiple sources of data

The core of the Perm-seq methodology relies on the innovation of incorporating DNase-seq data to derive priors for read allocation probabilities with a Dirichlet-multinomial regression model. This regression framework is highly versatile and can easily accommodate incorporation of other epigenomic data for prior construction. Various histone modifications contribute different putative functions to gene regulation. For example, acetylation of H3k27 and H3k9 are associated with gene activation, trimethylations of H3k27 and H3k9 are linked to repression and H3k4me1, H3k4me2, and H3k4me3 are correlated with functional enhancers/promoters in various cell types [35, 36].

Perm-seq incorporates multiple histone datasets as additional covariates in the Dirichlet-multinomial regression model and employs a variable selection procedure with Group Lasso [37] to select the histones that are most relevant for read allocation (work-flow in Fig. S19 of S1 Text).

### Perm-seq analysis with histone ChIP-seq in addition to DNase-seq provides further discriminating power between read mapping positions

We reanalyzed a subset of the ENCODE ChIP-seq datasets (Atf3, Ctcf, Rest, Sin3a, Egr1, Ep300 from GM12878 cells) by utilizing 11 histone ChIP-seq datasets (H2a.z, H3k27ac, H3k27me3, H3k36me3, H3k4me1, H3k4me2, H3k4me3, H3k79me2, H3k9ac, H3k9me3, H4k20me1). In this set, Atf3 and Ctcf function as both transcriptional activators and repressors, Rest and Sin3a are repressors, and Egr1 and Ep300 are activators [38]. Table S6 in S1 Text lists the final set of histone marks that were selected for each TF. We observe that histone variant H2a.z and modifications H3k27ac and H3k27me3 significantly contributed to prior construction for at least five of the six datasets. Overall, inclusion of multiple histone data in prior construction resulted in an increase of numbers of peaks for Atf3, Ctcf, and Ep300 and a decrease for the rest of the factors compared to Perm-seq analysis with only DNase-seq (Table S7 in S1 Text). On average, the change in the numbers of peaks with the inclusion of histone datasets were much smaller (0.99%) compared to the overall change in the numbers of peaks with and without prior information (4.6%) and with and without multi-reads (8%).

**Fig 5:**
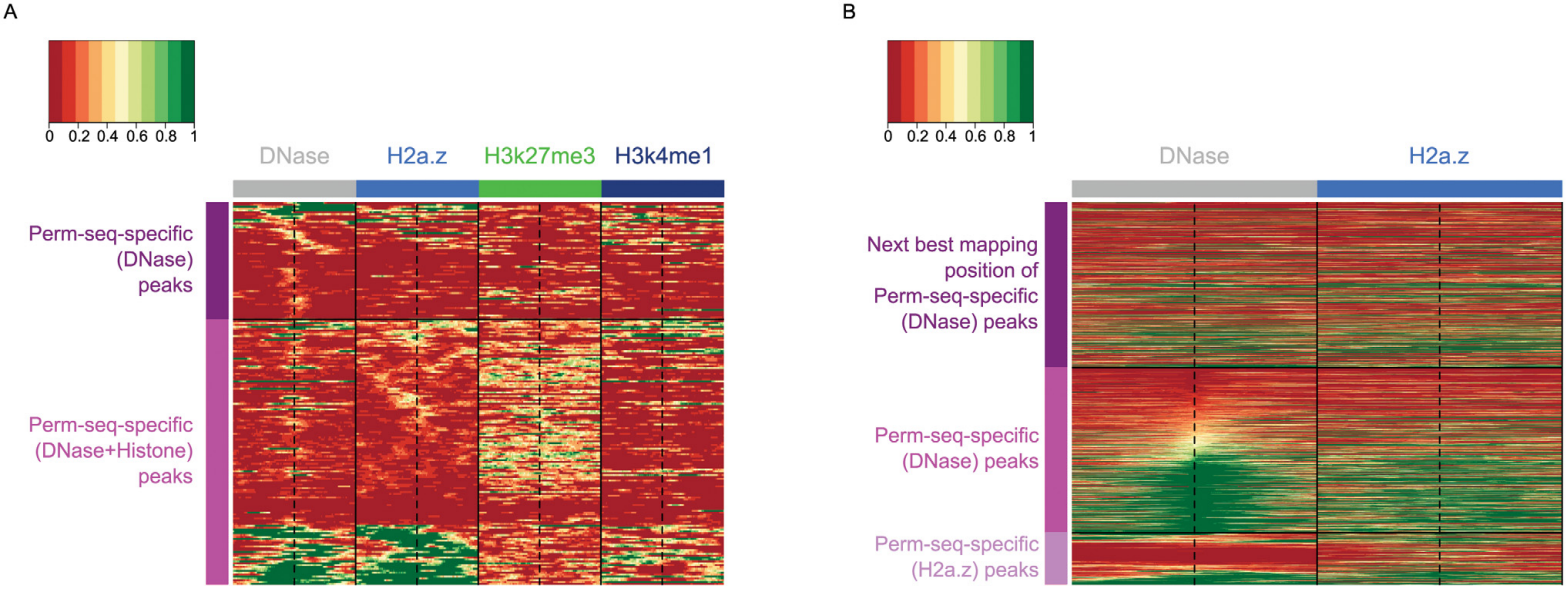
Perm-seq analysis of GM12878 Ctcf and Sin3a ChIP-seq dataset with DNase-seq and Histone ChIP-seq priors. (a) Heatmap of normalized DNase-seq and Histone ChIP-seq read counts for the [-1000 bps, +1000 bps] window anchored at GM12878 Ctcf peak summits depicted by the vertical dashed lines. Perm-seq-specific (DNase+Histone): Perm-seq optimal peaks using DNase-seq and Histone ChIP-seq in prior construction. These peaks are not identified when using only DNase-seq for prior construction or with CSEM. Perm-seq-specific (DNase): Perm-seq optimal peaks using only DNase-seq in prior construction. These peaks are not identified when using DNase-seq and Histone ChIP-seq for prior construction or with CSEM. (b) Heatmap of normalized DNase-seq and H2a.z ChIP-seq read counts for the [-500 bps, +500 bps] window anchored at GM12878 Sin3a peak summits. Perm-seq-specific (DNase): Perm-seq optimal peaks using DNase-seq in prior construction. These peaks are not identified when using only H2a.z ChIP-seq for prior construction or with CSEM. Perm-seq-specific (H2a.z): Perm-seq optimal peaks using only H2a.z ChIP-seq in prior construction. These peaks are not identified when using DNase-seq for prior construction or with CSEM. Next best mapping positions of Perm-seq-specific (DNase) peaks denote the regions where the multi-reads of the peaks map with the next best total allocation scores, i.e., ranked second compared to the allocation scores at the actual peaks.

We performed detailed analysis of Perm-seq peaks obtained with DNase alone and DNase and Histone ChIP-seq and did not observe significant differences between the motif occurrence percentages in these peak sets. Fig 5(a) displays the DNase and Histone modification profiles of Ctcf peaks in the Perm-seq analysis. Utilizing histone ChIP-seq in addition to DNase-seq eliminates peaks with low DNase-seq read counts and lacking histone support. The majority of Ctcf Perm-seq-specific (DNase) peaks are located within low DNase and low histone signal regions, with an average DNase read count of 18.94 and average histone read count of 5 (Fig 5(a) (upper panel)). Incorporation of histone datasets identifies novel peaks supported by histone profiles. For the Perm-seq-specific (DNase+Histone) peaks, the average DNase read count is 30.18 and average H2a.z count is 19.51. Further investigation of these regions indicate that additional support from the histone profiles enable discrimination between mapping positions with similar DNase-seq profiles. Fig. S20 in S1 Text provides circos plots of read allocation by Perm-seq-specific (DNase) and Perm-seq-specific (DNase+Histone) for a Perm-seq-specific (DNase+Histone) peak with reads distributed over two segmental duplication regions and elucidates how additional histone information is guiding read allocation. The two regions depicted in the circos plots span partially overlapping segmental duplication regions (chr1: 83,647,856–83,955,427 and chr7: 76,280,701–76,575,579). Multi-read allocation by Perm-seq using only DNase data fails to distribute the set of multi-reads to a single region because of low DNase read counts in both of the regions and, hence, does not identify a peak in any of them. However, as depicted with the H2a.z and H3k27ac tracks (the second and third tracks), only one of these two regions has considerable H2a.z, a variant of histone H2a associated with regulatory elements within dynamic chromatin [39], and H3k27ac, mark of active regulatory elements. Utilizing histone information allocates majority of the multi-reads to the region supported by histone information and identifies a peak with a motif.

We next explored Egr1 Perm-seq (DNase) specific peaks. Most of these peaks show both strong DNase and histone signals (Fig. S21 in S1 Text), yet they are not identified by the Perm-seq (DNase+Histone) analysis. We compared the DNase and histone signals of these peak regions with the signal in the next best alternative mapping regions (mapping regions with the second largest allocation weights compared to peaks). In Fig. S21 of S1 Text, we observe histone signal both in the next best alternative mapping regions (left panel) and peak regions (right panel). Furthermore, very few peak regions have DNase and histone signal consistently higher than those of the next best possible mapping regions, leading to Perm-seq analysis with both DNase and Histone to miss these regions.

#### Perm-seq with H2a.z ChIP-seq performs comparatively to Perm-seq with DNase-seq

We next sought to understand whether the information contributed by DNase can be captured by any single histone modification. Since H2a.z was selected in prior construction for all the six factors (Table S6 in S1 Text) and its marginal association with ChIP read counts was most similar to that of the DNase read counts with the ChIP read counts (Fig. S22 in S1 Text), we compared Perm-seq with H2a.z ChIP-seq versus DNase-seq for prior construction. Overall, both data sets as priors led to similar numbers of peaks with Perm-seq (Table S8 in S1 Text). Furthermore, the set of common peaks identified by using either of the data types as prior was generally larger than the peak sets specific to either of the priors for all the TFs except Sin3a. We next investigated the peak set differences. In general, more Perm-seq-specific (DNase) peaks are associated with strong DNase signal. We defined regions with read counts larger than the 99-th percentile of the read count distribution as strong signal regions. On average, 46% (with a standard deviation of 18.5%) of Perm-seq-specific (DNase) peaks are within strong DNase signal regions compared to 25% (with a standard deviation of 17.1%) within strong H2a.z regions. In contrast, on average 42.5% (with a standard deviation of 11.8%) of the Perm-seq-specific (H2a.z) peaks are associated with strong H2a.z signal while only 27.2% (with a standard deviation of 9.1%) are associated with strong DNase signal. This suggests that peaks in the set differences are similarly supported by their respective data types used for prior construction.

We next focused on Sin3a since Perm-seq peaks with DNase and H2a.z priors differ the most for this factor. Surprisingly, the percentage of Perm-seq-specific (DNase) peaks residing in strong H2a.z signal regions is 47.0%, as high as the percentage of peaks residing in strong DNase signal regions, which is 54.8%. Fig 5(b) (top 2 panels) displays H2a.z and DNase profiles for Sin3a Perm-seq-specific (DNase) peaks and the next best mapping regions (with second largest allocation probability of the reads). We observe that peaks and the next best alternative regions show comparable H2a.z signal while only the peaks exhibit much stronger DNase signal. Therefore, Perm-seq using DNase prior successfully allocates multi-reads to the peaks while H2a.z fails to discriminate between the two mapping regions. Majority of the Perm-seq-specific (H2a.z) peaks have low DNase read counts as depicted in Fig 5(b) (third panel) and, therefore, cannot be identified by Perm-seq with DNase prior. For other TFs, the peak lists obtained by Perm-seq using different priors are quite consistent. Overall, H2a.z ChIP-seq seems to be an equally powerful alternative to DNase-seq for allocating multi-reads with prior information.

## Discussion

Understanding the nature of protein-DNA interactions in highly repetitive regions of the genome remains a major challenge in deciphering the human transcriptional regulatory code. To expand the capability of ChIP-seq experiments for profiling repetitive regions of genomes, we developed a prior-enhanced read mapping method named Perm-seq that capitalizes on DNase-seq and histone ChIP-seq data and read counts of local neighbourhoods of mapping locations to discriminate between multiple alignment positions of the same read. Perm-seq analysis of a large collection of ENCODE ChIP-seq datasets from GM12878 and K562 cells revealed that Perm-seq leads to higher sequencing depths and, more importantly, identification of novel targets in segmental duplication regions as well as other repetitive regions. We found that these novel targets, to a large extent, share the same sequence characteristics of the known canonical binding sites of the studied TFs. Conservation analysis indicated that canonical binding sites in these regions are evolutionary less conserved compared to their counterparts in non-repetitive regions; however, the patterns of nucleotide-level conservations correlate well between the repetitive and non-repetitive region binding sites. By integrating ChIP-seq data of Pol2 with RNA-seq data, we found that many highly expressed genes originally thought not have Pol2 occupancy in the promoter regions with uni-read analysis of ChIP-seq data indeed have Pol2 peaks revealed by Perm-seq analysis.

Perm-seq assumes that DNase-seq data harbors information regarding the nature of the protein-DNA interactions and the prior building step learns the specifics of this relationship. For most transcription factors, protein-DNA interactions occur in regions of accessible chromatin. However, a number of proteins, e.g., KRAB-associated factors) are observed to bind to inaccessible chromatin [40]. Exploratory plots such as Fig. S2 of S1 Text indicate whether DNase-seq priors are informative. If this plot does not reveal a systematic relationship between the ChIP and DNase counts, the prior building step generates non-informative priors and, hence, Perm-seq is not expected to improve over methods that do not utilize priors. We also explored the use of multiple histone ChIP-seq datasets in addition to DNase-seq for constructing priors. Although we observed that histone ChIP-seq, in addition to DNase-seq, could further discriminate between the mapping positions, the gain due to using additional histone ChIP-seq was not as large as that of the gain due to using DNase-seq as prior as opposed to no priors. Furthermore, H2a.z ChIP-seq performed as a comparable alternative to DNase-seq for prior construction.

The DNase-seq samples we used have moderate sequencing depths (approximately 61M aligned reads for Huvec, 63M for GM12878, and 49M for K562). We observe that, as expected, the discriminative power of DNase-seq attenuates with lower depths as illustrated in Fig. S23 of S1 Text. Therefore, we recommend prior construction with high quality DNase-seq data with at least 15–20M reads as recommended by the ENCODE consortium [24]. Finally, we focused on read allocation for TF ChIP-seq experiments. The overall framework should also be useful for multi-read allocation of ChIP-experiments for punctuated histone marks.

The current implementation of the Perm-seq pipeline is available at https://github.com/keleslab/permseq.

## Materials and Methods

### Perm-seq generative model

The initial step of Perm-seq is to align reads to the reference genome with a standard aligner that can report both uni- and multi-mapping reads. We utilized Bowtie [23] for this task and retained reads with at most 2 mismatches and fewer than 100 reported alignments. These initial aligned set of reads were then modelled with the Perm-seq generative model to probabilistically allocate multi-mapping reads to their mapping locations using DNase-seq data as a prior. We next provide mathematical details of the Perm-seq model.

Let *N* be the number of reads, i.e., sequencing depth, and *M* be the number of genomic positions. Let *i* = 1, …, *N* denote the index for reads, *j* = 1, …, *M* for positions. Let **Z**_*i*_ = (*Z*_*i*1_, …, *Z*_*iM*_) be the true origin indicator for the *i*-th read. If the *i*-th read is generated from *j*-th position, then *Z*_*ij*_ = 1 and *Z*_*ij*′_ = 0, for *j*′ ≠ *j*. We assume that {**Z**_*i*_, *i* = 1, …, *N*} are independent and from a multinomial distribution with parameter vector *π* = (*π*_1_, …, *π*_*M*_). Here, *π* is the density function for generating reads, and specifically, *P*(*Z*_*ij*_ = 1) = *π*_*j*_. In this generative model, **Z**_*i*_s are not directly observable since multi-reads can align to multiple locations on the reference genome. Hence, **Z**_*i*_ are hidden random variables. As a result of alignment, we observe binary vectors **Y**_*i*_ = (*Y*_*i*1_, …, *Y*_*iM*_) where *Y*_*ij*_ = 1 if *i*-th read is aligned to *j*-th position on the genome and *Y*_*ij*_ = 0 otherwise. If the *i*-th read is a uni-read, then $\sum_{j=1}^{M}Y_{ij}=1$. If the *i*-th read aligns to *k* different positions with *k* > 1, then $\sum_{j=1}^{M}Y_{ij}=k$. For both uni- and multi-reads, $\sum_{j=1}^{M}Z_{ij}=1$. Defining *L*_*i*_ = {*j*:*Z*_*ij*_ = 1} as the actual position read *i* originates from and *h*_*ij*_ = *Pr*(*Y*_*i*_ ∣ *L*_*i*_ = *j*), we have $Pr\left(Y_{i},Z_{i}\right)=\prod_{j=1}^{M}{\left(h_{ij}\pi_{j}\right)}^{Z_{ij}}$. Under the assumption that the true origin *L*_*i*_ is one of the observed alignment positions, we have *h*_*ij*_ = *Y*_*ij*_.

To utilize DNase-seq data, we assign a Dirichlet prior distribution to ***π*** with the following density function:

$$\begin{array}{c} f_{D}\left(\pi ;\gamma \right)=\frac{\Gamma (\gamma_{+})}{\prod_{j=1}^{M}\Gamma \left(\gamma_{j}\right)}\;\prod_{j=1}^{M}\pi_{j}^{\gamma_{j}-1}, \end{array}$$ (1)

where Γ(.) is the Gamma function, $\gamma_{+}=\sum_{j=1}^{M}\gamma_{j}$, and $\sum_{j=1}^{M}\pi_{j}=1$. Then, we define intermediate variables *S*_*j*_, *S*_*j*_ ≥ 0 as the pseudo counts added to position *j*, *j* = 1, ⋯, *M* and set *γ*_*j*_ = *S*_*j*_ + 1, which enforces *γ*_*j*_ ≥ 1 to avoid negative component values in the maximization (M) step in estimation of the allocation probabilities.

DNase-seq data informs the prior parameters with a Dirichlet-multinomial regression by assuming that *S*_*j*_ depends on DNase read counts *x*_*j*_ via the following log-linear model with B-splines:

$$\begin{array}{c} S_{j}|x_{j}∼\exp[\beta_{0}+\beta_{1}SP\left(x_{j}\right)],j=1,\ldots ,M, \end{array}$$ (2)

where ***SP***(.) is a vector of piece-wise linear B-spline basis functions and *β*_1_ are the set of associated parameters. Let ***x*** = (*x*_1_, …, *x*_*M*_). Then, we can rewrite Eq (1) by conditioning on *x* as:

$$\begin{array}{c} f_{D}\left(\pi ;\beta_{0},\beta_{1},|x\right)=\frac{\Gamma \{M+\sum_{j=1}^{M}\exp[\beta_{0}+\beta_{1}SP\left(x_{j}\right)]\}}{\prod_{j=1}^{M}\Gamma \{1+\exp[\beta_{0}+\beta_{1}SP\left(x_{j}\right)]\}}\prod_{j=1}^{M}\pi_{j}^{\exp[\beta_{0}+\beta_{1}SP\left(x_{j}\right)]}. \end{array}$$ (3)

When incorporating multiple histones ChIP-seq datasets, we first convert histone read counts into trinary enrichment indicators indicating low, median, and strong signal strengths. Specifically, for a given histone ChIP-seq dataset, positions with read counts smaller than 90-th percentile of the overall read count distribution are set as non-enriched (0), positions with read counts smaller than 99-th percentile but larger than 90-th percentile are set as moderately-enriched (1), and positions with read counts larger than the 99-th percentile are set as enriched (2). Unlike DNase-seq which measures the extent of chromatin accessibility, histone ChIP-seq identifies regions exhibiting histone modifications, i.e., the true underlying states in a histone ChIP-seq experiment is modified versus unmodified as opposed to a continuous scale. The inclusion of a moderately-enriched group is due to the fact that the multi-read only peaks, in general, exhibit lower signal for all the modifications but their average modification profiles match to those of common peaks that can be identified by both uni- and multi-reads as illustrated in Fig 3(c) and S2 Text & S3 Text.

Let *N*_*h*_ denote the number of histone ChIP-seq datasets and define **h**_*j*_ = (**h**_1*j*_, …, **h**_*N_h_ j*_), where **h**_*ij*_ is the dummy variable of i-th histone’s trinary indicator at j-th position. Let **h** = (**h**_1_, …, **h**_*M*_) and ***β***_2_ be a vector valued parameter associated with the histone ChIP-seq datasets. Then, Eq (3) can be extended as:

$$\begin{array}{c} f\left(\pi ;\beta_{0},\beta_{1},\beta_{2},x,h\right)=\frac{\Gamma \{M+\sum_{j=1}^{M}\exp[\beta_{0}+\beta_{1}\text{SP}\left(x_{j}\right)+\beta_{2}h_{j}]\}}{\prod_{j=1}^{M}\Gamma \{1+\exp[\beta_{0}+\beta_{1}\text{SP}\left(x_{j}\right)+\beta_{2}h_{j}]\}}\prod_{j=1}^{M}\pi_{j}^{\exp[\beta_{0}+\beta_{1}\text{SP}\left(x_{j}\right)+\beta_{2}h_{j}]}. \end{array}$$ (4)

## Parameter estimation in the Perm-seq model

Theoretically, ***π***, *β*_0_, and ***β*_1_** can be estimated simultaneously via the Expectation(E)-Maximization(M)-Smoothing(S) algorithm [41]. However, since prior parameters *β*_0_ and ***β*_1_** do not have closed form estimators, simultaneous estimation of all three parameters require repeated numerical optimization in the maximization step of the EMS algorithm. To avoid such high computational cost, we first estimate the prior parameters *β*_0_ and ***β*_1_** and then estimate ***π*** while keeping these prior parameter estimates fixed. Our data-driven computational experiments indicate that this estimation procedure can reliably estimate the read density (Fig. S10(d) in S1 Text).

When estimating *β*_0_ and ***β*_1_**, we first fit the log-linear model (2) using data from positions with only uni-reads with a data aggregation strategy. In the case of uni-reads, *S*_*j*_ can be replaced by the actual ChIP read counts. We group positions 1, …, *M* with the same DNase read counts and average *S*_*j*_ within each group. When using both DNase and histone data, *S*_*j*_ is averaged across positions with the same DNase read counts and combinatorial histone patterns. To avoid the potential estimation bias from regions with ultra-low DNase read counts, we group positions with 0, 1, 2 DNase read counts together. To further decrease computational complexity, we pool positions with ultra-high DNase read counts (larger than the 99.95-th percentile of the DNase read count distribution) together. Then, we put knots at the 90, 99, and 99.9-th percentiles of the averaged DNase read counts and fit the log linear model using these aggregated data. This set of knot points was well supported by our large scale analysis of ENCODE data. When multiple histone ChIP-seq datasets are available for prior construction, we employ Group Lasso [37] on the aggregated data to select the most relevant histone datasets. Once we obtain the estimates $\hat{\beta_{0}}$ and $\hat{\beta_{1}}$, we use the EMS algorithm to estimate ***π*** as follows. Let ***π*** ^(*t*)^ denote the estimate of ***π*** from the *t*-th iteration,

**E-step:** Taking expectation of **Z**_*ij*_ conditional on observed read alignments **Y**, we obtain
  $$\begin{array}{c} z_{ij}^{\left(t\right)}\equiv E_{\pi^{\left(t\right)}}[Z_{ij}|Y=y]=\frac{\pi_{j}^{\left(t\right)}}{\sum_{j^{\prime }\in R_{i}}\pi_{j^{\prime }}^{\left(t\right)}}1\left(j\in R_{i}\right), \end{array}$$
  where *R*_*i*_ is the set of positions that the *i*-th read aligns to and 1(.) is the indicator function.
**M-step:** In the M-step, we obtain an intermediate estimate of *π*_*j*_ denoted by $\mu_{j}^{\left(t+1\right)}$:
  $$\begin{array}{c} \mu_{j}^{\left(t+1\right)}=\frac{\sum_{i}^{N}z_{ij}^{\left(t\right)}+\exp[\hat{\beta_{0}}+\hat{\beta_{1}}SP\left(x_{j}\right)]}{N+\sum_{j=1}^{M}\exp[\hat{\beta_{0}}+\hat{\beta_{1}}SP\left(x_{j}\right)]}. \end{array}$$
**S-step:** In the S-step, we smooth $\mu_{j}^{\left(t+1\right)}$ with a moving average smoother to obtain $\pi_{j}^{\left(t+1\right)}$:
  $$\begin{array}{c} \pi_{j}^{\left(t+1\right)}=\frac{1}{2w+1}\sum_{j^{\prime }=j-w}^{j^{\prime }=j+w}\mu_{j^{\prime }}^{\left(t+1\right)}, \end{array}$$
  where *w* is the half size of the smoothing window. The moving average smoother was also adapted by CSEM where 2*w* + 1 was set to the average library size. This choice was well supported by the computational experiments in CSEM. Estimation with the extended histone ChIP-seq datasets follow the same procedure with the density of *π* given in Eq (4).

### Perm-seq analysis of ENCODE data

All of the data files used in the analysis are listed in Supplementary Tables S8–S10. After processing of all the fastq files with Perm-seq, we assigned each multi-read to its best mapping position if the allocation probability at that position exceeded 0.5, i.e., multi-reads with maximum allocation probability less than 0.5 were discarded. Although this approach is more conservative than utilizing all the multi-mapping reads and all the positions that they are allocated to, it makes the final alignment files compatible with the ENCODE’s ChIP-seq uniform processing pipeline. We used the peak calling and IDR parameters adopted by the ENCODE pipeline and set the IDR to 0.02. This resulted in both optimal peak lists and relaxed peak lists for each factor in both the uni-read and Perm-seq analyses. The Common peak set for each TF is defined as the peaks common to uni-read and Perm-seq optimal peak lists. Perm-seq-only peaks include those that are in the Perm-seq optimal list but not in the optimal list of the uni-read analysis. To identify the set of Perm-seq-only peaks that are extremely unlikely to be identified with the uni-read analysis, we extended the uni-read optimal list with *n*_opt_ peaks to a total of 5*n*_opt_ peaks by taking the top 5*n*_opt_ relaxed peaks. Then, we defined the Perm-seq-exclusive peak sets as the subsets of the Perm-seq-only peak sets that were not part of these relaxed uni-read peak list.

### Sequence analysis of peaks

Sequence analysis of the Common and Perm-seq-exclusive peak sets included *de novo* motif finding with the MEME Suite [28] using ± 50 bps of summit of the top 500 peaks. For each peak set, we identified the top 5 motifs (M1 to M5) with the smallest E-values (Fig. S15 in S1 Text). Then, the rest of the peak sets were scanned with the fimo tool of the MEME Suite using the default p-value cut-off of 1e-4 for the occurrences of the identified motifs within sequences spanning ± 150 bps of the peak summits. For evaluating the degeneracy of the motifs identified from Perm-seq-exclusive peaks, we generated a subset of the Common peaks that matched the Perm-seq-exclusive peaks in signal strength reported by the peak caller SPP. This set included all the Common peaks with peak signal strengths within the minimum and third quantile of the signal strength of the Perm-seq-exclusive peaks.

### Information content calculations of the *de novo* learnt motifs

We manually compared the most significant motif (M1) of the Common peak set with the five motifs (M1–M5) from the Perm-seq-exclusive peak set for each TF. If the M1 motifs of the Common peak sets were not identified in the Perm-seq-exclusive motif sets, we considered the canonical motifs (as defined in literature for each factor) identified *de novo* in the Common peak sets. The factors for which neither the M1 nor the canonical motif from the Common peak sets were identified the among the M1–M5 of the Perm-seq-exclusive peak sets were excluded from the information content analysis. Then, for each motif pair (M1 or canonical from the Common peak set and the corresponding motif from the Perm-seq-exclusive peak set for each TF), we identified the longest sub-motifs with the highest sequence similarity and extended the flanking bases of the sub-motifs to either the full motif lengths or the first non-degenerate position that did not match between the motifs. The information content of each extended sub-motif was calculated [42] for comparison.

### ChIP-seq aggregation profiles of histone modifications

Histone ChIP-seq datasets were processed with CSEM and reads with allocation probability larger than 0.9 were utilized after being extended to the average library size of 200 bps to calculate aggregation profiles. For each genomic coordinate around the ± 2000 bps of the peak summit, the number of extended reads within a 151 bps window were averaged to generate a smooth aggregation profile. Each profile was then normalized to 1 million reads and the profiles for a given peak set were averaged coordinate-wise. The profile plots were generated using the Segvis R package (https://github.com/keleslab/Segvis).

### Software availability

The perm-seq algorithm is implemented as an R package named permseq and is freely available from https://github.com/keleslab/permseq. The raw datafiles listed in Tables S9, S10, and S11 in S1 Text are available at the ENCODE portal of the UCSC Genome Browser (http://hgdownload.cse.ucsc.edu/goldenpath/hg19/encodeDCC/). Perm-seq results are available at ftp://ftp.cs.wisc.edu/pub/users/kelesgroup/encode2-perm-seq-peaks/.

## Supporting Information

S1 Text Supplementary Methods for “Mapping protein-DNA interactions in segmental duplication and highly repetitive regions of genomes with prior-enhanced read mapping”. (PDF)

S2 Text Supplementary Files for Histone Profiles of Atf3 to Nrf1. (PDF)

S3 Text Supplementary Files for Histone Profiles of Polr2a to Zbtb33. (PDF)

S4 Text Supplementary Files for phyloP Conservation Profiles of Atf3 to Rad21. (PDF)

S5 Text Supplementary Files for phyloP Conservation Profiles of Rest to Zbtb33. (PDF)
