# Supplementary material for: Perm-seq: Mapping Protein-DNA Interactions in Segmental Duplication and Highly Repetitive Regions of Genomes with Prior-Enhanced Read Mapping
Source: PLoS Comput Biol. 2015 Oct 20;11(10):e1004491. doi: 10.1371/journal.pcbi.1004491 (PMC4618727; doi:10.1371/journal.pcbi.1004491)
Supplement: S1 Text — (PDF) [file pcbi.1004491.s001.pdf]

Supplementary Materials for "Mapping protein-DNA interactions in segmental duplication and highly repetitive regions of genomes with prior-enhanced read mapping"

Xin Zeng[1], Bo Li[3], Rene Welch[1], M. Constanza Rojo-Alfaro*[1], Ye Zheng*[1], Colin Dewey[2] & Sündüz Keleş[1,2]
[1] Department of Statistics, University of Wisconsin, Madison, WI, U.S.A.
[2] Department of Biostatistics and Medical Informatics, University of Wisconsin, Madison, WI, U.S.A.
[3] California Institute for Quantitative Biosciences, University of California, Berkeley, CA, U.S.A.

June 24, 2015

1 Discriminative power of DNase-seq for allocating multi-reads in ChIP-seq data

*Equal Contribution

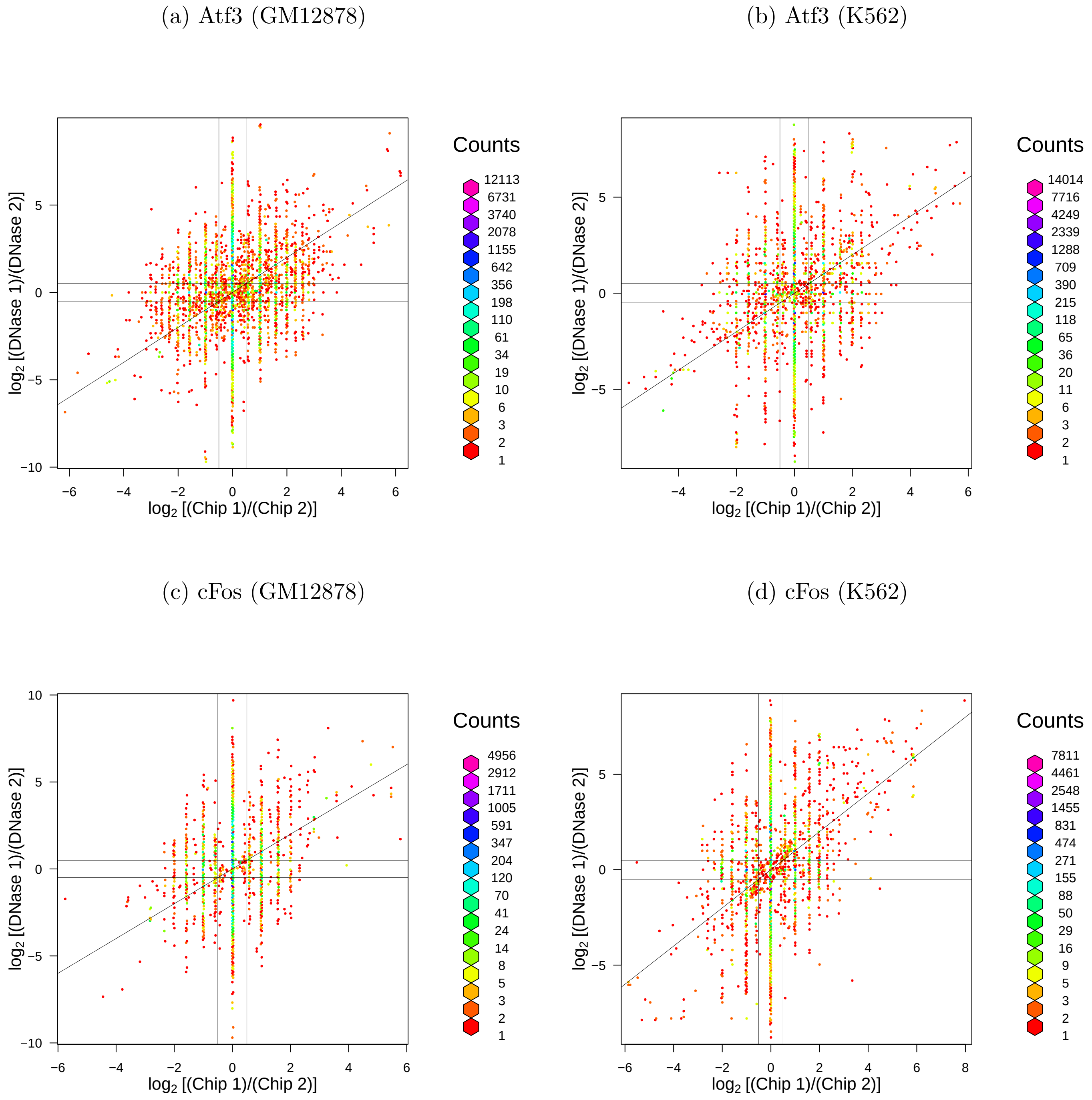

Supplementary Figure 1. Discriminative power of DNase-seq for mapping locations of multi-reads. Log base two ratios of DNase-seq versus ChIP-seq reads counts in the local neighbourhoods of the two mapping locations of each multi-read in Atf3 and cFos ChIP-seq datasets from GM12878 and K562 cells. The vertical and horizontal lines depict boundaries with the log base two ratios equal to 0.5.

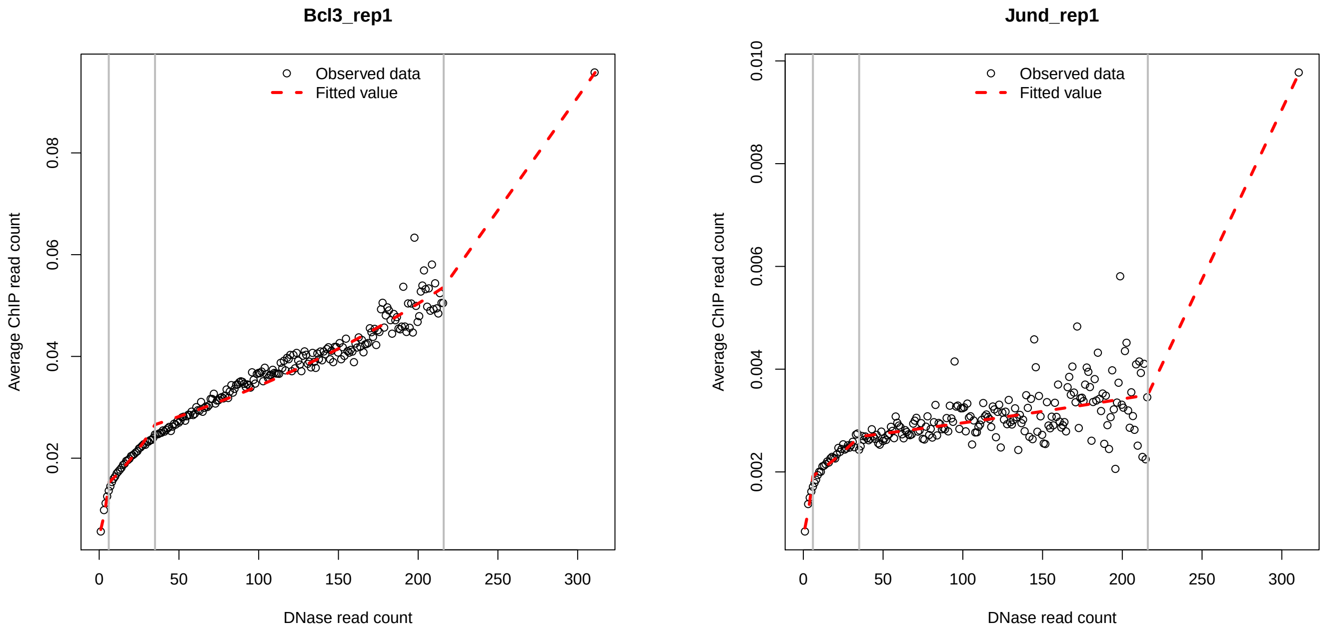

Supplementary Figure 2. DNase versus ChIP read counts for replicate 1 of GM12878 (a) Bcl3 (b) Jund ChIP-seq datasets. Genomic positions with the same DNase read counts are grouped together and ChIP read counts are averaged within each group. Vertical lines indicate the knot points at the 90, 99, and 99.99-th percentiles of the DNase read count distribution.

(a) GM12878

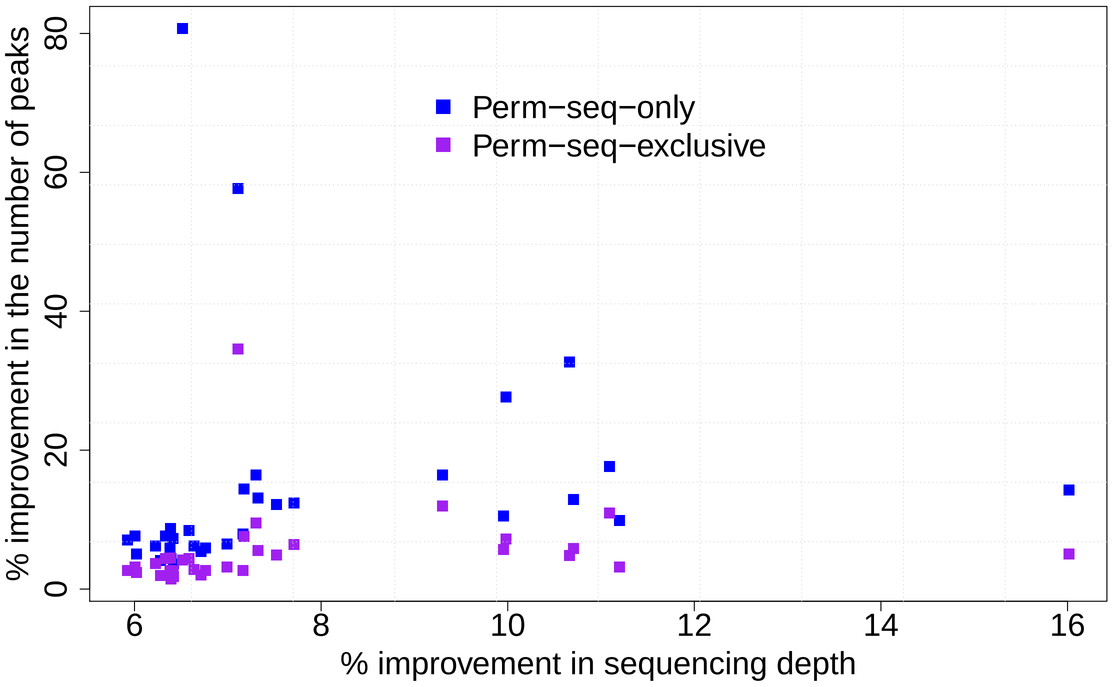

(b) K562

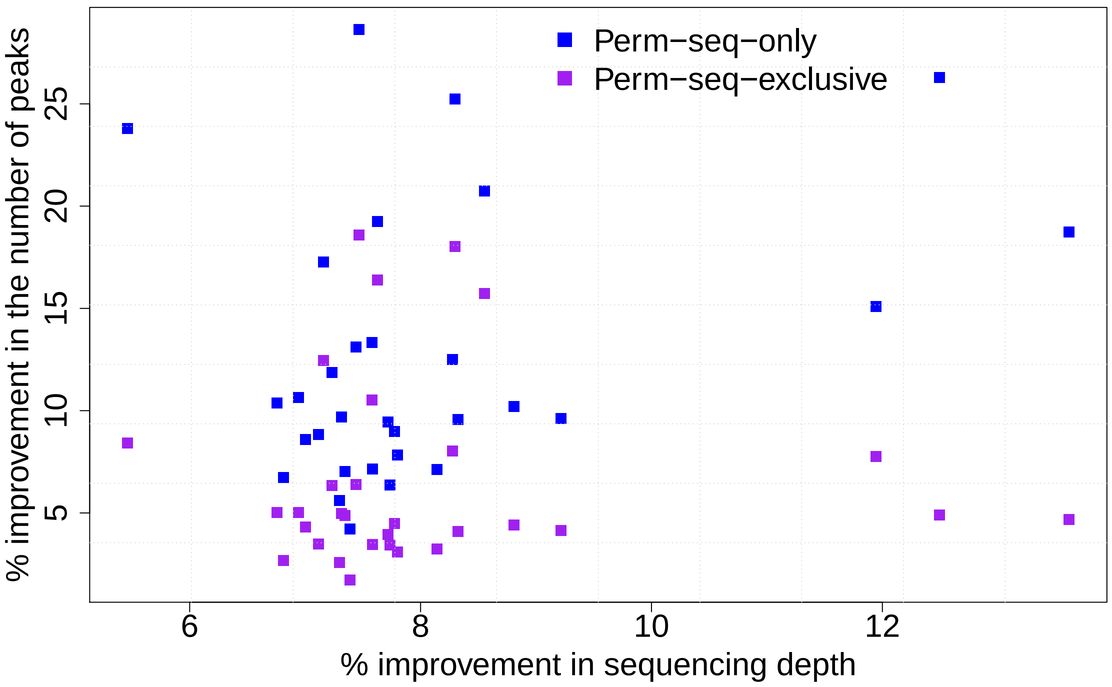

Supplementary Figure 3. Perm-seq improvements on the sequencing depths and the numbers of peaks compared to uni-read analysis for (a) GM12878 and (b) K562 cells. x-axis reports the percentage increase in the sequencing depth due to Perm-seq for each of the 32 factors. y-axis reports the percentage increase in the number of peaks by dividing the number of peaks that are only identifiable by Perm-seq but not uni-read analysis by the total number of peaks from the uni-read analysis. "Perm-seq-only" compares the uni-read peaks at an IDR of 2% with the Perm-seq peaks at the same IDR. "Perm-seq" exclusive compares Perm-seq peaks at an IDR of 2% with an extended uni-read peak list that is 5 times the size of the optimal uni-read optimal peak list as defined at the IDR of 2%.

2 Example Ctcf peaks

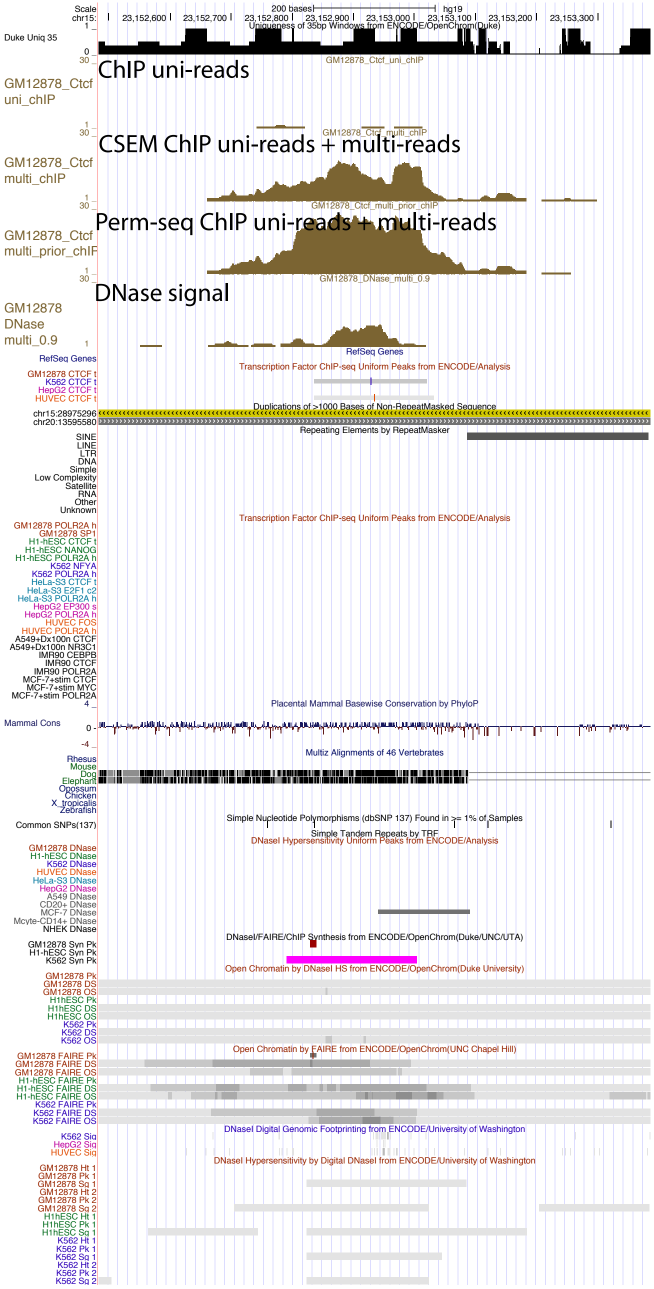

Supplementary Figure 4. UCSC Genome browser screen shot of a sample Ctcf peak in GM12878 cells (chr15:23,152,776-23,153,076) identified by CSEM and Perm-seq and missed by uni-read analysis.

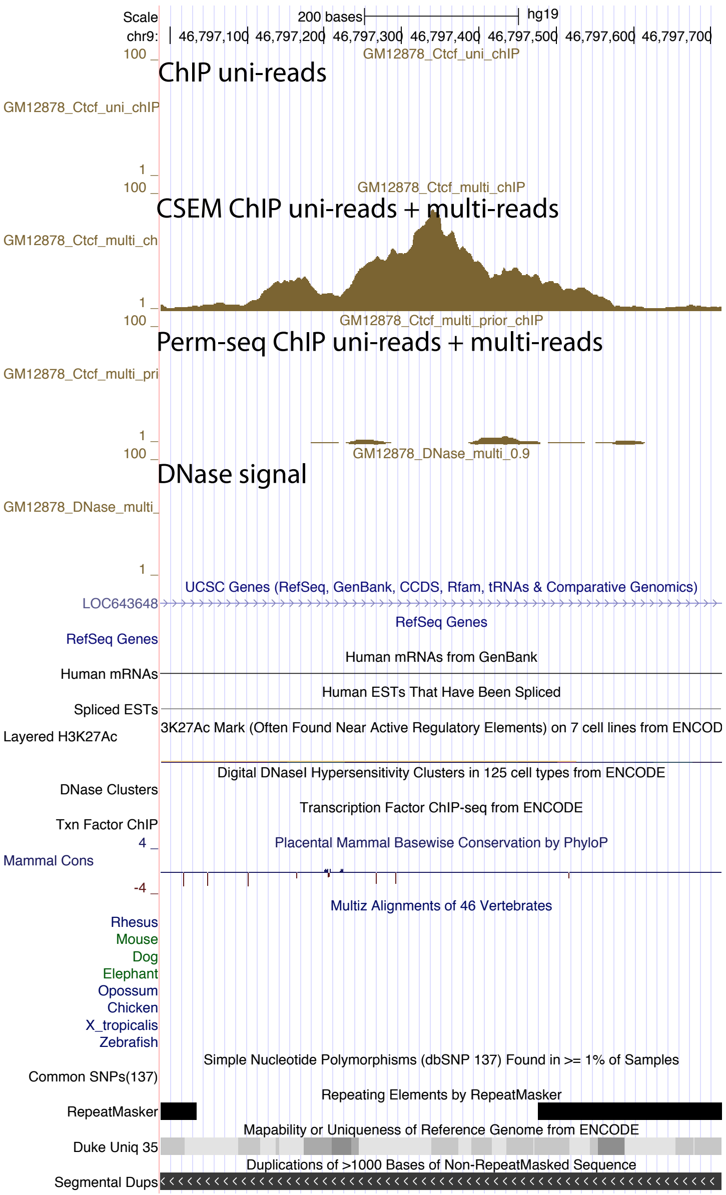

Supplementary Figure 5. UCSC Genome browser screen shot of a CSEM Ctcf peak in GM12878 cells (chr9:46797231-46797472) identified only by CSEM and not supported by DNase data.

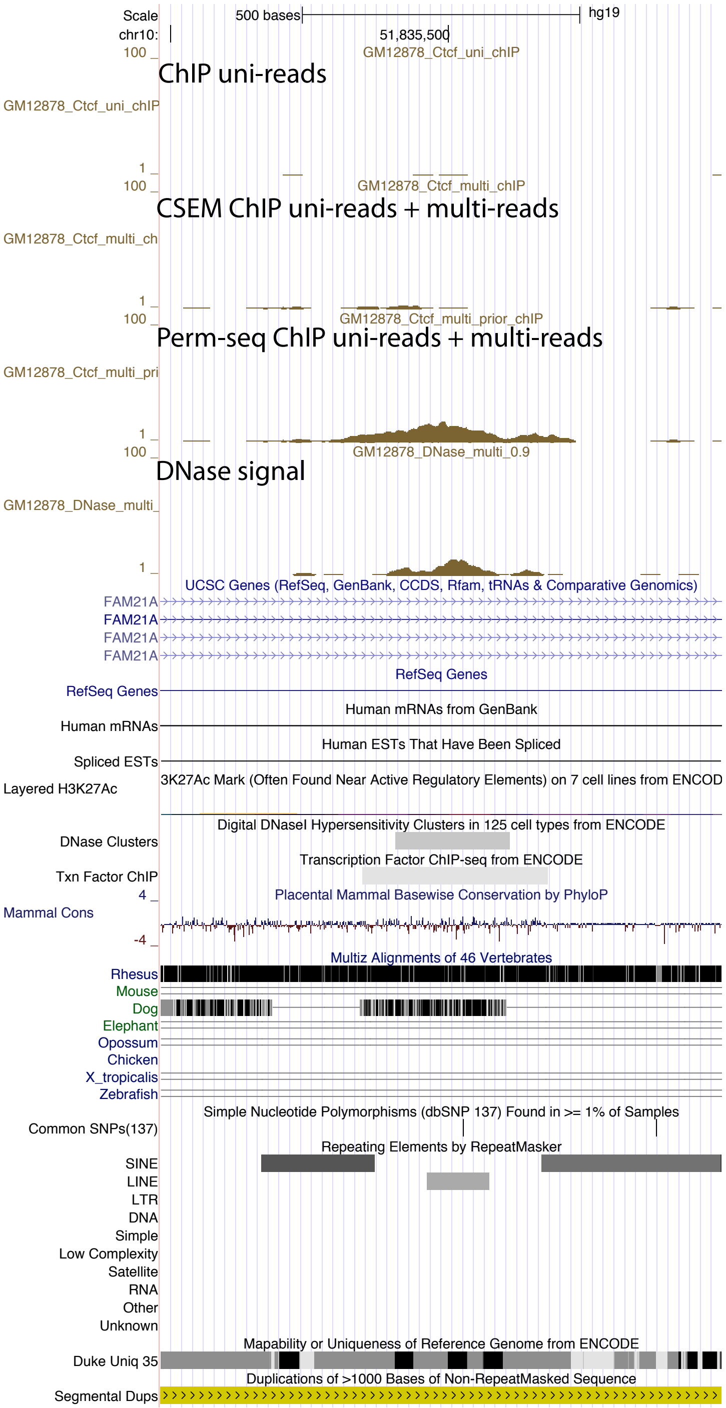

Supplementary Figure 6. UCSC Genome browser screen shot of a Perm-seq-exclusive Ctcf peak in GM12878 cells (chr10:51,835,319 -51,835,655) missed by uni-read and CSEM analysis but supported by DNase data.

3 Mappability in segmental duplication regions

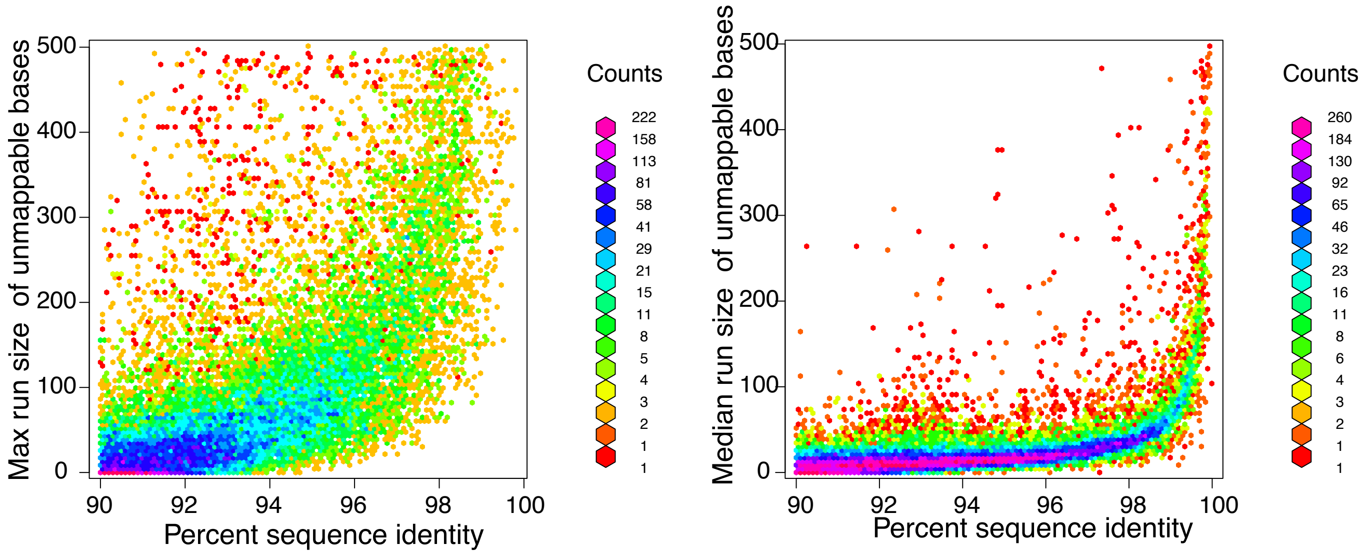

Supplementary Figure 7. (a) Maximum and (b) median run length of unmappable positions (with respect to 50 bps read length) across human segmental duplication regions. The first and third quartiles and the median values of maximum and median run lengths across all segmental duplication regions are (25, 163, 66) and (9, 26, 15), respectively.

4 Data-driven computational experiments

We compared Perm-seq, CSEM, a Gibbs-based approach [1], which is another multi-read allocation method similar to CSEM, and a random allocation approach that randomly allocates multi-reads to one of their mapping locations using data-driven computational experiments. We simulated read data using parameters estimated from the Atf3 ChIP-seq sample in GM12878 cells. To reduce computational time, we restricted our data generation process to chromosome 2. We used the actual DNase counts and the estimate of the read density π from the Perm-seq analysis of Atf3 sample to simulate ChIP read counts conditional on the DNase counts. Specifically, we considered three sequencing depth settings:

1. SE1 (low depth): 500,000 reads;
2. SE2 (average depth): 3,000,000 (approximately the number of chromosome 2 reads in the Atf3 sample);
3. SE3 (high depth): 5,000,000 reads.

Since uni-read allocation is the same for all the methods, we focused on the allocation accuracy of multi-reads. Furthermore, we also excluded reads for which the density at the true origin is not the maximum of the densities across all the mapping positions of the read. Although a portion of these reads can be allocated correctly if the value of the local read density around the true origin is high, they are likely to be wrongly allocated by all the methods in individual simulations.

Both the random allocation and Gibbs-based approaches assign multi-reads to a single position, therefore, we assigned multi-reads allocated by Perm-seq and CSEM to their mapping locations with the maximum allocation probabilities. Figure S8 displays the average proportion of correctly allocated reads at different sequencing depths across 10 simulation runs and indicates that Perm-seq performs better than other three approaches at all depths. On average, Perm-seq results in an average increase of 7.7% to 15.4% compared to CSEM and 9.5% and 34.5% compared to the Gibbs-based approach. The random allocation approach performs the worst in all settings, and, as expected, its performance does not improve with the increasing sequencing depth. CSEM outperforms Gibbs in the low depth setting with an increase of 6.7% in the percentage of correct allocations (with a standard error of 0.61%) and performs comparably to Gibbs-based in the higher depth settings. The allocation accuracies of Gibbs-based, CSEM, and Perm-seq increase when the sequencing depth is increased.

We next carried out a comparison of the allocation methods in terms of their impact on peak detection. We partitioned the genome into 200 bps non-overlapping intervals. We first computed interval-level read counts with only uni-reads and uni-reads and multi-reads using their true mapping locations. Then, we generated a set of gold standard enriched regions by identifying intervals with read counts larger than the 99-th percentile of the interval-level uni-read count distribution. Next, we allocated multi-reads by Gibbs-based, CSEM, and Perm-seq approaches and computed interval-level read counts for each method. We then evaluated these approaches in terms of sensitivity and positive predictive value (PPV) of multi-read peaks (peaks that are only identifiable using both multi- and uni-reads). Each method-specific enriched regions were identified following the procedure used for generating the gold standard enriched regions, i.e., peaks. Figure S8(b) illustrates that Perm-seq has higher sensitivity than both CSEM and Gibbs-based approach (4.6%-10% higher than CSEM and 3.4%-40.1% higher than the Gibbs-based approach with all standard errors less than 2%) for the three settings. Gibbs-based approach performs the worst for SE1 (low sequencing depth setting) with much lower sensitivity compared to Perm-seq and CSEM (40.1% lower than Perm-seq with standard error of 1.7% and 30.1% lower than CSEM with standard error of 2.2% for the three settings). The positive predictive value of Perm-seq is higher than the other two (3.7%-5.3% higher than CSEM with all standard errors less than 2% and 4.8%-17.6% higher than Gibbs-based with standard errors up to 2.8% for the three settings) and Gibbs-based is the lowest. Even though the multi-read allocation accuracy is comparable between CSEM and the Gibbs-based approach for higher sequencing depth settings, multi-reads wrongly allocated by the Gibbs-based approach result in more false positive enriched regions and a decrease in positive predictive value (10.4%, 12.5% lower than CSEM with standard errors less than 2% for SE2 and SE3). Overall, we observe that Perm-seq has consistently higher allocation accuracy compared to other approaches and the increase in the number of correctly allocated multi-reads can lead to detection of more enriched regions and elimination of falsely identified enrichments.

We next performed a more detailed comparison of Perm-seq and CSEM in terms of allocation accuracy and estimation accuracy of the read density π. We partitioned multi-reads into two groups: reads with maximum

allocation probability larger than 0.5 (group 1) and reads with maximum allocation probability smaller than or equal to 0.5 (group 2). Each method allocates reads in group 1 to their mapping positions with the maximum allocation probability. These group 1 reads are the subset of the multi-reads that are included in the peak calling pipeline. For reads in group 2, if the two largest allocation probabilities from CSEM or Perm-seq are the same, we label them as failed to be allocated by CSEM or Perm-seq. Remainder of the group 2 reads are allocated to the mapping positions with maximum allocation probability; however they are not utilized in the peak calling pipeline with the exception of the reads with allocation probability exactly equal to 0.5.

Figure S9 summarizes allocation accuracy by CSEM and Perm-seq across 10 simulation runs. Overall, these results illustrate that Perm-seq outperforms CSEM in these three sequencing depth settings. For the reads in the first group (reads that are utilized in peak calling), Perm-seq on average allocates 14.44% (with a standard error of 0.65%) more reads while keeping the same false positive rate as CSEM for the low sequencing depth setting. For the medium depth, while Perm-seq does not increase the allocation accuracy, it significantly reduces the number of false positives (8.41% with a standard error of 0.35%). For the high depth setting, Perm-seq shows an average of 8.6% (with a standard error of 0.23%) decrease in the false allocations at the cost of small decrease (2.4%) in the percentage of correct allocations. The general trend for the reads in the second group is that Perm-seq significantly decreases the percentage of reads that cannot be unambiguously allocated (30.44%, 6.1%, 1.03% with all standard errors less than 1% for the low, medium, and high depth settings).

To further investigate the impact of DNase-seq prior, we considered the multi-reads with only two mapping positions. We partitioned these reads into two groups according to the fold change of DNase-seq counts at the mapping locations. Specifically, we labeled the reads with log base 2 fold-changes of DNase-seq counts at the two mapping locations larger than or equal to 0.5 as the group that can be discriminated by DNase-seq and evaluated allocation accuracy of Perm-seq and CSEM for both groups (Figures S10(a), (b), (c)). If the DNase-seq does not have discriminating power, Perm-seq performs as good or better than CSEM (comparable increase in both the false and true positive allocations). In contrast, if DNase-seq has discriminating power, Perm-seq significantly increases the percentage of true positive allocations and decreases false allocations at all the three sequencing depth scenarios.

Finally, we evaluated the read density estimates from Perm-seq and CSEM by comparing their density estimates with the true value of the read density. Figure S10(d) illustrates that both of the Perm-seq and CSEM estimated densities have increasing correlation with the true density as a function of sequencing depth; however, Perm-seq consistently results in higher correlation than that of CSEM. A similar conclusion is observed when the total variation distance of the probability measures is used as a metric to compare the true and the estimated densities (data now shown).

(a)

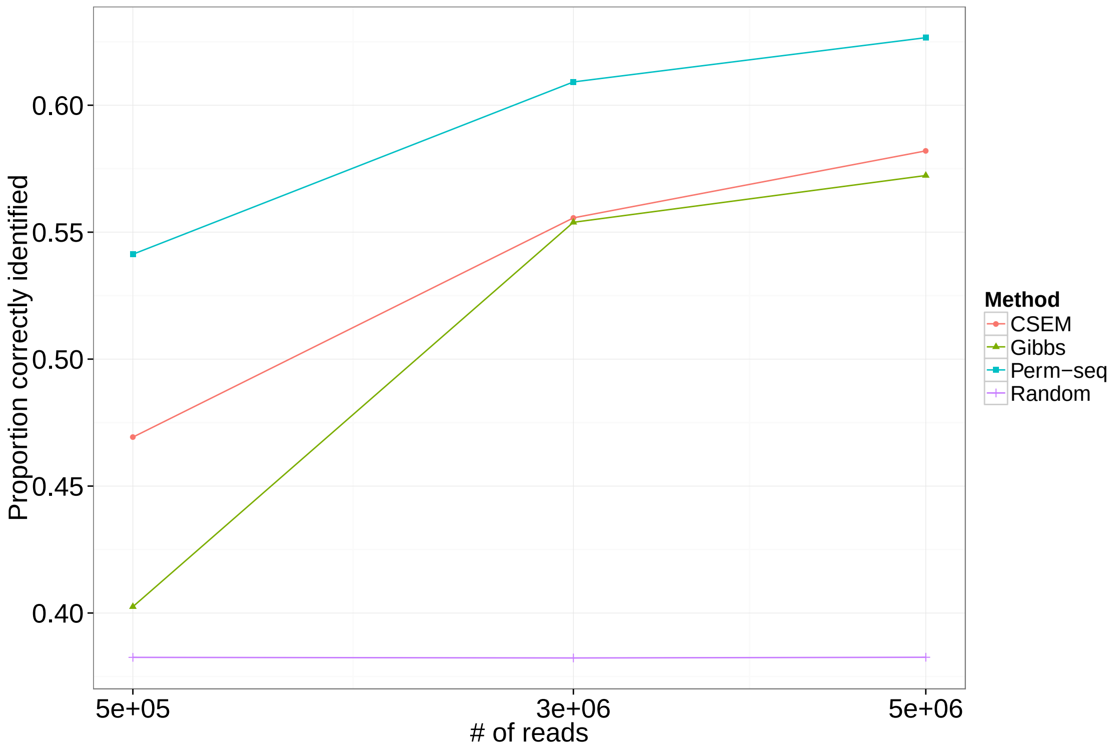

(b)

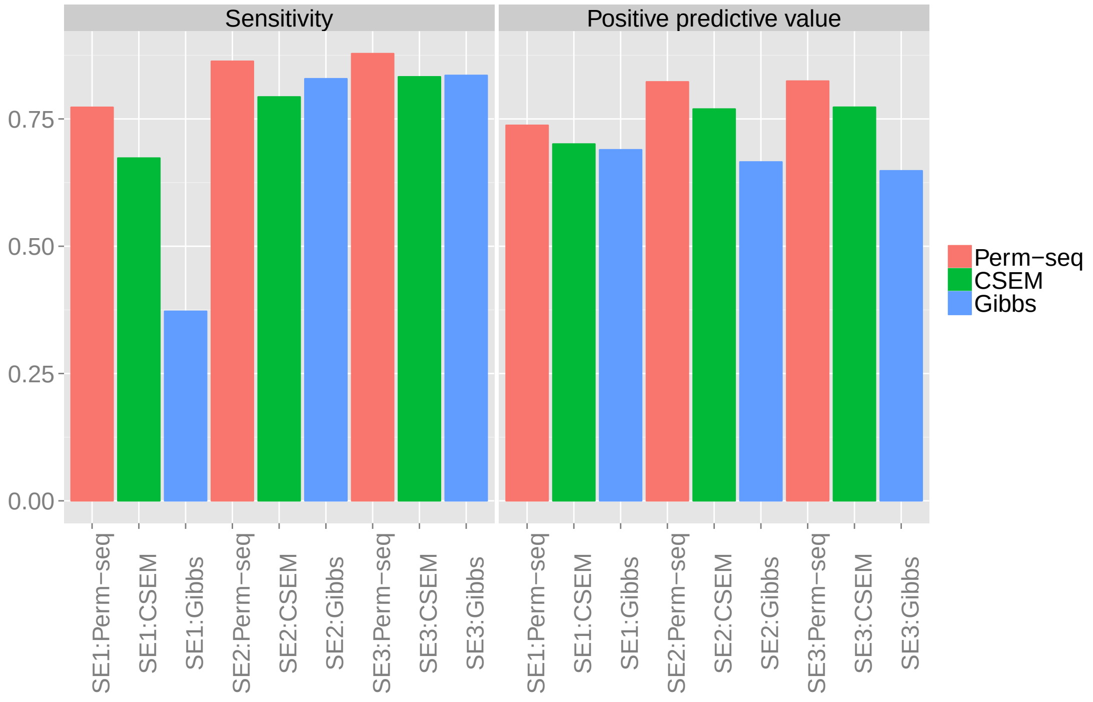

Supplementary Figure 8. Multi-read allocation comparisons between Perm-seq, CSEM, Gibbs-based [1] and the random allocation strategy. (a) Accuracy of multi-read allocation. For the Gibbs-based and random approaches, 1-(proportion correctly identified) represents the proportion of false positive allocations. For Perm-seq and CSEM, proportion of false allocations is smaller because the reads can be labeled as ambiguous based on their allocation probabilities as illustrated in Figure S12. (b) Evaluating different multi-read allocation approaches in terms of enrichment detection.

(a) Low depth

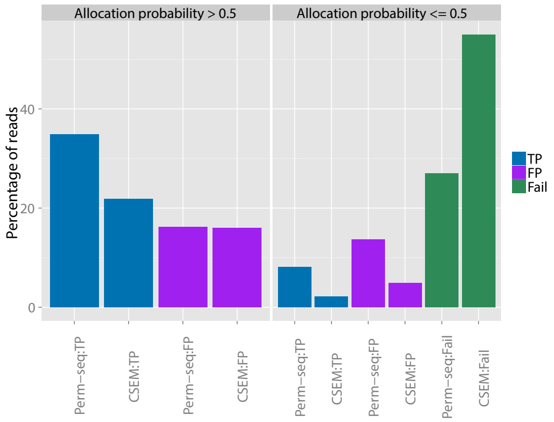

(b) Medium depth

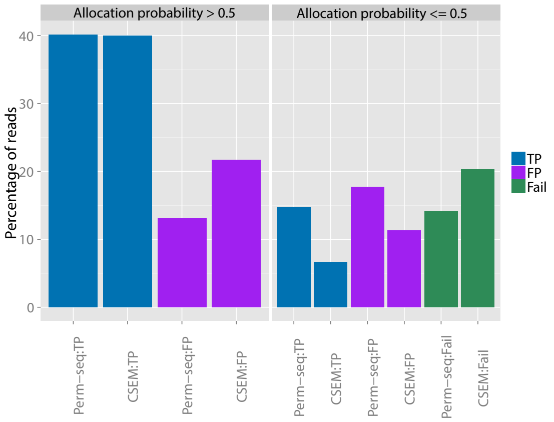

(c) High depth

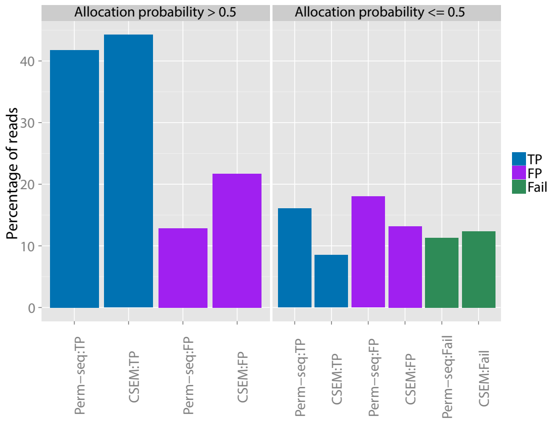

Supplementary Figure 9. Comparison of Perm-seq and CSEM multi-read allocation under varying sequencing depths. TP, FP, and Fail denote true positive, false positive, and ambiguous allocations, respectively. (a) SE1: Low depth. (b) SE2: Medium depthh. (c) SE3: High depth.

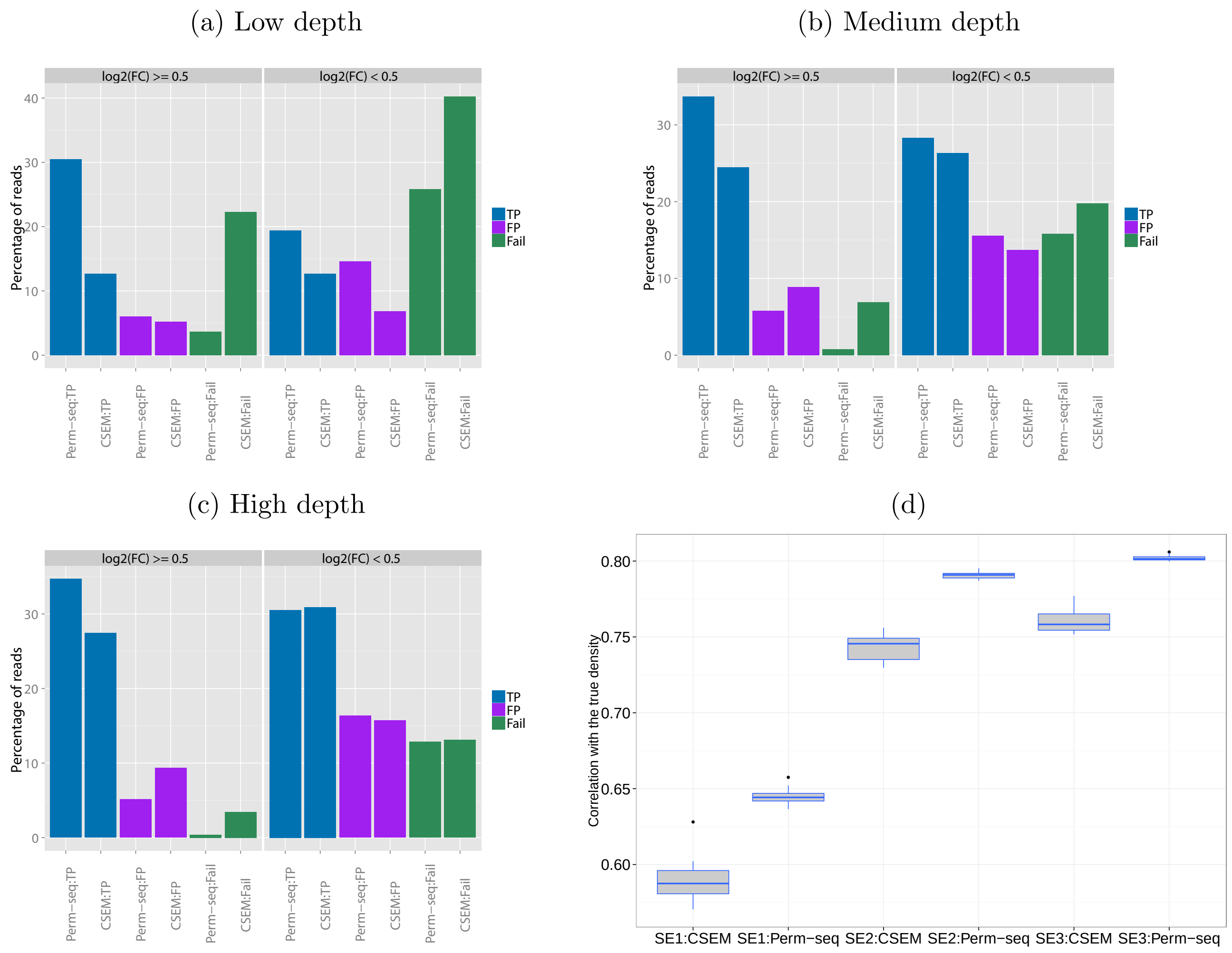

Supplementary Figure 10. (a, b, c) Comparison of Perm-seq and CSEM multi-read allocation for multi-reads with exactly two mapping positions. Fold-change (FC) is calculated between DNase-seq counts of the two mapping positions of each read. TP, FP, and Fail denote true positive, false positive, and ambiguous allocations, respectively. (d) Correlation of the Perm-seq and CSEM read density estimates with the true read density across 10 simulations.

5 Annotation of the peak lists

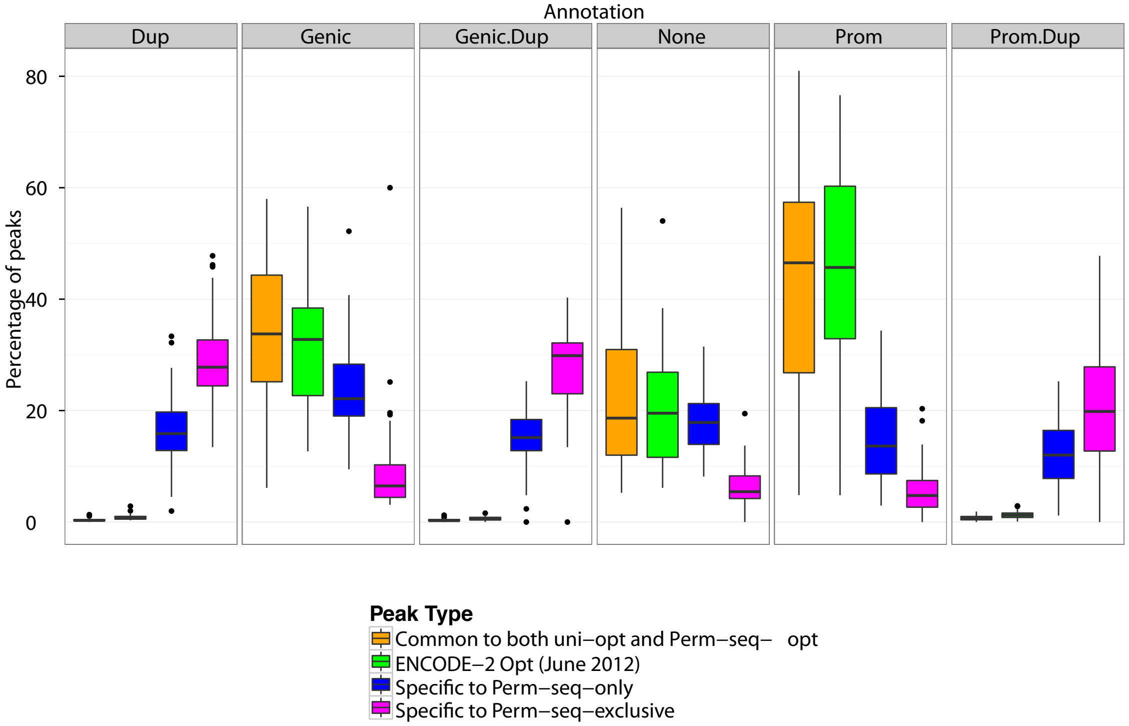

Supplementary Figure 11. Annotation of the GM12878 peaks with respect to segmental duplications. Categories are: Prom.Dup: peaks that are in promoter regions (± 2500 bps of TSS) of RefSeq genes that reside in segmental duplications; Prom: Peaks in promoter regions (excludes peaks in Prom.Dup); Genic.Dup: peaks that are within [-10000 bps of TSS, +1000 bps of TES] of RefSeq genes that are in segmental duplications (excludes peaks in Prom.Dup); Genic: peaks that are within [-10000 bps of TSS, +1000 bps of TES] of RefSeq genes (excludes peaks in Genic.Dup, Prom.Dup); Dup: peaks that are in segmental duplications (excludes Prom.Dup and Genic.Dup) ; None: peaks that do not fall into any of the other defined categories.

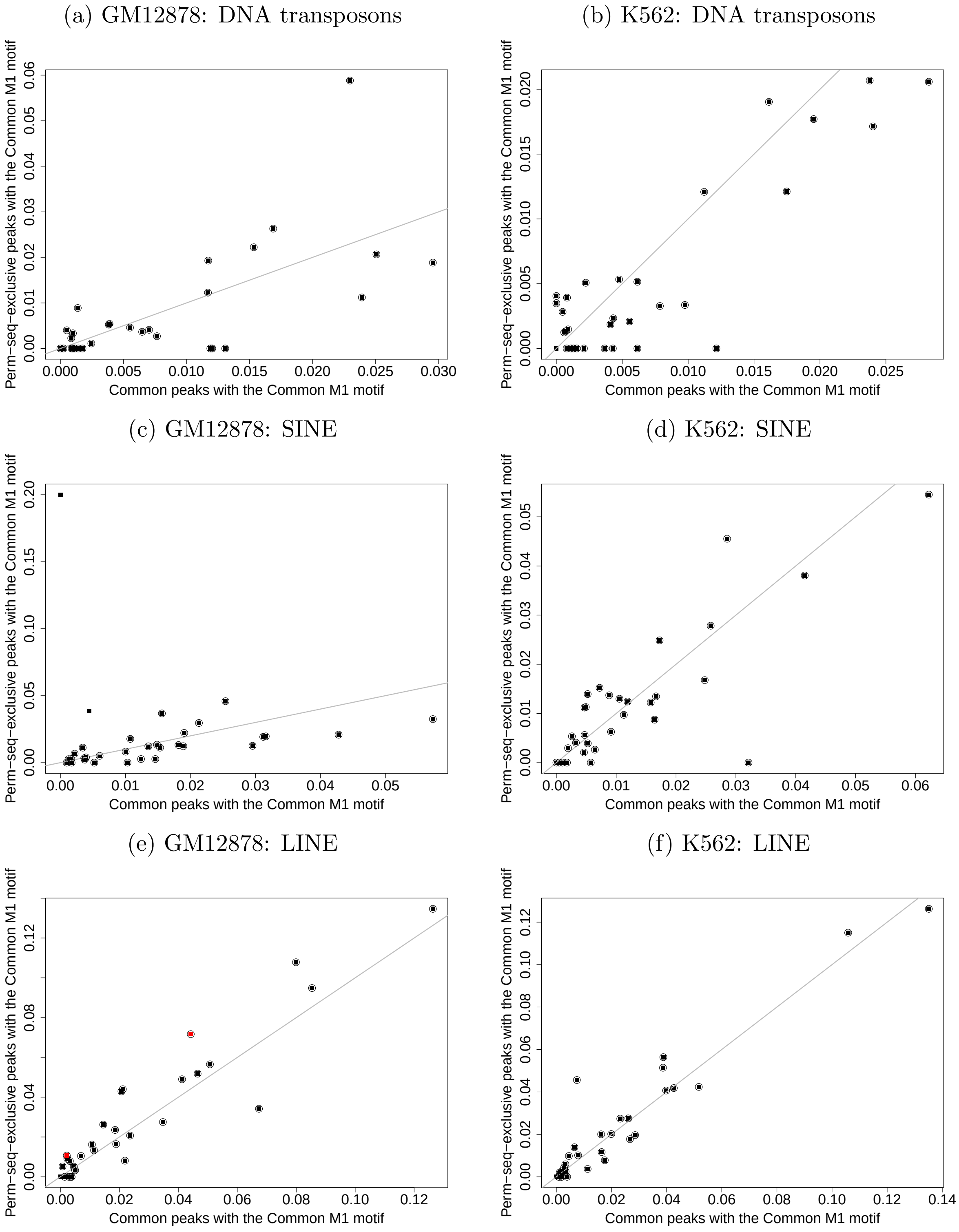

Supplementary Figure 12. Comparison of the proportions of the Common and Perm-seq-exclusive peaks with the canonical motif that overlap with DNA transposons, Short Interspersed Elements (SINE retrotransposons), and Long Interspersed Elements (LINE retrotransposons) repeat classes. TFs for which the repeat class enrichments are significantly different (Bonferoni adjusted p-value ≤ 0.05) between the Common and Perm-seq-exclusive sets are depicted in red. TFs for which the numbers of Common and Perm-seq-exclusive peaks with the Common M1 motif are larger than 50 are circled in black.

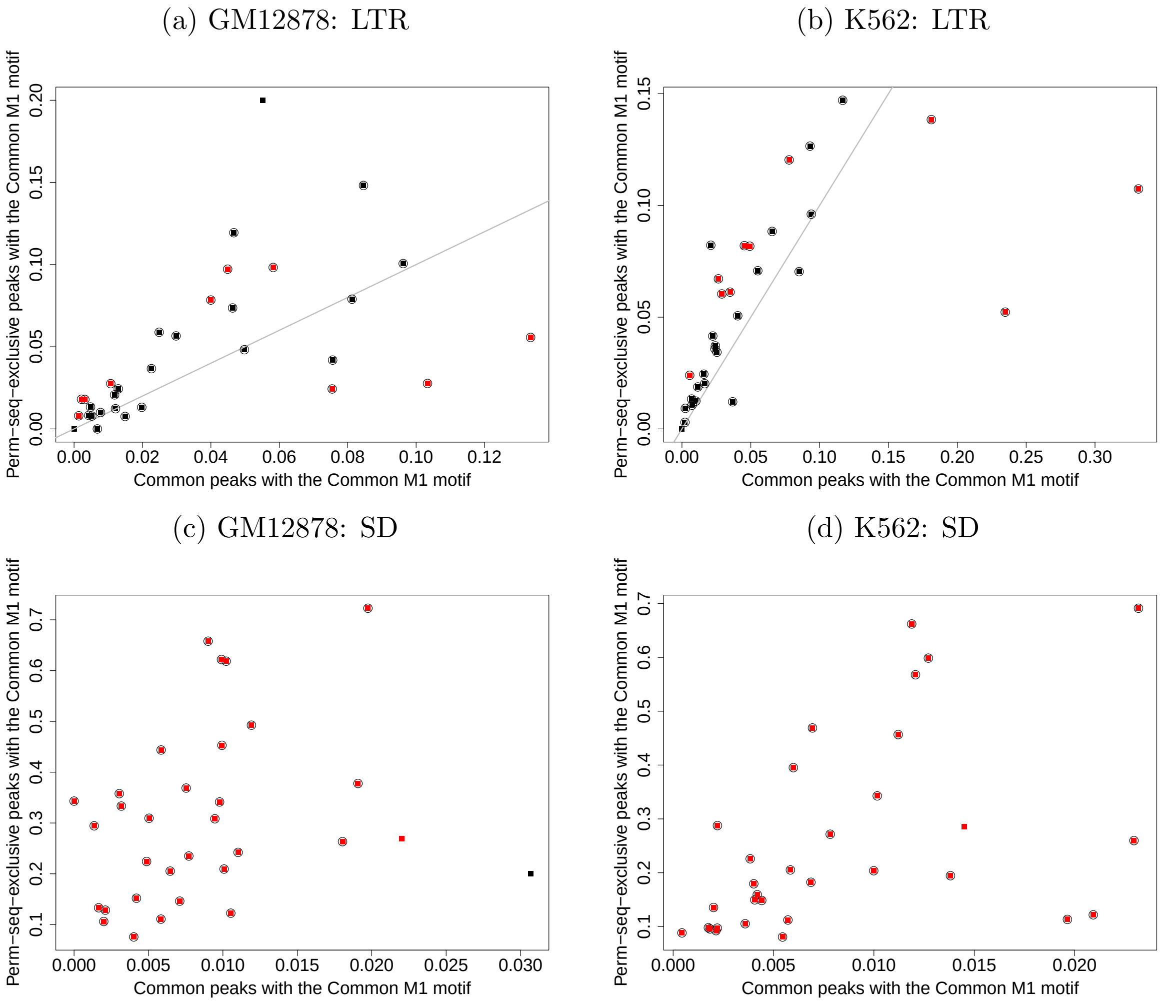

Supplementary Figure 13. Comparison of the proportions of the Common and Perm-seq-exclusive peaks with the canonical motif that overlap with Long Terminal Repeat (LTR retrotransposons) and Segmental Duplication repeat elements. TFs for which the repeat class enrichments are significantly different (Bonferoni adjusted p-value ≤ 0.05) between the Common and Perm-seq-exclusive sets are depicted in red. TFs for which the numbers of Common and Perm-seq-exclusive peaks with the Common M1 motif are larger than 50 are circled in black.

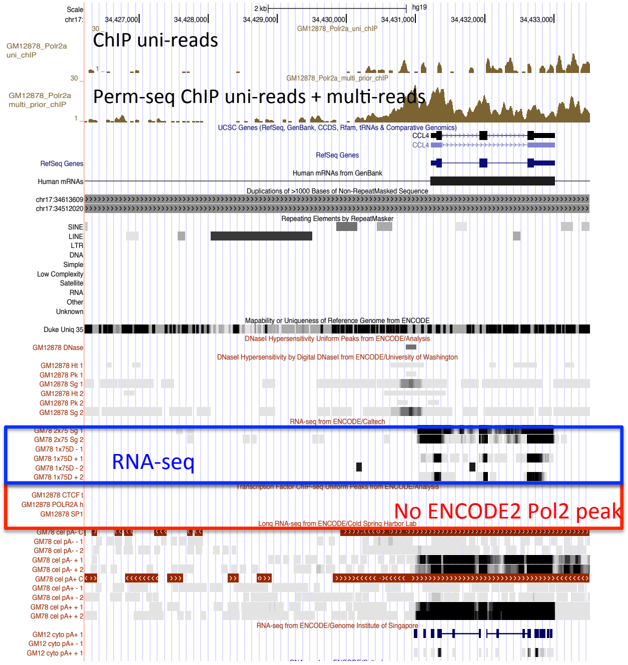

Supplementary Figure 14. UCSC Genome Browser screen shot of a Perm-seq-only Pol2 peak (GM12878) in the promoter region of the CCL4 gene expressed in GM12878 cells.

6 Sequence analysis of the peaks

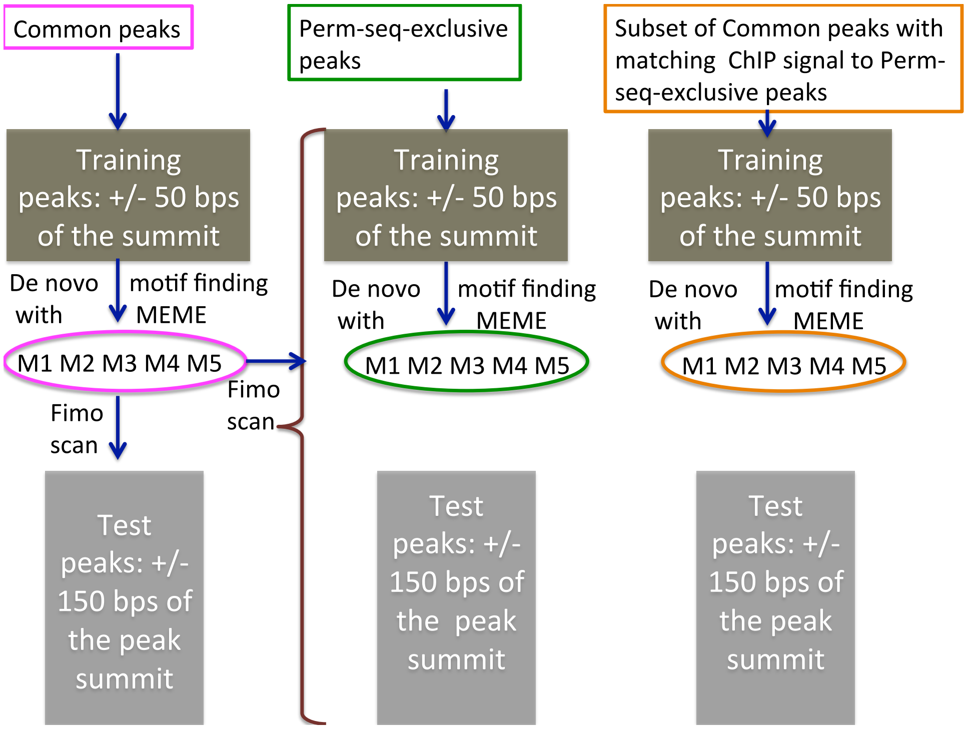

Supplementary Figure 15. Overall summary of the sequence analysis of the peak sets with the MEME Suite.

(a) GM12878

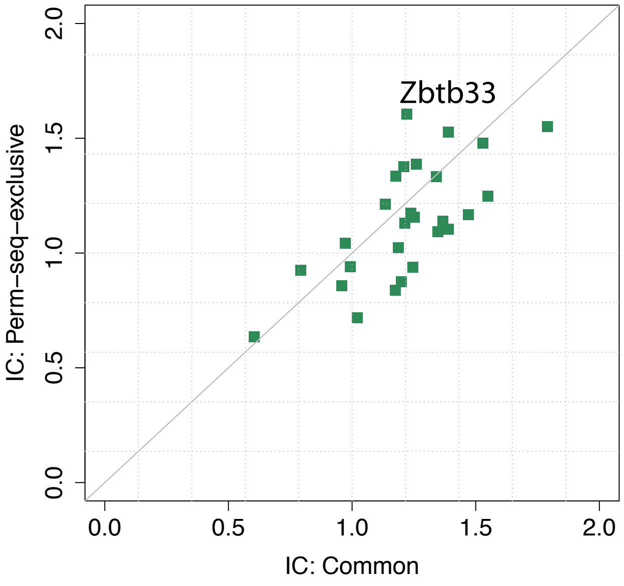

(b) K562

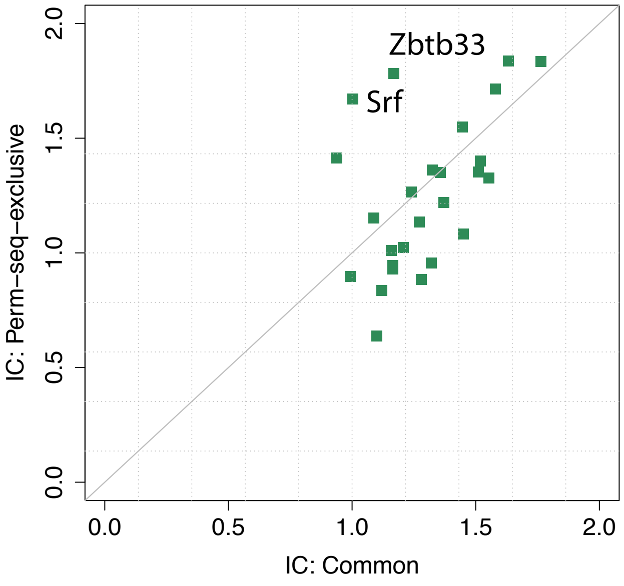

Supplementary Figure 16. Information contents of the canonical motifs learnt *de novo* from Perm-seq-exclusive and Common peak sets (green) in (a) GM12878 and (b) K562 cells. Orange squares compare Perm-seq-exclusive peaks with the subsets of the Common peaks with matching ChIP signal.

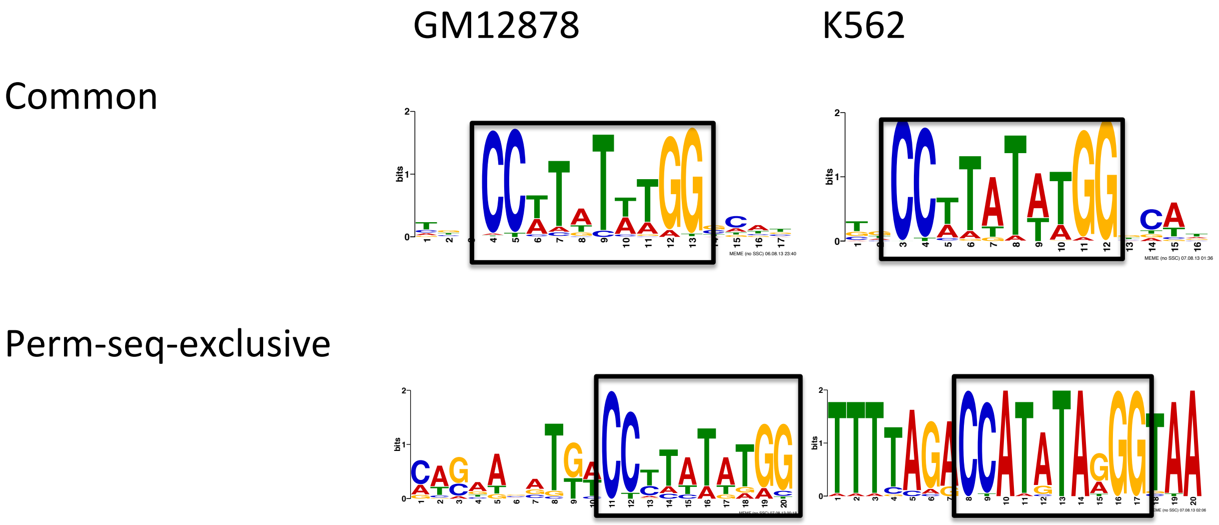

Supplementary Figure 17. Sequence logos of the Srf canonical motif identified from Common and Perm-seq-exclusive peak sets.

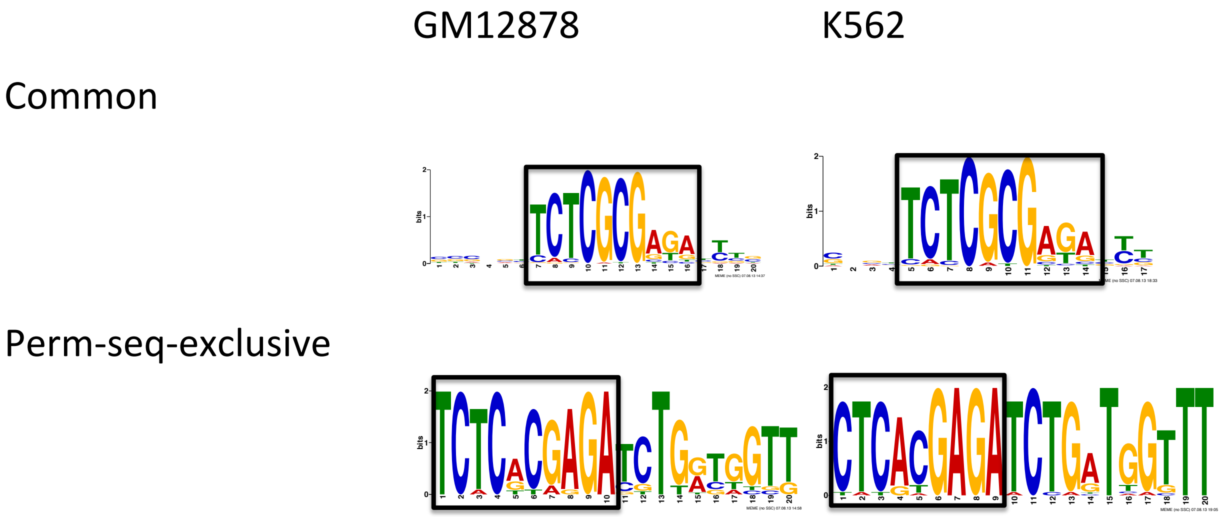

Supplementary Figure 18. Sequence logos of the Zbtb33 canonical motif identified from Common and Perm-seq-exclusive peak sets.

7 Perm-seq with multiple histone ChIP-seq datasets

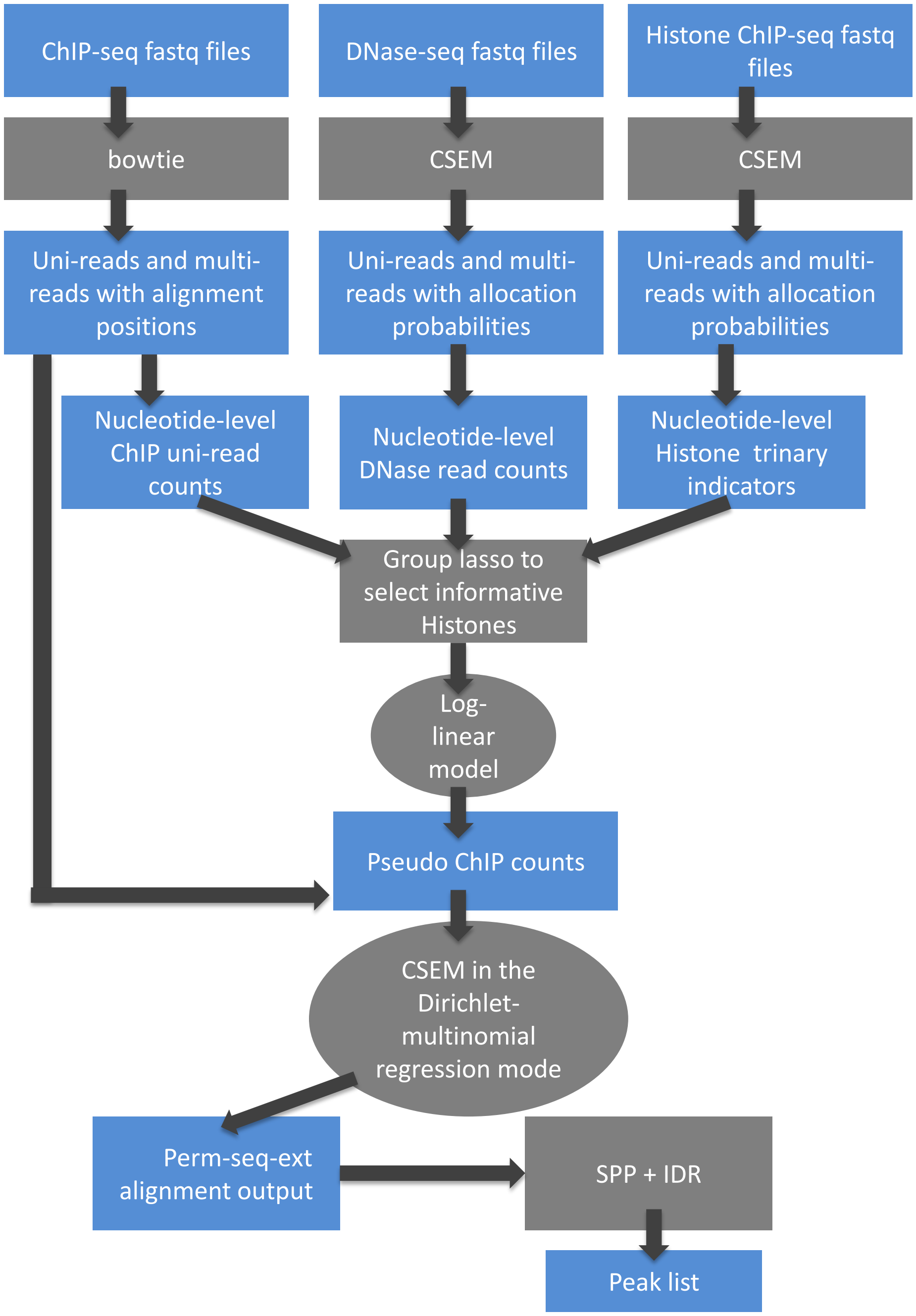

Supplementary Figure 19. Overall summary of the Perm-seq pipeline with both DNase-seq and Histone ChIP-seq for prior construction.

1

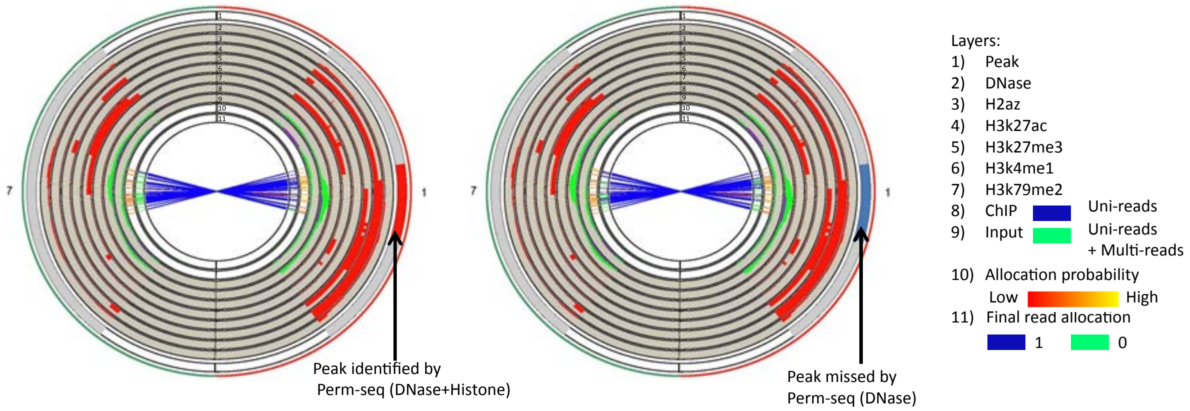

Supplementary Figure 20. Circos plots of Perm-seq (DNase+Histone) (left) and Perm-seq (DNase) (right) read allocation for reads mapping to two segmental duplication regions with coordinates chr1:83,647,856-83,955,427 and chr7:76,280,701-76,575,579.

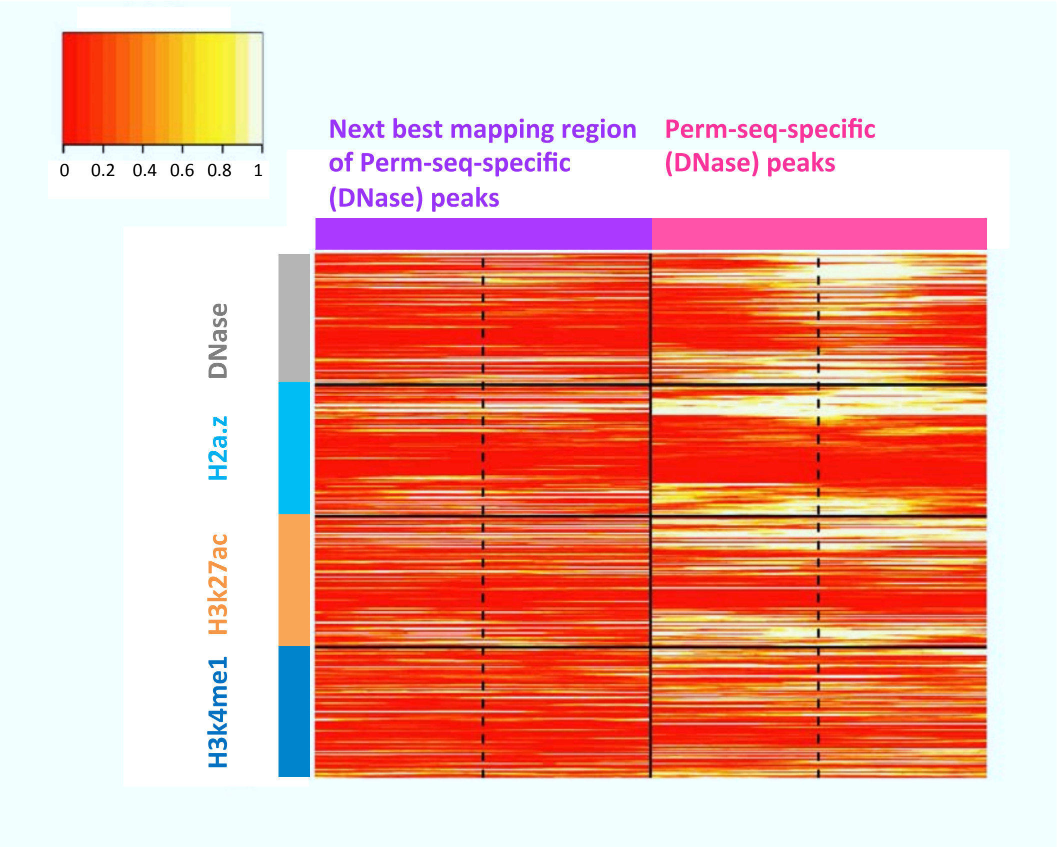

Supplementary Figure 21. Normalized DNase and Histone profiles of Perm-seq Egr1 peaks specific to DNase prior construction. "Perm-seq-specific (DNase) peaks" depict the set of peaks identified by Perm-seq when using DNase alone and are missed when Histone data is incorporated. "Next best mapping region of Perm-seq-specific (DNase) peaks" depicts the signal for the next best mapping region of every peak. Normalized DNase and histone read counts are plotted for the [-1000 bps, +1000 bps] window surrounding the region center marked by the dashed line.

(a)

(b)

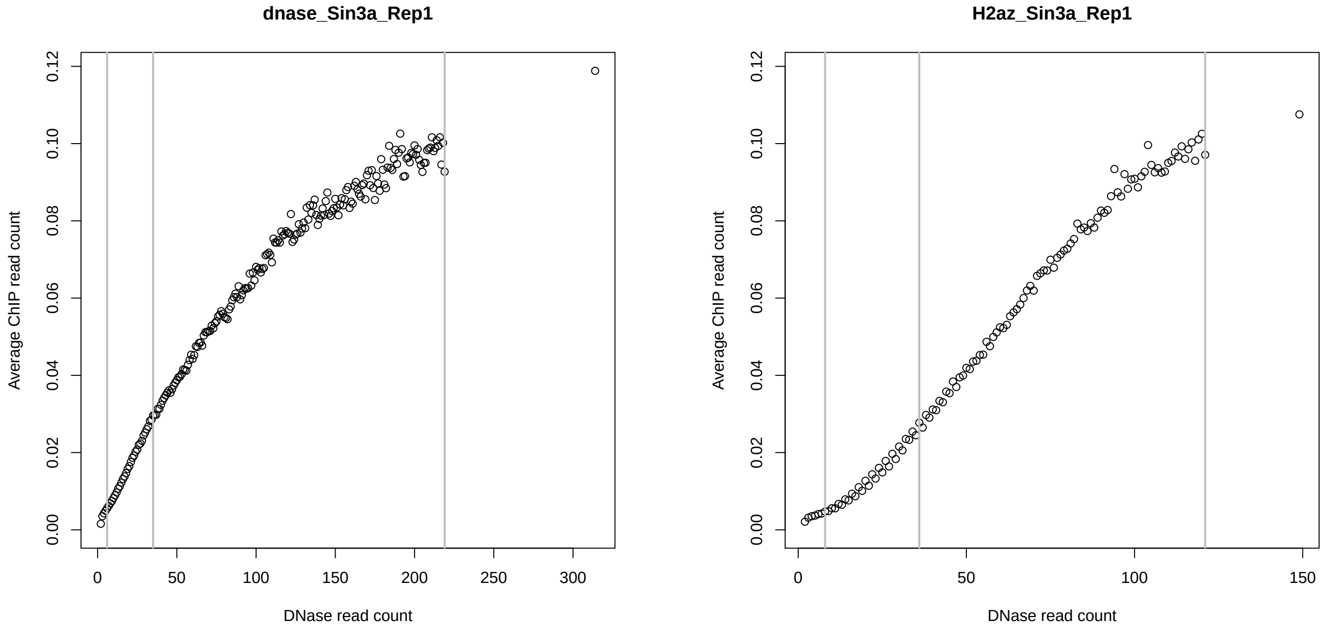

Supplementary Figure 22. ChIP versus DNase (a) and ChIP versus H2a.z read counts for replicate 1 of GM12878 Sin3a ChIP-seq dataset. Genomic positions with the same DNase (a) and H2a.z (b) read counts are grouped together and ChIP read counts are averaged within each group. Vertical lines indicate the knot points at the 90, 99, and 99.99-th percentiles of the DNase (a) and H2a.z (b) read count distributions.

8 Discriminative effect of DNase-seq as a function of sequencing depth

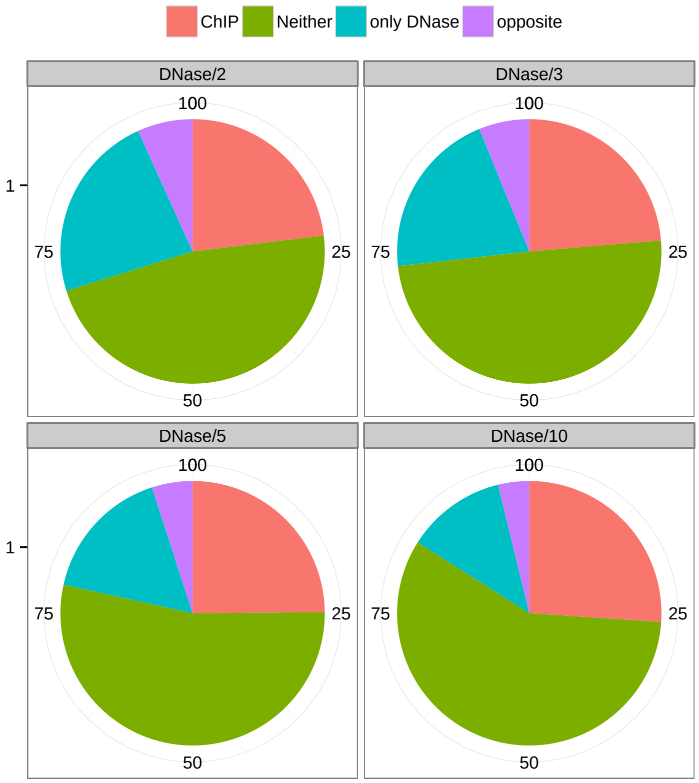

Supplementary Figure 23. Classification of multi-reads with two mapping locations based on their local DNase-seq and ChIP-seq read counts into 4 groups as ChIP, Neither, Only DNase, and Opposite in a Gata2 ChIP-seq experiment in Huvec cells as a function of the DNase-seq sample sequencing depth (at 1/2, 1/3, 1/5, and 1/10th of the original depth). Similar trends are observed for Atf3 and Fos ChIP-seq datasets (data not shown).

Threshold	Only DNase	Neither	Opposite	ChIP
0.2	32.4	38.3	9.5	19.8
0.5*	31.9	40.9	7.3	19.9
0.7	30.8	45.1	5.3	18.8
1	28.2	48.7	4.6	18.5
1.5	24.8	65.6	0.8	8.8
2	17.9	77.4	0.2	4.5

Supplementary Table 1. Discriminative power of DNase-seq for mapping locations of multi-reads at varying fold changes among the ChIP counts. The proportion of read pairs in the following categories are reported: ChIP: ChIP or both ChIP and DNase discriminates; Neither: Neither ChIP nor DNase discriminates; Only DNase: Only DNase discriminates; Opposite: ChIP and DNase log base 2 ratios have different signs.

	Sequencing Depth (Uni)	Sequencing Depth (Perm-seq)	Numbers of Peaks (Uni)	Numbers of Peaks (Perm-seq)	Numbers of Peaks (Perm-seq-only)	Numbers of Peaks (Perm-seq-exclusive)
Atf3	16516029	17886826	1371	1571	346	247
Bcl3	24804624	26580497	3091	2809	534	385
Bclaf1	26128225	27554942	4447	4920	1058	374
Chd2	33016355	35473599	7986	8479	1048	510
Ctcf	17899760	19222152	48254	49639	2032	826
Egr1	46860353	50053725	40164	41225	2705	1076
Elf1	31660321	34061486	32539	33402	2327	1126
Ep300	45690351	48940991	26312	27653	2327	915
Ets1	30286700	32477607	12685	13203	1504	804
Fos	9885554	11231813	6913	8200	1295	324
Gabpa	26109520	28019295	17879	19341	1733	888
Jund	8718546	9760083	941	1049	142	73
Max	43137887	46503236	56338	58592	4412	1748
Nfe2	7967333	8962819	2310	2912	607	113
Nr2c2	13655462	14823690	598	670	124	94
Nrf1	16340327	17540619	4177	4257	293	204
Polr2a	29049265	31608596	29172	30307	2978	1287
Polr3g	3787906	4312964	69	271	202	35
Rad21	22855732	24523813	41111	43363	2310	1064
Rest	35102371	37560381	22256	22814	1914	958
Sin3a	50987854	54527049	13515	13718	1440	679
Six5	26793781	28837703	1750	1866	337	287
Smc3	20104712	21659599	23839	24994	1517	817
Sp1	31192168	33299729	6965	7074	723	350
Spi1	27232079	29348841	25800	26934	2316	1156
Srf	29692557	32149981	6735	6929	842	540
Taf1	17601576	19034735	21869	22699	1560	708
Tbp	32530518	35528405	18344	19329	1766	759
Usf1	24735278	26643981	29307	31152	2769	1156
Usf2	29985854	32258094	2833	2839	378	298
Yy1	17266445	18703944	19266	20671	1844	789
Zbtb33	33812440	36336616	2598	3130	744	483

Supplementary Table 2. Sequencing depths and sizes of the peak sets for K562 datasets.

	Sequencing Depth (Uni)	Sequencing Depth (Perm-seq)	Numbers of Peaks (Uni)	Numbers of Peaks (Perm-seq)	Numbers of Peaks (Perm-seq-only)	Numbers of Peaks (Perm-seq-exclusive)
Atf3	21861261	23256837	4244	4536	370	191
Bcl3	27067187	29102517	9927	9887	1208	487
Bclaf1	45361560	48048569	22599	22033	1596	607
Chd2	31168753	33156846	14169	13524	840	383
Ctcf	22862934	24330241	41746	42617	1507	743
Egr1	36724620	39142985	18742	19473	1575	820
Elf1	34471115	36880333	28489	29204	1849	902
Ep300	19483999	20911226	3922	4142	513	218
Ets1	31936029	33854052	12312	12394	939	395
Fos	8051994	8953537	2113	2306	208	67
Gabpa	24040697	26433349	5292	5602	556	300
Jund	6488977	7180771	2098	2776	686	102
Max	9581947	10538259	2266	2849	626	163
Nfe2	25838663	27585032	186	174	11	5
Nr2c2	27545935	29504740	1071	1646	618	370
Nrf1	38860335	41279988	6100	6201	380	225
Polr2a	47515286	50918827	28619	29832	2265	762
Polr3g	5183255	5758061	238	268	42	26
Rad21	31292308	33257289	42646	43652	1764	823
Rest	24296581	26039299	4997	5620	719	380
Sin3a	16395785	19022096	14872	16061	2122	750
Six5	38786864	41243477	5745	6042	437	253
Smc3	50547607	53591628	30368	31215	1525	714
Sp1	27807875	29674738	30312	31233	1640	622
Spi1	37300139	39684445	45570	46223	2018	646
Srf	33221980	36311828	3087	3251	508	370
Taf1	31134954	33201103	15925	16195	987	446
Tbp	34142690	36331922	17206	17791	1256	443
Usf1	19840956	21964855	7958	8847	1026	467
Usf2	30273743	32245181	3827	6915	3089	159
Yy1	12994772	13996444	2095	2145	260	135
Zbtb33	25259596	27104717	2232	2482	366	212

Supplementary Table 3. Sequencing depths and sizes of the peak sets for GM12878 datasets.

	GM12878		K562	
Factor	CSEM	Perm-seq	CSEM	Perm-seq
Atf3	0.104	0.294	0.067	0.386
Bcl3	0.180	0.305	0.081	0.137
Bclaf1	0.116	0.141	0.069	0.170
Chd2	0.072	0.065	0.140	0.119
Ctcf	0.378	0.650	0.522	0.544
Egr1	0.353	0.640	0.575	0.656
Elf1	0.203	0.221	0.280	0.291
Ep300	0.343	0.398	0.143	0.148
Ets1	0.137	0.166	0.155	0.219
Fos	0.230	0.622	0.232	0.318
Gabpa	0.148	0.385	0.172	0.162
Jund	0.436	0.560	0.140	0.528
Max	0.206	0.418	0.219	0.184
Nfe2	0.800	1.000	0.397	0.683
Nr2c2	0.184	0.275	0.052	0.143
Nrf1	0.097	0.391	0.045	0.410
Polr2a	0.358	0.354	0.121	0.139
Polr3g	0.039	0.452	0.093	0.438
Rad21	0.404	0.418	0.509	0.497
Rest	0.201	0.292	0.171	0.184
Sin3a	0.289	0.386	0.107	0.150
Six5	0.074	0.131	0.099	0.337
Smc3	0.336	0.491	0.359	0.705
Sp1	0.409	0.459	0.074	0.144
Spi1	0.643	0.660	0.604	0.812
Srf	0.084	0.175	0.113	0.098
Taf1	0.149	0.222	0.165	0.153
Tbp	0.041	0.067	0.072	0.081
Usf1	0.211	0.414	0.344	0.418
Usf2	0.245	0.581	0.092	0.572
Yy1	0.249	0.484	0.235	0.200
Zbtb33	0.065	0.197	0.066	0.142

Supplementary Table 4. Proportion of Perm-seq specific and CSEM specific peaks with the most significant motifs identified from the *de novo* sequence analysis of the intersection peaks, i.e., peaks common to uni-read, CSEM, and Perm-seq analysis.

9 Computational experiment with a GM12878 Ctcf PE 101bps read dataset

Read length (SE)	# of reads	# of multireads	Random	CSEM	Perm-seq	Gibbs-based	Lonut
101	9317678	119492	0.34	0.54	0.53	0.47	0.44
75	9282172	244029	0.30	0.50	0.49	0.44	0.40
50	9120226	446091	0.27	0.48	0.48	0.43	0.37
36	8898129	626980	0.26	0.48	0.48	0.43	0.36

Supplementary Table 5. Proportion of correctly allocated multi-reads in different designs. The true origins of the reads are defined based on the uni-read alignments of the experiment with PE 101bps reads.

10 Data tables for Perm-seq with multiple histone ChIP-seq datasets

Histone	Atf3	Ctcf	Egr1	Ep300	Rest	Sin3a
H2a.z	1	1	1	1	1	1
H3k27ac	1	1	1	1	0	1
H3k27me3	0	1	1	1	1	1
H3k4me3	0	0	1	1	1	1
H3k36me3	1	0	0	1	1	1
H3k79me2	1	1	1	0	0	1
H3k4me1	1	1	1	0	0	0
H3k9me3	0	0	0	0	1	0
H3k9ac	0	0	0	0	0	0
H4k20me1	0	0	0	0	0	0
H3k4me2	0	0	0	0	0	0

Supplementary Table 6. Set of histone ChIP-seq data selected for prior construction by the Group Lasso approach. "1" indicates the inclusion of the histone data in the log-linear regression model.

TF	Perm-seq (DNase+Histone)	Perm-seq (DNase)	Prior-Common-only	Perm-seq-specific (DNase+Histone)	Perm-seq-specific (DNase)
Atf3	4516	4476	112	43	23
Ctcf	42848	42743	591	142	62
Rest	5586	5647	326	33	109
Sin3a	16135	16367	445	105	384
Egr1	18974	19185	494	50	260
Ep300	4163	4129	227	40	24

Supplementary Table 7. Comparison of peak lists from CSEM, Perm-seq (DNase), and Perm-seq (DNase+Histone). Perm-seq (DNase+Histone): Optimal peaks from Perm-seq with DNase and multiple histone data; Perm-seq (DNase): Optimal peaks from Perm-seq with only DNase data; Prior-Common-only: Peaks that are identified by both Perm-seq (DNase) and Perm-seq (DNase+Histone) but are not in the optimal peak list of CSEM; Perm-seq-specific (DNase+Histone): Perm-seq (DNase+Histone) optimal peaks not overlapping the Perm-seq (DNase) and CSEM optimal peaks; Perm-seq-specific (DNase): Perm-seq (DNase) optimal peaks not overlapping the Perm-seq (DNase+Histone) and CSEM optimal peaks.

TF	Perm-seq-DNase	Perm-seq-H2a.z	Prior-Common-only	Perm-seq-specific (DNase)	Perm-seq-specific (H2a.z)
Atf3	4476	4518	105	30	49
Ctcf	42743	42760	546	107	97
Rest	5647	5638	345	90	66
Sin3a	16367	15927	254	575	167
Egr1	19185	19154	593	161	130
Ep300	4129	4064	169	82	34

Supplementary Table 8. Comparison of peak sets from CSEM, Perm-seq (H2a.z), and Perm-seq (DNase). Perm-seq (DNase): Set of peaks identified by Perm-seq pipeline utilizing DNase data at IDR = 0.02; Perm-seq (H2az): Set of peaks identified by Perm-seq pipeline utilizing H2a.z data instead of DNase data at IDR = 0.02; Prior-Common-only: Peaks identified by both Perm-seq (DNase) and Perm-seq (H2a.z) but are not in the optimal peak list of CSEM; Perm-seq-specific (DNase): Perm-seq (DNase) optimal peaks not overlapping the Perm-seq peaks (H2a.z) and CSEM optimal peaks; Perm-seq-specific (H2a.z): Perm-seq (H2a.z) optimal peaks not overlapping the Perm-seq (DNase) peaks and CSEM optimal peaks.

11 Datasets used in the analysis

TF	ChIP samples	Input samples
Atf3	wgEncodeHaibTfbsGm12878Atf3Pcr1xRawDataRep1.fastq.gz, wgEncodeHaibTfbsGm12878Atf3Pcr1xRawDataRep2.fastq.gz	wgEncodeHaibTfbsGm12878RxlchPcr1xRawDataRep5.fastq.gz, wgEncodeHaibTfbsGm12878RxlchV0416101RawDataRep1.fastq.gz
Bcl3	wgEncodeHudsonalphaChipSeqRawDataRep1Gm12878Bcl3Pcr1xBcl3.fastq.gz, wgEncodeHudsonalphaChipSeqRawDataRep2Gm12878Bcl3Pcr1xBcl3.fastq.gz	wgEncodeHaibTfbsGm12878RxlchPcr1xRawDataRep1.fastq.gz, wgEncodeHaibTfbsGm12878RxlchPcr1xRawDataRep2.fastq.gz
Bclaf1	wgEncodeHaibTfbsGm12878Bclaf101388V0416101RawDataRep1.fastq.gz, wgEncodeHaibTfbsGm12878Bclaf101388V0416101RawDataRep2.fastq.gz	wgEncodeHaibTfbsGm12878RxlchPcr1xRawDataRep5.fastq.gz, wgEncodeHaibTfbsGm12878RxlchV0416101RawDataRep1.fastq.gz
Chd2	wgEncodeSydhTfbsGm12878Chd2ab68301IggmusRawDataRep1.fastq.gz, wgEncodeSydhTfbsGm12878Chd2ab68301IggmusRawDataRep2.fastq.gz	wgEncodeYaleChIPseqRawDataRep1Gm12878InputIggmus.fastq.gz, wgEncodeYaleChIPseqRawDataRep2Gm12878InputIggmus.fastq.gz
Ctcf	wgEncodeBroadHistoneGm12878CtcfStdRawDataRep1.fastq.gz, wgEncodeBroadHistoneGm12878CtcfStdRawDataRep2.fastq.gz	wgEncodeBroadHistoneGm12878ControlStdRawDataRep1.fastq.gz, wgEncodeBroadHistoneGm12878ControlStdRawDataRep2.fastq.gz
Egr1	wgEncodeHaibTfbsGm12878Egr1V0416101RawDataRep1.fastq.gz, wgEncodeHaibTfbsGm12878Egr1V0416101RawDataRep2.fastq.gz	wgEncodeHaibTfbsGm12878RxlchPcr2xRawDataRep1.fastq.gz, wgEncodeHaibTfbsGm12878RxlchPcr2xRawDataRep2.fastq.gz
Elf1	wgEncodeHaibTfbsGm12878Elf1sc631V0416101RawDataRep1.fastq.gz, wgEncodeHaibTfbsGm12878Elf1sc631V0416101RawDataRep2.fastq.gz	wgEncodeHaibTfbsGm12878RxlchPcr1xRawDataRep5.fastq.gz, wgEncodeHaibTfbsGm12878RxlchV0416101RawDataRep1.fastq.gz
Ep300	wgEncodeHaibTfbsGm12878P300RawDataRep1.fastq.gz, wgEncodeHaibTfbsGm12878P300RawDataRep2.fastq.gz	wgEncodeHaibTfbsGm12878RxlchPcr1xRawDataRep1.fastq.gz, wgEncodeHaibTfbsGm12878RxlchPcr1xRawDataRep2.fastq.gz
Ets1	wgEncodeHaibTfbsGm12878Ets1Pcr1xRawDataRep1.fastq.gz, wgEncodeHaibTfbsGm12878Ets1Pcr1xRawDataRep2.fastq.gz	wgEncodeHaibTfbsGm12878RxlchPcr1xRawDataRep5.fastq.gz, wgEncodeHaibTfbsGm12878RxlchV0416101RawDataRep1.fastq.gz
Fos	wgEncodeSydhTfbsGm12878CfosRawDataRep2.fastq.gz, wgEncodeSydhTfbsGm12878CfosRawDataRep3.fastq.gz	wgEncodeSydhTfbsGm12878InputRawData.fastq.gz
Gabpa	wgEncodeHaibTfbsGm12878GabpPcr2xRawDataRep1.fastq.gz, wgEncodeHaibTfbsGm12878GabpPcr2xRawDataRep2.fastq.gz	wgEncodeHaibTfbsGm12878RxlchPcr2xRawDataRep1.fastq.gz, wgEncodeHaibTfbsGm12878RxlchPcr2xRawDataRep2.fastq.gz
Jund	[illegible] wgEncodeSydhTfbsGm12878JundRawDataRep2.fastq.gz	wgEncodeSydhTfbsGm12878InputRawData.fastq.gz
Max	[illegible] wgEncodeSydhTfbsGm12878MaxRawDataRep2.fastq.gz	wgEncodeSydhTfbsGm12878InputRawData.fastq.gz
Nfe2	[illegible] wgEncodeSydhTfbsGm12878Nfe2sc22827StdRawDataRep2.fastq.gz	wgEncodeSydhTfbsGm12878InputRawData.fastq.gz
Nr2c2	[illegible] wgEncodeSydhTfbsGm12878Tr4RawDataRep2.fastq.gz	wgEncodeSydhTfbsGm12878InputRawData.fastq.gz
Nrf1	wgEncodeSydhTfbsGm12878Nrf1IggmusRawDataRep1.fastq.gz, wgEncodeSydhTfbsGm12878Nrf1IggmusRawDataRep2.fastq.gz	wgEncodeYaleChIPseqRawDataRep1Gm12878InputIggmus.fastq.gz, wgEncodeYaleChIPseqRawDataRep2Gm12878InputIggmus.fastq.gz
Polr2a	wgEncodeHaibTfbsGm12878Pol24h8Pcr1xRawDataRep1.fastq.gz, wgEncodeHaibTfbsGm12878Pol24h8Pcr1xRawDataRep2.fastq.gz	wgEncodeHaibTfbsGm12878RxlchPcr1xRawDataRep1.fastq.gz, wgEncodeHaibTfbsGm12878RxlchPcr1xRawDataRep2.fastq.gz
Polr3g	[illegible] wgEncodeSydhTfbsGm12878Pol3RawDataRep2.fastq.gz	wgEncodeSydhTfbsGm12878InputRawData.fastq.gz
Rad21	wgEncodeHaibTfbsGm12878Rad21V0416101RawDataRep1.fastq.gz, wgEncodeHaibTfbsGm12878Rad21V0416101RawDataRep2.fastq.gz	wgEncodeHaibTfbsGm12878RxlchPcr1xRawDataRep5.fastq.gz, wgEncodeHaibTfbsGm12878RxlchV0416101RawDataRep1.fastq.gz
Rest	wgEncodeHaibTfbsGm12878NrsfPcr2xRawDataRep1.fastq.gz, wgEncodeHaibTfbsGm12878NrsfPcr2xRawDataRep2.fastq.gz	wgEncodeHaibTfbsGm12878RxlchPcr2xRawDataRep1.fastq.gz, wgEncodeHaibTfbsGm12878RxlchPcr2xRawDataRep2.fastq.gz
Sin3a	wgEncodeHudsonalphaChipSeqRawDataRep1Gm12878Sin3ak20Pcr2x.fastq.gz, wgEncodeHudsonalphaChipSeqRawDataRep2Gm12878Sin3ak20Pcr2x.fastq.gz	wgEncodeHaibTfbsGm12878RxlchPcr1xRawDataRep1.fastq.gz, wgEncodeHaibTfbsGm12878RxlchPcr1xRawDataRep2.fastq.gz
Six5	wgEncodeHaibTfbsGm12878Six5Pcr1xRawDataRep1.fastq.gz, wgEncodeHaibTfbsGm12878Six5Pcr1xRawDataRep2.fastq.gz	wgEncodeHaibTfbsGm12878RxlchPcr1xRawDataRep4.fastq.gz, wgEncodeHaibTfbsGm12878RxlchPcr1xRawDataRep5.fastq.gz
Smc3	wgEncodeSydhTfbsGm12878Smc3ab9263IggmusRawDataRep1.fastq.gz, wgEncodeSydhTfbsGm12878Smc3ab9263IggmusRawDataRep2.fastq.gz	wgEncodeYaleChIPseqRawDataRep1Gm12878InputIggmus.fastq.gz, wgEncodeYaleChIPseqRawDataRep2Gm12878InputIggmus.fastq.gz
Sp1	wgEncodeHaibTfbsGm12878Sp1Pcr1xRawDataRep1.fastq.gz, wgEncodeHaibTfbsGm12878Sp1Pcr1xRawDataRep2.fastq.gz	wgEncodeHaibTfbsGm12878RxlchPcr1xRawDataRep2.fastq.gz, wgEncodeHaibTfbsGm12878RxlchPcr1xRawDataRep3.fastq.gz
Spi1	wgEncodeHaibTfbsGm12878Pu1Pcr1xRawDataRep1.fastq.gz, wgEncodeHaibTfbsGm12878Pu1Pcr1xRawDataRep2.fastq.gz	wgEncodeHaibTfbsGm12878RxlchPcr1xRawDataRep1.fastq.gz, wgEncodeHaibTfbsGm12878RxlchPcr1xRawDataRep2.fastq.gz
Srf	wgEncodeHaibTfbsGm12878SrfPcr2xRawDataRep1.fastq.gz, wgEncodeHaibTfbsGm12878SrfPcr2xRawDataRep2.fastq.gz	wgEncodeHaibTfbsGm12878RxlchPcr2xRawDataRep1.fastq.gz, wgEncodeHaibTfbsGm12878RxlchPcr2xRawDataRep2.fastq.gz
Taf1	wgEncodeHaibTfbsGm12878Taf1Pcr1xRawDataRep1.fastq.gz, wgEncodeHaibTfbsGm12878Taf1Pcr1xRawDataRep2.fastq.gz	wgEncodeHaibTfbsGm12878RxlchPcr1xRawDataRep1.fastq.gz, wgEncodeHaibTfbsGm12878RxlchPcr1xRawDataRep2.fastq.gz
Tbp	wgEncodeSydhTfbsGm12878TbpIggmusRawDataRep1.fastq.gz, wgEncodeSydhTfbsGm12878TbpIggmusRawDataRep2.fastq.gz	wgEncodeYaleChIPseqRawDataRep1Gm12878InputIggmus.fastq.gz, wgEncodeYaleChIPseqRawDataRep2Gm12878InputIggmus.fastq.gz
Usf1	wgEncodeHaibTfbsGm12878Usf1Pcr2xRawDataRep1.fastq.gz, wgEncodeHaibTfbsGm12878Usf1Pcr2xRawDataRep2.fastq.gz	wgEncodeHaibTfbsGm12878RxlchPcr2xRawDataRep1.fastq.gz, wgEncodeHaibTfbsGm12878RxlchPcr2xRawDataRep2.fastq.gz
Usf2	wgEncodeSydhTfbsGm12878Usf2IggmusRawDataRep1.fastq.gz, wgEncodeSydhTfbsGm12878Usf2IggmusRawDataRep2.fastq.gz	wgEncodeYaleChIPseqRawDataRep1Gm12878InputIggmus.fastq.gz, wgEncodeYaleChIPseqRawDataRep2Gm12878InputIggmus.fastq.gz
Yy1	[illegible] wgEncodeSydhTfbsGm12878Yy1RawDataRep2.fastq.gz	wgEncodeSydhTfbsGm12878InputRawData.fastq.gz
Zbtb33	wgEncodeHaibTfbsGm12878Zbtb33RawDataRep1.fastq.gz, wgEncodeHaibTfbsGm12878Zbtb33RawDataRep2.fastq.gz	wgEncodeHaibTfbsGm12878RxlchPcr1xRawDataRep1.fastq.gz, wgEncodeHaibTfbsGm12878RxlchPcr1xRawDataRep2.fastq.gz

Supplementary Table 9. ChIP and Input fastq files for GM12878.

TF	ChIP samples	Input samples
Atf3	wgEncodeSydhTfbsK562Atf3RawDataRep1.fastq.gz, wgEncodeSydhTfbsK562Atf3RawDataRep2.fastq.gz	SydhK562InputStdAlnRepRep1.fastq.gz, SydhK562InputStdAlnRepRep2.fastq.gz
Bcl3	wgEncodeHaibTfbsK562Bcl3Pcr1xRawDataRep1.fastq.gz, wgEncodeHaibTfbsK562Bcl3Pcr1xRawDataRep2.fastq.gz	wgEncodeHaibTfbsK562RxlchV0416101RawDataRep1.fastq.gz, wgEncodeHaibTfbsK562RxlchV0416101RawDataRep2.fastq.gz
Bclaf1	wgEncodeHaibTfbsK562Bclaf101388Pcr1xRawDataRep1.fastq.gz, wgEncodeHaibTfbsK562Bclaf101388Pcr1xRawDataRep2.fastq.gz	wgEncodeHaibTfbsK562RxlchV0416101RawDataRep1.fastq.gz, wgEncodeHaibTfbsK562RxlchV0416101RawDataRep2.fastq.gz
Chd2	wgEncodeSydhTfbsK562Chd2ab68301IggrabRawDataRep1.fastq.gz, wgEncodeSydhTfbsK562Chd2ab68301IggrabRawDataRep2.fastq.gz	SydhK562InputIggrabAlnRep1.fastq.gz
Ctcf	wgEncodeBroadHistoneK562CtcfStdRawDataRep1.fastq.gz, wgEncodeBroadHistoneK562CtcfStdRawDataRep2.fastq.gz	wgEncodeBroadHistoneK562ControlStdRawDataRep1.fastq.gz
Egr1	wgEncodeHaibTfbsK562Egr1V0416101RawDataRep1.fastq.gz, wgEncodeHaibTfbsK562Egr1V0416101RawDataRep2.fastq.gz	wgEncodeHaibTfbsK562RxlchV0416101RawDataRep1.fastq.gz, wgEncodeHaibTfbsK562RxlchV0416101RawDataRep2.fastq.gz
Elf1	wgEncodeHaibTfbsK562Elf1sc631V0416102RawDataRep1.fastq.gz, wgEncodeHaibTfbsK562Elf1sc631V0416102RawDataRep2.fastq.gz	wgEncodeHaibTfbsK562RxlchPcr1xRawDataRep3.fastq.gz, wgEncodeHaibTfbsK562RxlchPcr1xRawDataRep4.fastq.gz
Ep300	wgEncodeSydhTfbsK562P300f4IggrabAlnRep1.fastq.gz, wgEncodeSydhTfbsK562P300f4IggrabAlnRep2.fastq.gz	SydhK562InputIggrabAlnRep1.fastq.gz
Ets1	wgEncodeHaibTfbsK562Ets1V0416101RawDataRep1.fastq.gz, wgEncodeHaibTfbsK562Ets1V0416101RawDataRep2.fastq.gz	wgEncodeHaibTfbsK562RxlchV0416101RawDataRep1.fastq.gz, wgEncodeHaibTfbsK562RxlchV0416101RawDataRep2.fastq.gz
Fos	wgEncodeSydhTfbsK562CfosRawDataRep1.fastq.gz, wgEncodeSydhTfbsK562CfosRawDataRep2.fastq.gz, wgEncodeSydhTfbsK562CfosRawDataRep3.fastq.gz	SydhK562InputStdAlnRepRep1.fastq.gz, SydhK562InputStdAlnRepRep2.fastq.gz
Gabpa	wgEncodeHaibTfbsK562GabpV0416101RawDataRep1.fastq.gz, wgEncodeHaibTfbsK562GabpV0416101RawDataRep2.fastq.gz	wgEncodeHaibTfbsK562RxlchV0416101RawDataRep1.fastq.gz, wgEncodeHaibTfbsK562RxlchV0416101RawDataRep2.fastq.gz
Jund	wgEncodeYaleChIPseqRawDataRep1K562Jund.fastq.gz, wgEncodeYaleChIPseqRawDataRep2K562Jund.fastq.gz	SydhK562InputStdAlnRepRep1.fastq.gz, SydhK562InputStdAlnRepRep2.fastq.gz
Max	wgEncodeHaibTfbsK562MaxV0416102RawDataRep1.fastq.gz, wgEncodeHaibTfbsK562MaxV0416102RawDataRep2.fastq.gz	wgEncodeHaibTfbsK562RxlchPcr1xRawDataRep3.fastq.gz, wgEncodeHaibTfbsK562RxlchPcr1xRawDataRep4.fastq.gz
Nfe2	wgEncodeSydhTfbsK562Nfe2RawDataRep1.fastq.gz, wgEncodeSydhTfbsK562Nfe2RawDataRep2.fastq.gz	SydhK562InputStdAlnRepRep1.fastq.gz, SydhK562InputStdAlnRepRep2.fastq.gz
Nr2c2	wgEncodeSydhTfbsK562Tr4RawDataRep1.fastq.gz, wgEncodeSydhTfbsK562Tr4RawDataRep2.fastq.gz	SydhK562bInputUcdAlnRep1.fastq.gz
Nrf1	wgEncodeSydhTfbsK562Nrf1IggrabRawDataRep1.fastq.gz, wgEncodeSydhTfbsK562Nrf1IggrabRawDataRep2.fastq.gz	SydhK562InputIggrabAlnRep1.fastq.gz
Polr2a	wgEncodeBroadHistoneK562Pol2bStdRawDataRep1.fastq.gz, wgEncodeBroadHistoneK562Pol2bStdRawDataRep2.fastq.gz	wgEncodeBroadHistoneK562ControlStdRawDataRep1.fastq.gz
Polr3g	wgEncodeSydhTfbsK562Pol3RawDataRep1.fastq.gz, wgEncodeSydhTfbsK562Pol3RawDataRep2.fastq.gz	SydhK562InputStdAlnRepRep1.fastq.gz, SydhK562InputStdAlnRepRep2.fastq.gz
Rad21	wgEncodeHaibTfbsK562Rad21V0416102RawDataRep1.fastq.gz, wgEncodeHaibTfbsK562Rad21V0416102RawDataRep2.fastq.gz	wgEncodeHaibTfbsK562RxlchPcr1xRawDataRep3.fastq.gz, wgEncodeHaibTfbsK562RxlchPcr1xRawDataRep4.fastq.gz
Rest	wgEncodeHaibTfbsK562NrsfV0416102RawDataRep1.fastq.gz, wgEncodeHaibTfbsK562NrsfV0416102RawDataRep2.fastq.gz	wgEncodeHaibTfbsK562RxlchV0416101RawDataRep1.fastq.gz, wgEncodeHaibTfbsK562RxlchV0416101RawDataRep2.fastq.gz
Sin3a	wgEncodeHaibTfbsK562Sin3ak20V0416101RawDataRep1.fastq.gz, wgEncodeHaibTfbsK562Sin3ak20V0416101RawDataRep2.fastq.gz	wgEncodeHaibTfbsK562RxlchV0416101RawDataRep1.fastq.gz, wgEncodeHaibTfbsK562RxlchV0416101RawDataRep2.fastq.gz
Six5	wgEncodeHaibTfbsK562Six5RawDataRep1.fastq.gz, wgEncodeHaibTfbsK562Six5RawDataRep2.fastq.gz	wgEncodeHaibTfbsK562RxlchV2RawDataRep1.fastq.gz, wgEncodeHaibTfbsK562RxlchV2RawDataRep2.fastq.gz
Smc3	wgEncodeSydhTfbsK562Smc3ab9263IggrabRawDataRep1.fastq.gz, wgEncodeSydhTfbsK562Smc3ab9263IggrabRawDataRep2.fastq.gz	SydhK562InputIggrabAlnRep1.fastq.gz
Sp1	wgEncodeHaibTfbsK562Sp1Pcr1xRawDataRep1.fastq.gz, wgEncodeHaibTfbsK562Sp1Pcr1xRawDataRep2.fastq.gz	wgEncodeHaibTfbsK562RxlchV0416101RawDataRep1.fastq.gz, wgEncodeHaibTfbsK562RxlchV0416101RawDataRep2.fastq.gz
Spi1	wgEncodeHaibTfbsK562Pu1Pcr1xRawDataRep1.fastq.gz, wgEncodeHaibTfbsK562Pu1RawDataRep2.fastq.gz	wgEncodeHaibTfbsK562RxlchV2RawDataRep1.fastq.gz, wgEncodeHaibTfbsK562RxlchV2RawDataRep2.fastq.gz
Srf	wgEncodeHaibTfbsK562SrfV0416101RawDataRep1.fastq.gz, wgEncodeHaibTfbsK562SrfV0416101RawDataRep2.fastq.gz	wgEncodeHaibTfbsK562RxlchV0416101RawDataRep1.fastq.gz, wgEncodeHaibTfbsK562RxlchV0416101RawDataRep2.fastq.gz
Taf1	wgEncodeHaibTfbsK562Taf1V0416101RawDataRep1.fastq.gz, wgEncodeHaibTfbsK562Taf1V0416101RawDataRep2.fastq.gz	wgEncodeHaibTfbsK562RxlchV0416101RawDataRep1.fastq.gz, wgEncodeHaibTfbsK562RxlchV0416101RawDataRep2.fastq.gz
Tbp	wgEncodeSydhTfbsK562TbpIggmusRawDataRep1.fastq.gz, wgEncodeSydhTfbsK562TbpIggmusRawDataRep2.fastq.gz	SydhK562InputIggmusAlnRep1.fastq.gz, SydhK562InputIggmusAlnRep2.fastq.gz
Usf1	wgEncodeHaibTfbsK562Usf1V0416101RawDataRep1.fastq.gz, wgEncodeHaibTfbsK562Usf1V0416101RawDataRep2.fastq.gz	wgEncodeHaibTfbsK562RxlchV0416101RawDataRep1.fastq.gz, wgEncodeHaibTfbsK562RxlchV0416101RawDataRep2.fastq.gz
Usf2	wgEncodeSydhTfbsK562Usf2IggrabRawDataRep1.fastq.gz, wgEncodeSydhTfbsK562Usf2IggrabRawDataRep2.fastq.gz	SydhK562InputStdAlnRepRep1.fastq.gz, SydhK562InputStdAlnRepRep2.fastq.gz
Yy1	wgEncodeHaibTfbsK562Yy1V0416101RawDataRep1.fastq.gz*, wgEncodeHaibTfbsK562Yy1V0416101RawDataRep2.fastq.gz*	wgEncodeHaibTfbsK562RxlchV0416101RawDataRep1.fastq.gz, wgEncodeHaibTfbsK562RxlchV0416101RawDataRep2.fastq.gz
Zbtb33	wgEncodeHaibTfbsK562Zbtb33Pcr1xRawDataRep1.fastq.gz, wgEncodeHaibTfbsK562Zbtb33Pcr1xRawDataRep2.fastq.gz	wgEncodeHaibTfbsK562RxlchV0416101RawDataRep1.fastq.gz, wgEncodeHaibTfbsK562RxlchV0416101RawDataRep2.fastq.gz

Supplementary Table 10. ChIP and Input fastq files for K562.

Histone	GM12878 ChIP samples
H2az	wgEncodeBroadHistoneGm12878H2azStdRawDataRep1.fastq.gz
	wgEncodeBroadHistoneGm12878H2azStdRawDataRep2.fastq.gz
H3k27ac	wgEncodeBroadHistoneGm12878H3k27acStdRawDataRep1.fastq.gz
	wgEncodeBroadHistoneGm12878H3k27acStdRawDataRep2.fastq.gz
H3k27me3	wgEncodeBroadHistoneGm12878H3k27me3StdRawDataRep1.fastq.gz
	wgEncodeBroadHistoneGm12878H3k27me3StdRawDataRep2.fastq.gz
H3k36me3	wgEncodeBroadHistoneGm12878H3k36me3StdRawDataRep1.fastq.gz
	wgEncodeBroadHistoneGm12878H3k36me3StdRawDataRep2.fastq.gz
H3k4me1	wgEncodeBroadHistoneGm12878H3k4me1StdRawDataRep1.fastq.gz
	wgEncodeBroadHistoneGm12878H3k4me1StdRawDataRep2.fastq.gz
H3k4me2	wgEncodeBroadHistoneGm12878H3k4me2StdRawDataRep1.fastq.gz
	wgEncodeBroadHistoneGm12878H3k4me2StdRawDataRep2.fastq.gz
H3k4me3	wgEncodeBroadHistoneGm12878H3k4me3StdRawDataRep1.fastq.gz
	wgEncodeBroadHistoneGm12878H3k4me3StdRawDataRep2.fastq.gz
H3k79me2	wgEncodeBroadHistoneGm12878H3k79me2StdRawDataRep1.fastq.gz
	wgEncodeBroadHistoneGm12878H3k79me2StdRawDataRep2.fastq.gz
H3k9ac	wgEncodeBroadHistoneGm12878H3k9acStdRawDataRep1.fastq.gz
	wgEncodeBroadHistoneGm12878H3k9acStdRawDataRep2.fastq.gz
H3k9me3	wgEncodeBroadHistoneGm12878H3k9me3StdRawDataRep1.fastq.gz
	wgEncodeBroadHistoneGm12878H3k9me3StdRawDataRep2.fastq.gz
H4k20me1	wgEncodeBroadHistoneGm12878H4k20me1StdRawDataRep1.fastq.gz
	wgEncodeBroadHistoneGm12878H4k20me1StdRawDataRep2.fastq.gz

Huvec ChIP samples
wgEncodeSydhTfbsHuvecGata2UcdRawDataRep1.fastq.gz
wgEncodeSydhTfbsHuvecGata2UcdRawDataRep2.fastq.gz

GM12878 RRBS data
wgEncodeHaibMethylRrbsGm12878HaibSitesRep1.bed
wgEncodeHaibMethylRrbsGm12878HaibSitesRep2.bed

Supplementary Table 11. Histone ChIP and DNase fastq files for GM12878 and K562.

References

[1] Wang J, Huda A, Lunyak VV, Jordan IK (2010) A Gibbs sampling strategy applied to the mapping of ambiguous short-sequence tags. Bioinformatics 26: 2501-2508.
