# Supplementary material for: Perm-seq: Mapping Protein-DNA Interactions in Segmental Duplication and Highly Repetitive Regions of Genomes with Prior-Enhanced Read Mapping
Source: PLoS Comput Biol. 2015 Oct 20;11(10):e1004491. doi: 10.1371/journal.pcbi.1004491 (PMC4618727; doi:10.1371/journal.pcbi.1004491)
Supplement: S2 Text — (PDF) [file pcbi.1004491.s002.pdf]

Supplementary File 2 for "Mapping protein-DNA interactions in segmental duplication and highly repetitive regions of genomes with prior-enhanced read mapping"

Xin Zeng[1], Bo Li[3], Rene Welch[1], Colin Dewey[2] & Sündüz Keleş[1,2]
[1] Department of Statistics, University of Wisconsin, Madison, WI, U.S.A.
[2] Department of Biostatistics and Medical Informatics, University of Wisconsin, Madison, WI, U.S.A.
[3] California Institute for Quantitative Biosciences, University of California, Berkeley, CA, U.S.A.

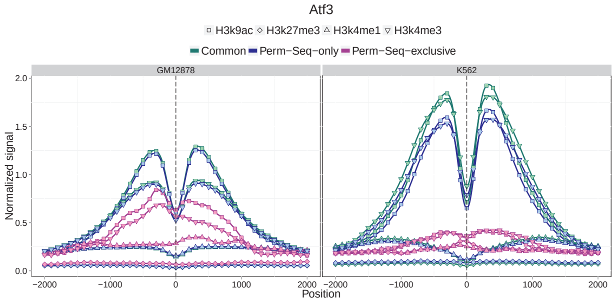
Atf3
H3k9ac H3k27me3 H3k4me1 H3k4me3
Common Perm−Seq−only Perm−Seq−exclusive
GM12878
K562
Normalized signal
Position
0.0 0.5 1.0 1.5 2.0
−2000 −1000 0 1000 2000

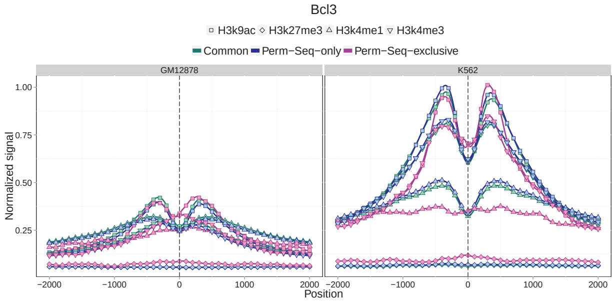
Bcl3
H3k9ac H3k27me3 H3k4me1 H3k4me3
Common Perm−Seq−only Perm−Seq−exclusive
GM12878
K562
Normalized signal
Position
0.25 0.50 0.75 1.00
−2000 −1000 0 1000 2000

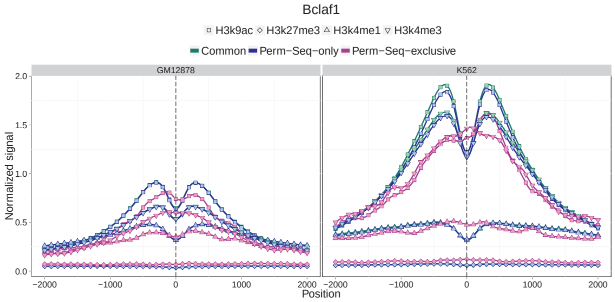
Bclaf1
H3k9ac H3k27me3 H3k4me1 H3k4me3
Common Perm−Seq−only Perm−Seq−exclusive
GM12878
K562
Normalized signal
Position
0.0 0.5 1.0 1.5 2.0
−2000 −1000 0 1000 2000

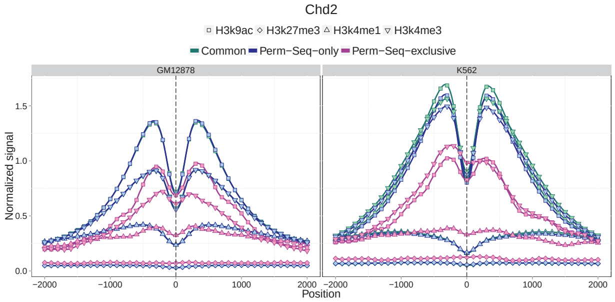
Chd2
H3k9ac H3k27me3 H3k4me1 H3k4me3
Common Perm−Seq−only Perm−Seq−exclusive
GM12878
K562
Normalized signal
Position
0.0 0.5 1.0 1.5
−2000 −1000 0 1000 2000

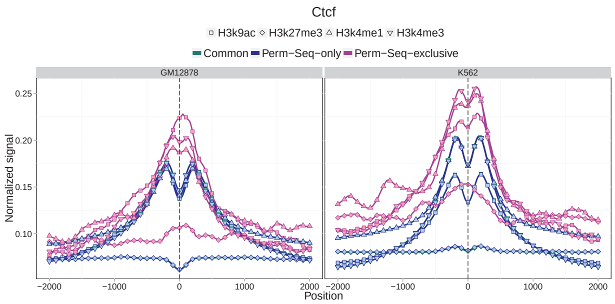
Ctcf
H3k9ac H3k27me3 H3k4me1 H3k4me3
Common Perm−Seq−only Perm−Seq−exclusive
GM12878
K562
Normalized signal
Position

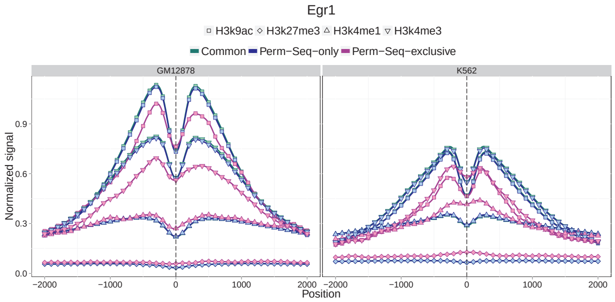
Egr1
H3k9ac H3k27me3 H3k4me1 H3k4me3
Common Perm−Seq−only Perm−Seq−exclusive
GM12878
K562
Normalized signal
Position

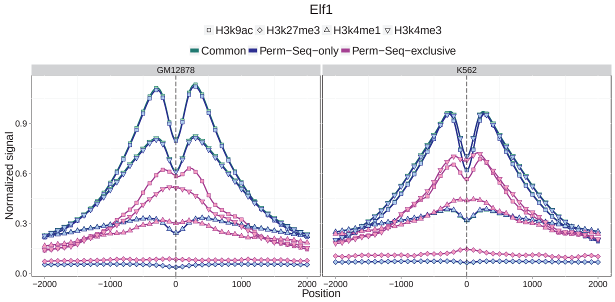
Elf1
H3k9ac H3k27me3 H3k4me1 H3k4me3
Common Perm−Seq−only Perm−Seq−exclusive
GM12878
K562
Normalized signal
Position

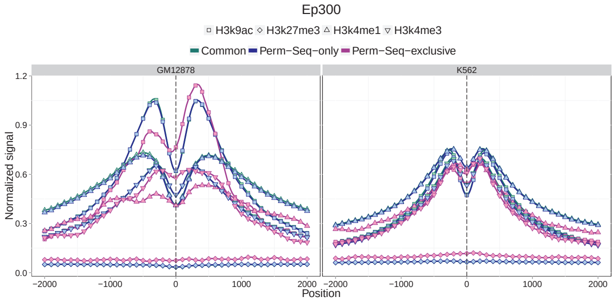
Ep300
H3k9ac H3k27me3 H3k4me1 H3k4me3
Common Perm−Seq−only Perm−Seq−exclusive
GM12878
K562
Normalized signal
Position

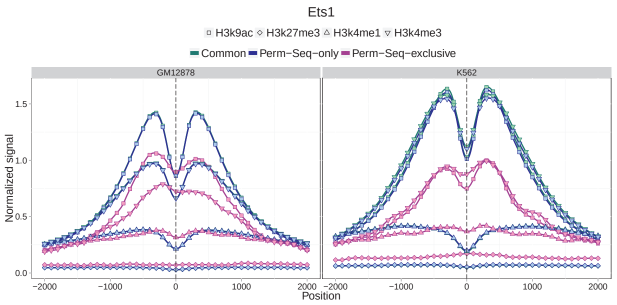
Ets1
H3k9ac H3k27me3 H3k4me1 H3k4me3
Common Perm−Seq−only Perm−Seq−exclusive
GM12878
K562
Normalized signal
Position

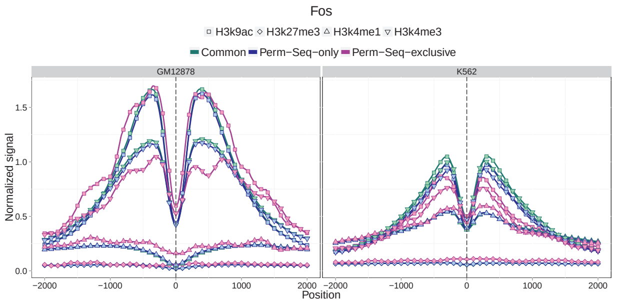
Fos
H3k9ac H3k27me3 H3k4me1 H3k4me3
Common Perm−Seq−only Perm−Seq−exclusive
GM12878
K562
Normalized signal
Position

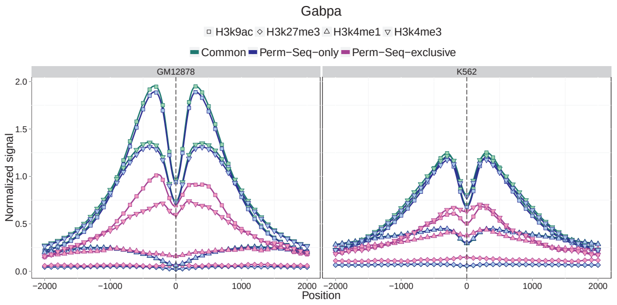
Gabpa
H3k9ac H3k27me3 H3k4me1 H3k4me3
Common Perm−Seq−only Perm−Seq−exclusive
GM12878
K562
Normalized signal
Position

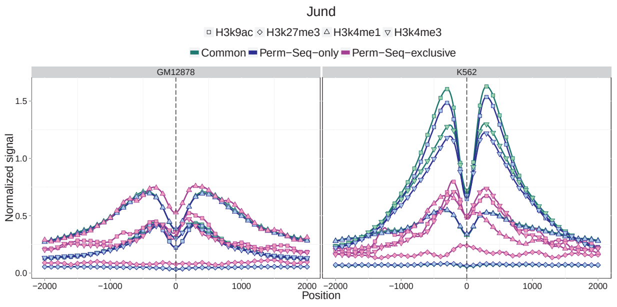
Jund
H3k9ac H3k27me3 H3k4me1 H3k4me3
Common Perm−Seq−only Perm−Seq−exclusive
GM12878
K562
Normalized signal
Position

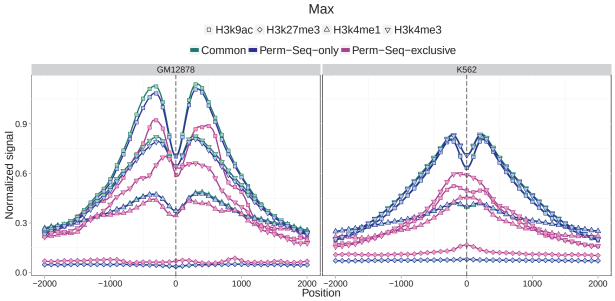
Max
H3k9ac H3k27me3 H3k4me1 H3k4me3
Common Perm−Seq−only Perm−Seq−exclusive
GM12878
K562
Normalized signal
Position
0.0 0.3 0.6 0.9
−2000 −1000 0 1000 2000

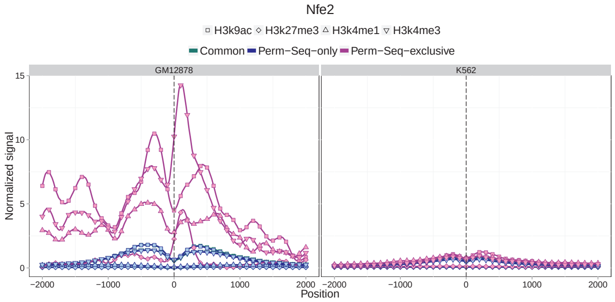
Nfe2
H3k9ac H3k27me3 H3k4me1 H3k4me3
Common Perm−Seq−only Perm−Seq−exclusive
GM12878
K562
Normalized signal
Position
0 5 10 15
−2000 −1000 0 1000 2000

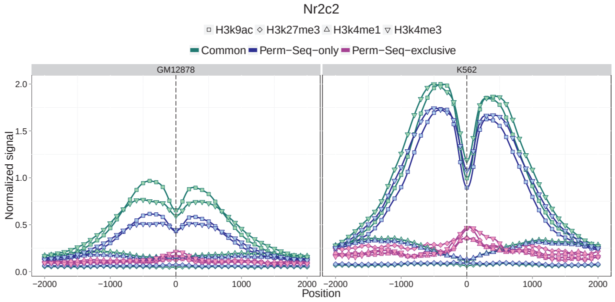
Nr2c2
H3k9ac H3k27me3 H3k4me1 H3k4me3
Common Perm−Seq−only Perm−Seq−exclusive
GM12878
K562
Normalized signal
Position
0.0 0.5 1.0 1.5 2.0
−2000 −1000 0 1000 2000

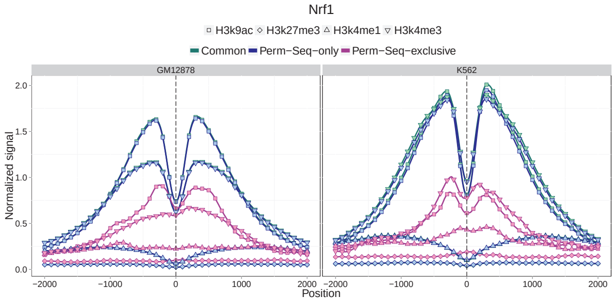
Nrf1
H3k9ac H3k27me3 H3k4me1 H3k4me3
Common Perm−Seq−only Perm−Seq−exclusive
GM12878
K562
Normalized signal
Position
0.0 0.5 1.0 1.5 2.0
−2000 −1000 0 1000 2000
