# Supplementary material for: Perm-seq: Mapping Protein-DNA Interactions in Segmental Duplication and Highly Repetitive Regions of Genomes with Prior-Enhanced Read Mapping
Source: PLoS Comput Biol. 2015 Oct 20;11(10):e1004491. doi: 10.1371/journal.pcbi.1004491 (PMC4618727; doi:10.1371/journal.pcbi.1004491)
Supplement: S3 Text — (PDF) [file pcbi.1004491.s003.pdf]

Supplementary File 3 for "Mapping protein-DNA interactions in segmental duplication and highly repetitive regions of genomes with prior-enhanced read mapping"

Xin Zeng[1], Bo Li[3], Rene Welch[1], Colin Dewey[2] & Sündüz Keleş[1,2]
[1] Department of Statistics, University of Wisconsin, Madison, WI, U.S.A.
[2] Department of Biostatistics and Medical Informatics, University of Wisconsin, Madison, WI, U.S.A.
[3] California Institute for Quantitative Biosciences, University of California, Berkeley, CA, U.S.A.

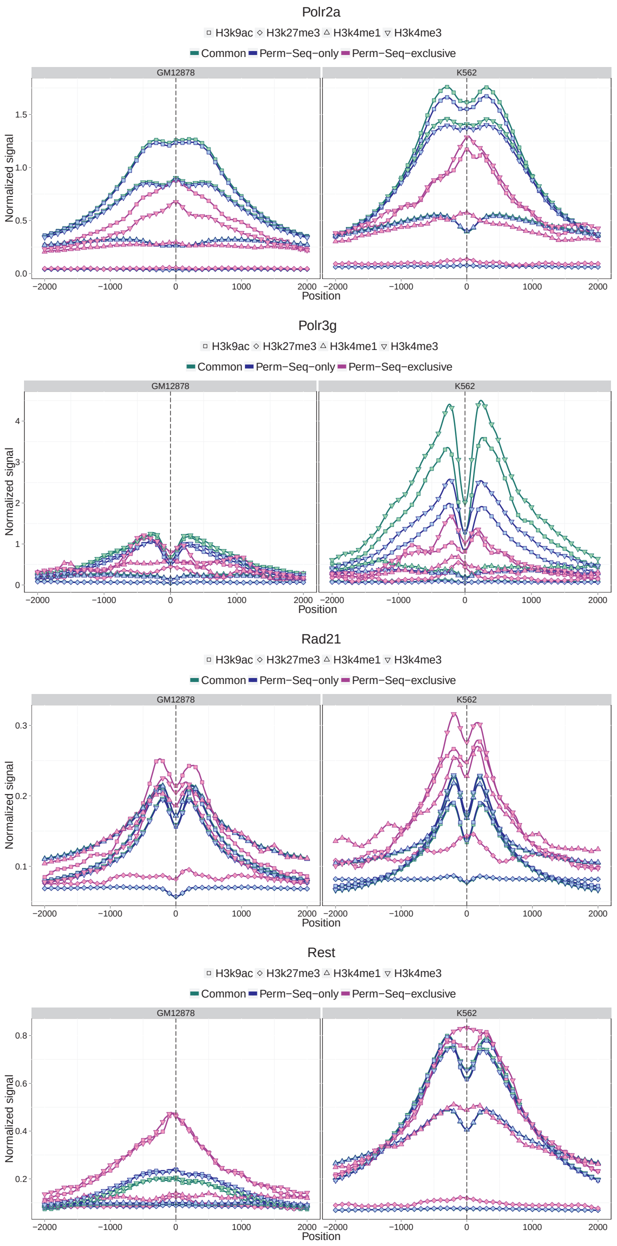
Polr2a
H3k9ac H3k27me3 H3k4me1 H3k4me3
Common Perm−Seq−only Perm−Seq−exclusive
GM12878
K562
Normalized signal
Position
Polr3g
H3k9ac H3k27me3 H3k4me1 H3k4me3
Common Perm−Seq−only Perm−Seq−exclusive
GM12878
K562
Normalized signal
Position
Rad21
H3k9ac H3k27me3 H3k4me1 H3k4me3
Common Perm−Seq−only Perm−Seq−exclusive
GM12878
K562
Normalized signal
Position
Rest
H3k9ac H3k27me3 H3k4me1 H3k4me3
Common Perm−Seq−only Perm−Seq−exclusive
GM12878
K562
Normalized signal
Position

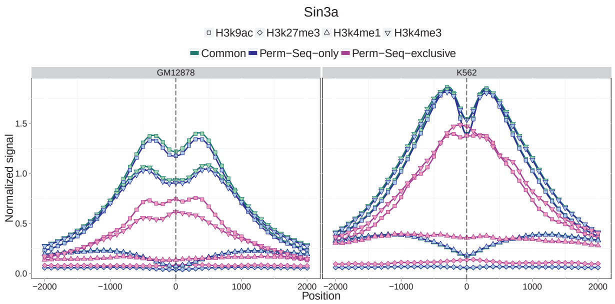
Sin3a
H3k9ac H3k27me3 H3k4me1 H3k4me3
Common Perm−Seq−only Perm−Seq−exclusive
GM12878
K562
Normalized signal
0.0
0.5
1.0
1.5
−2000
−1000
0
1000
2000
Position

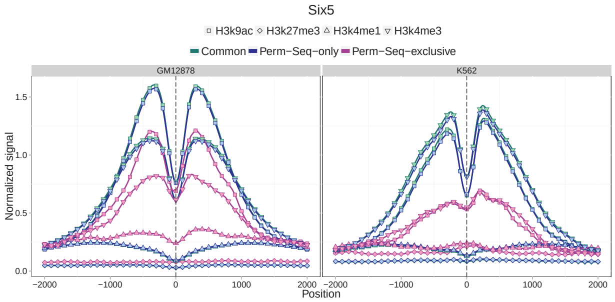
Six5
H3k9ac H3k27me3 H3k4me1 H3k4me3
Common Perm−Seq−only Perm−Seq−exclusive
GM12878
K562
Normalized signal
0.0
0.5
1.0
1.5
−2000
−1000
0
1000
2000
Position

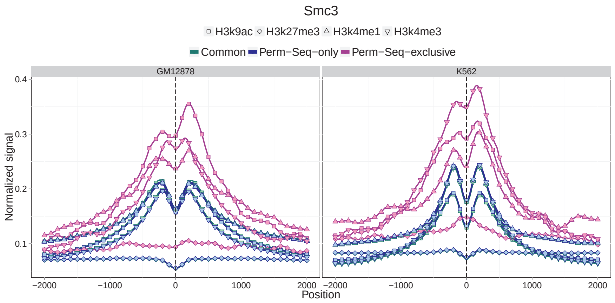
Smc3
H3k9ac H3k27me3 H3k4me1 H3k4me3
Common Perm−Seq−only Perm−Seq−exclusive
GM12878
K562
Normalized signal
0.1
0.2
0.3
0.4
−2000
−1000
0
1000
2000
Position

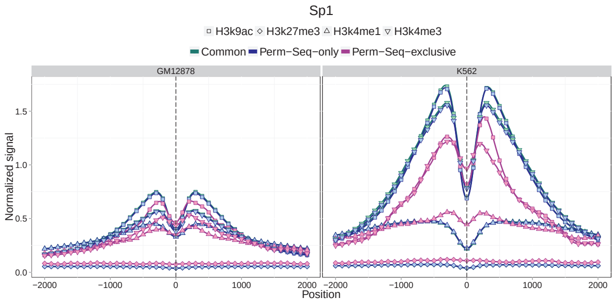
Sp1
H3k9ac H3k27me3 H3k4me1 H3k4me3
Common Perm−Seq−only Perm−Seq−exclusive
GM12878
K562
Normalized signal
0.0
0.5
1.0
1.5
−2000
−1000
0
1000
2000
Position

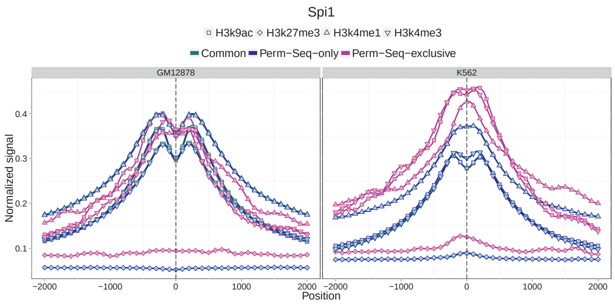
Spi1
H3k9ac H3k27me3 H3k4me1 H3k4me3
Common Perm−Seq−only Perm−Seq−exclusive
GM12878
K562
Normalized signal
Position

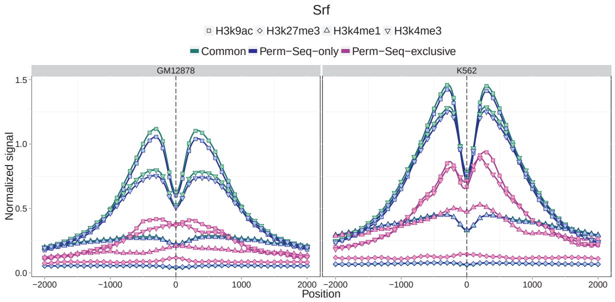
Srf
H3k9ac H3k27me3 H3k4me1 H3k4me3
Common Perm−Seq−only Perm−Seq−exclusive
GM12878
K562
Normalized signal
Position

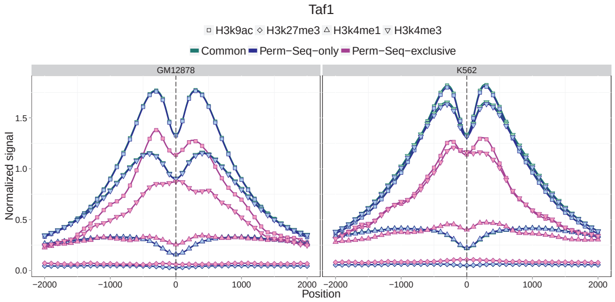
Taf1
H3k9ac H3k27me3 H3k4me1 H3k4me3
Common Perm−Seq−only Perm−Seq−exclusive
GM12878
K562
Normalized signal
Position

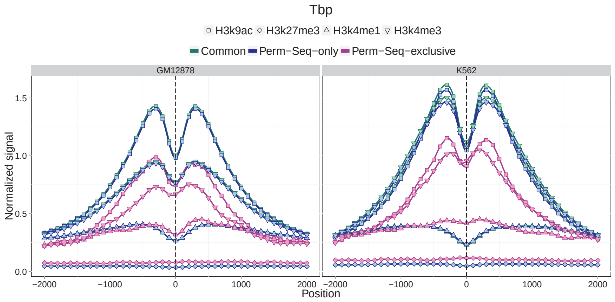
Tbp
H3k9ac H3k27me3 H3k4me1 H3k4me3
Common Perm−Seq−only Perm−Seq−exclusive
GM12878
K562
Normalized signal
Position

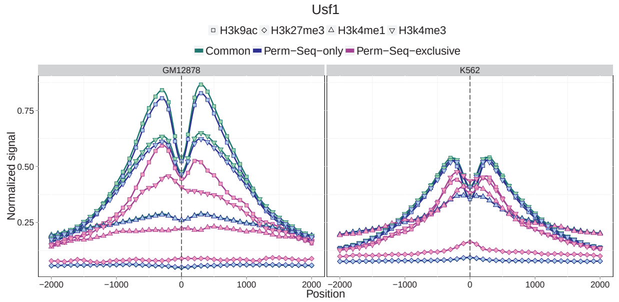
Usf1
H3k9ac H3k27me3 H3k4me1 H3k4me3
Common Perm−Seq−only Perm−Seq−exclusive
GM12878
K562
Normalized signal
0.25
0.50
0.75
−2000 −1000 0 1000 2000
Position

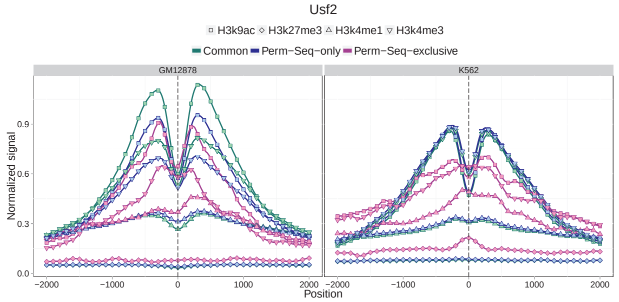
Usf2
H3k9ac H3k27me3 H3k4me1 H3k4me3
Common Perm−Seq−only Perm−Seq−exclusive
GM12878
K562
Normalized signal
0.0
0.3
0.6
0.9
−2000 −1000 0 1000 2000
Position

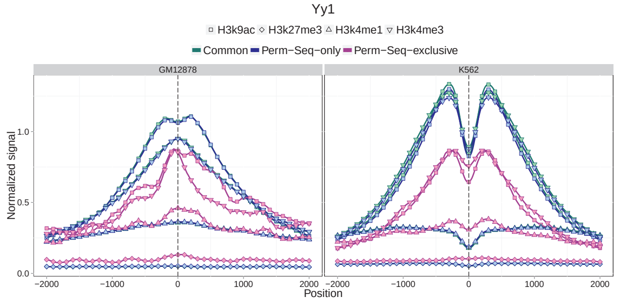
Yy1
H3k9ac H3k27me3 H3k4me1 H3k4me3
Common Perm−Seq−only Perm−Seq−exclusive
GM12878
K562
Normalized signal
0.0
0.5
1.0
−2000 −1000 0 1000 2000
Position

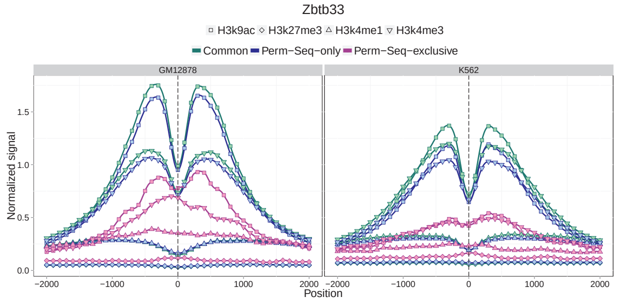
Zbtb33
H3k9ac H3k27me3 H3k4me1 H3k4me3
Common Perm−Seq−only Perm−Seq−exclusive
GM12878
K562
Normalized signal
0.0
0.5
1.0
1.5
−2000 −1000 0 1000 2000
Position
