# Supplementary material for: Perm-seq: Mapping Protein-DNA Interactions in Segmental Duplication and Highly Repetitive Regions of Genomes with Prior-Enhanced Read Mapping
Source: PLoS Comput Biol. 2015 Oct 20;11(10):e1004491. doi: 10.1371/journal.pcbi.1004491 (PMC4618727; doi:10.1371/journal.pcbi.1004491)
Supplement: S4 Text — (PDF) [file pcbi.1004491.s004.pdf]

Supplementary File 4 for "Mapping protein-DNA interactions in segmental duplication and highly repetitive regions of genomes with prior-enhanced read mapping"

Xin Zeng[1], Bo Li[3], Rene Welch[1], Colin Dewey[2] & Sündüz Keleş[1,2]
[1] Department of Statistics, University of Wisconsin, Madison, WI, U.S.A.
[2] Department of Biostatistics and Medical Informatics, University of Wisconsin, Madison, WI, U.S.A.
[3] California Institute for Quantitative Biosciences, University of California, Berkeley, CA, U.S.A.

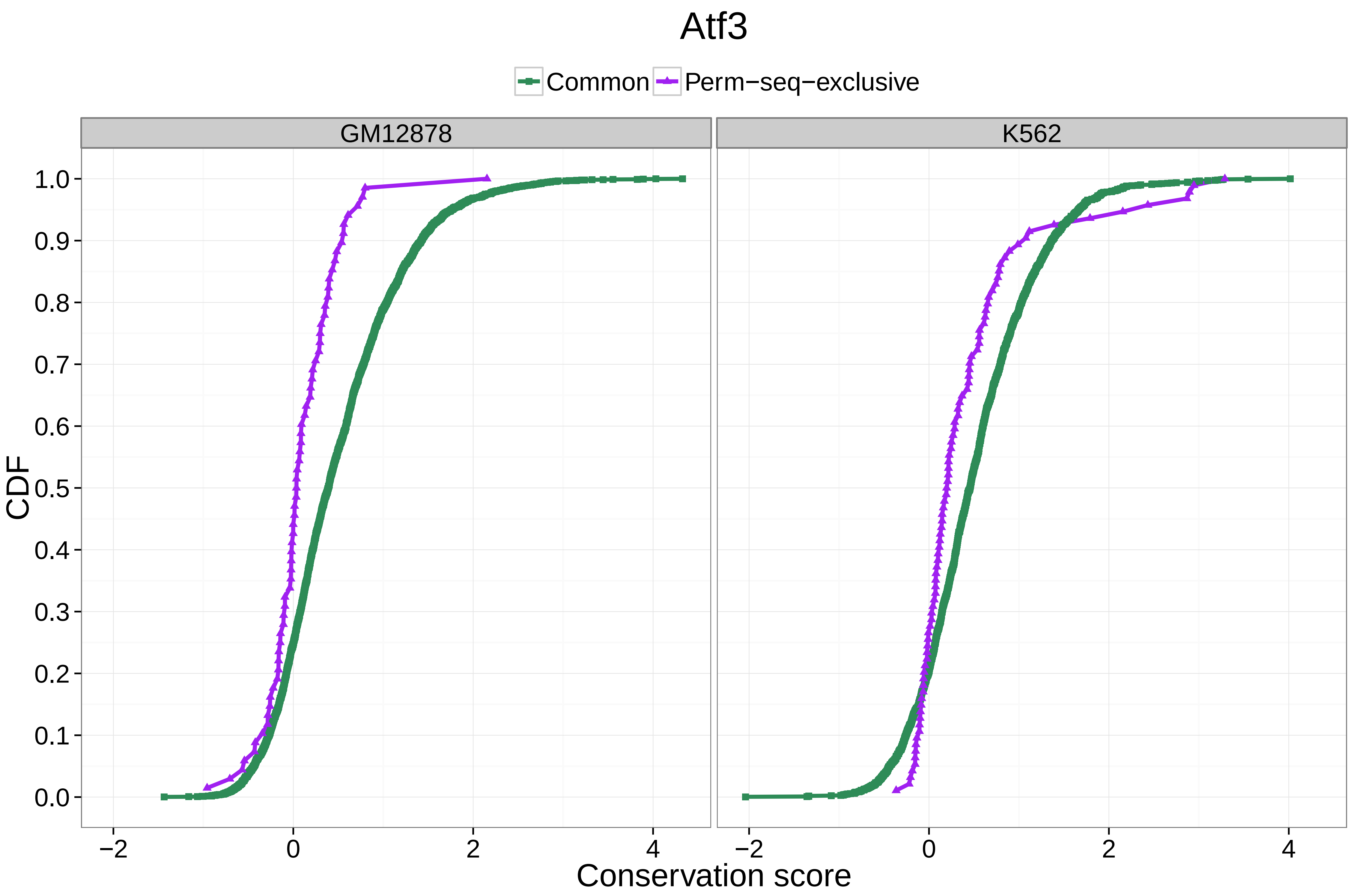
Atf3
Common
Perm−seq−exclusive
GM12878
K562
CDF
0.0
0.1
0.2
0.3
0.4
0.5
0.6
0.7
0.8
0.9
1.0
−2
0
2
4
Conservation score

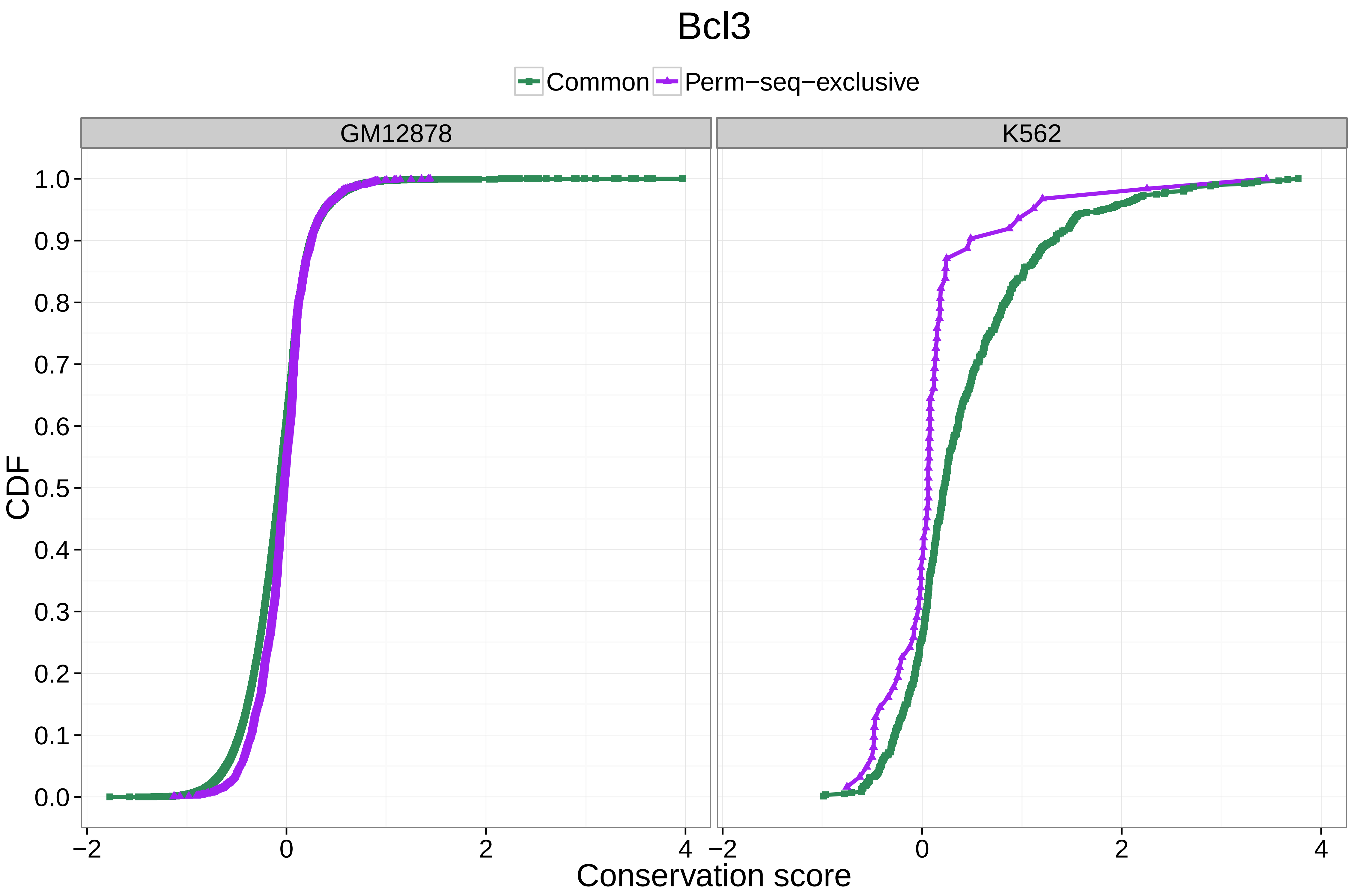

Bcl3
Common
Perm−seq−exclusive
GM12878
K562
CDF
1.0
0.9
0.8
0.7
0.6
0.5
0.4
0.3
0.2
0.1
0.0
−2
0
2
4
Conservation score

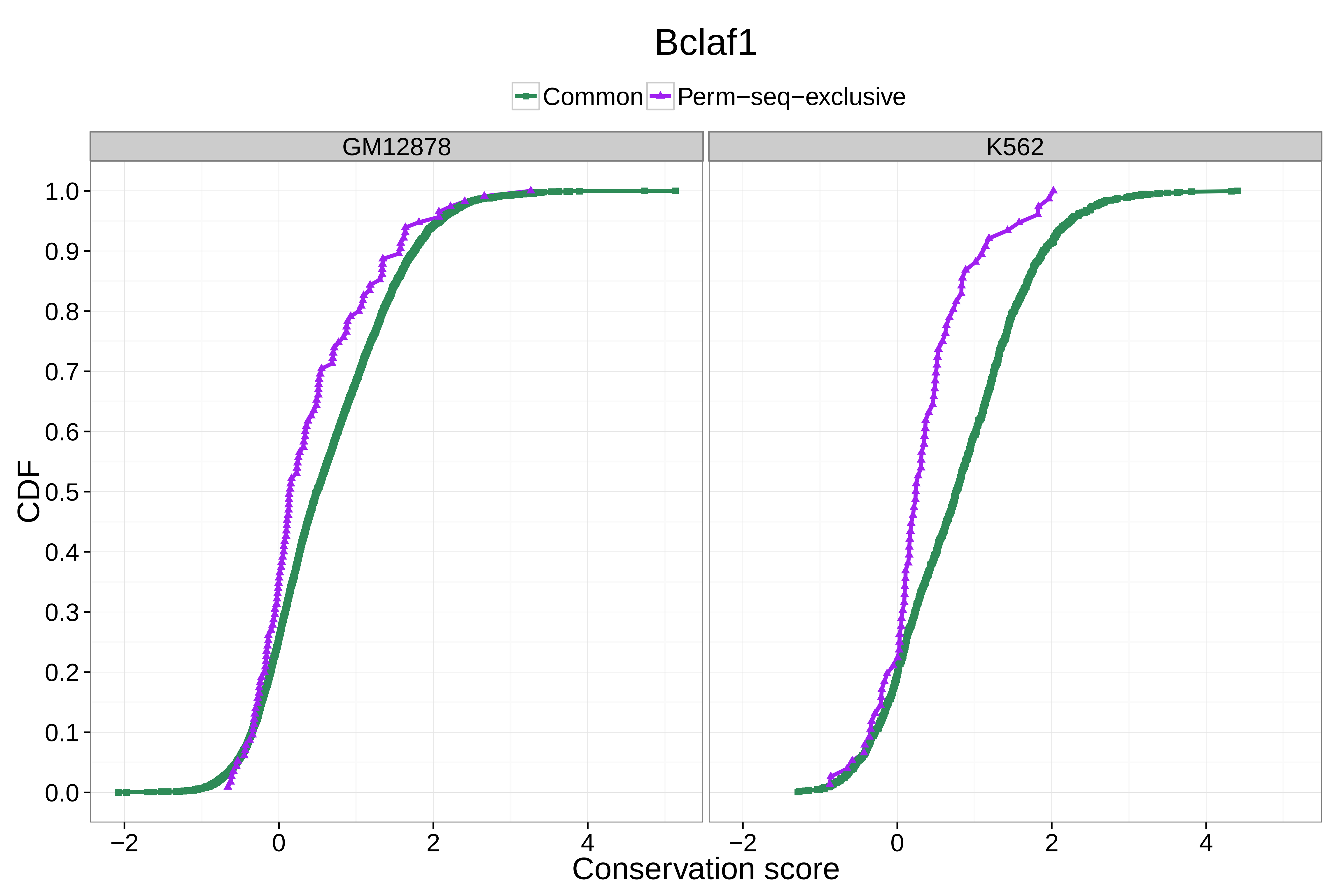

Bclaf1
Common
Perm−seq−exclusive
GM12878
K562
CDF
1.0
0.9
0.8
0.7
0.6
0.5
0.4
0.3
0.2
0.1
0.0
−2
0
2
4
Conservation score

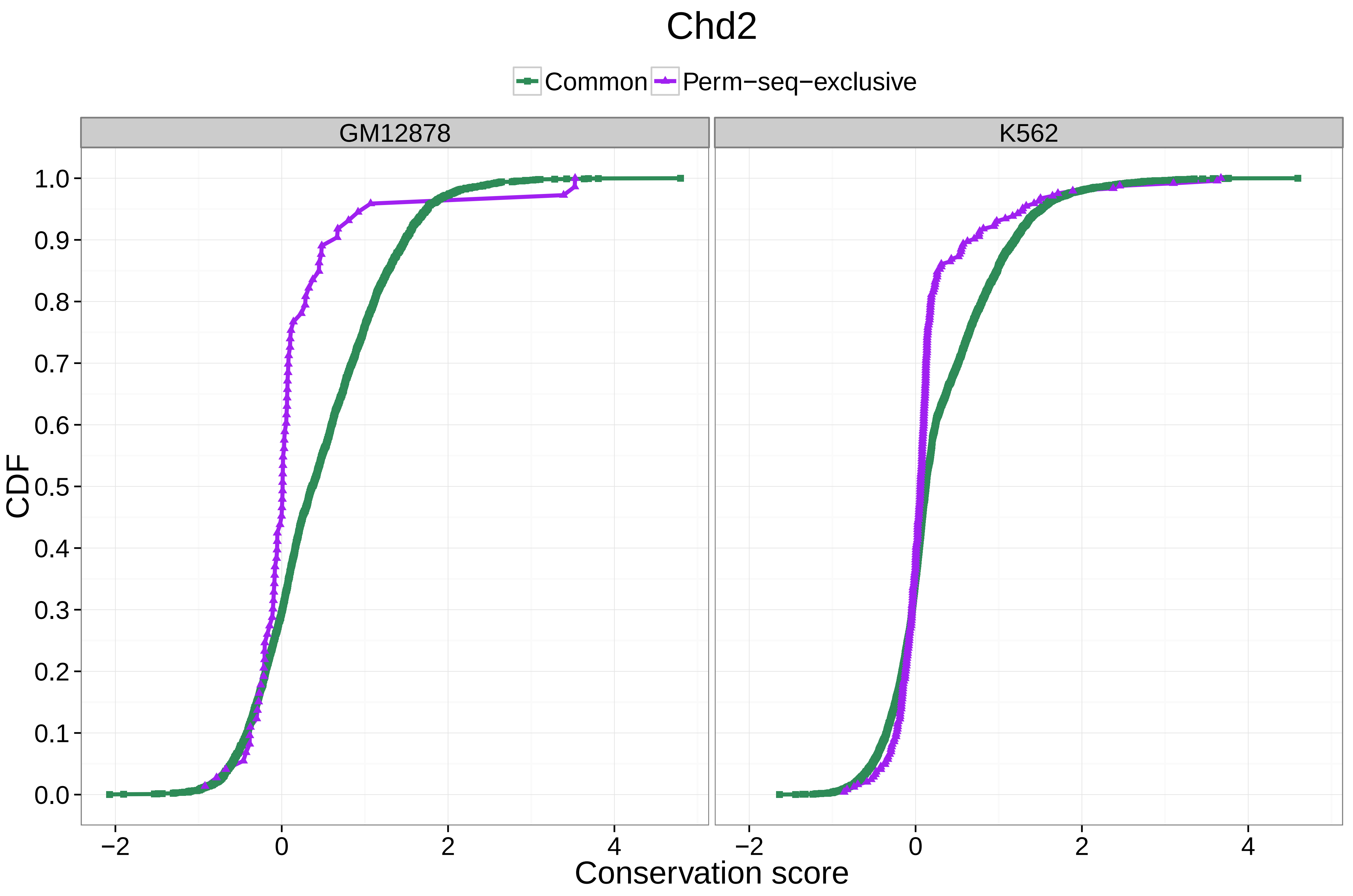
Chd2
Common
Perm−seq−exclusive
GM12878
K562
CDF
1.0
0.9
0.8
0.7
0.6
0.5
0.4
0.3
0.2
0.1
0.0
−2
0
2
4
Conservation score

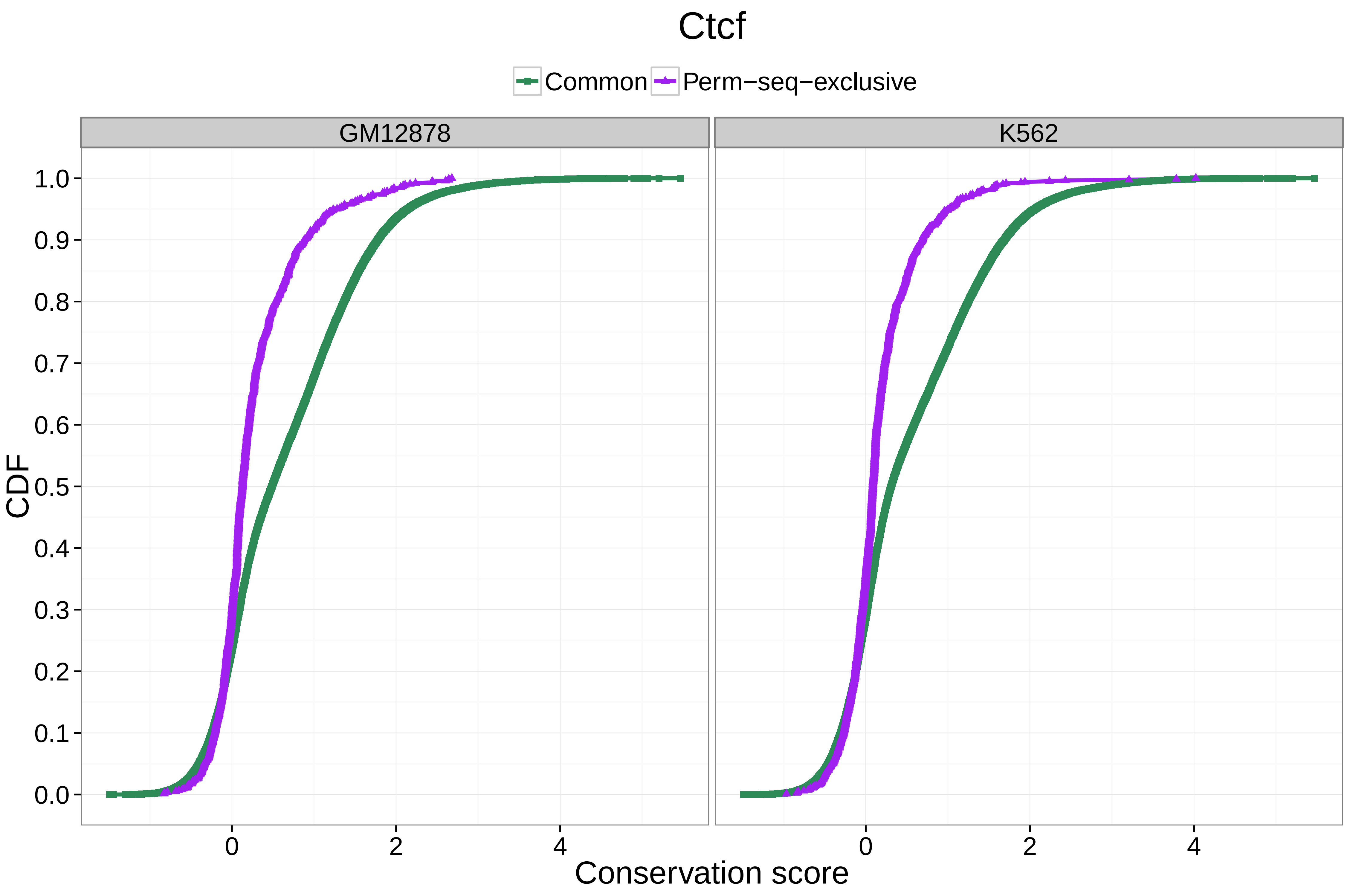

Ctcf
Common
Perm−seq−exclusive
GM12878
K562
CDF
1.0
0.9
0.8
0.7
0.6
0.5
0.4
0.3
0.2
0.1
0.0
0
2
4
Conservation score

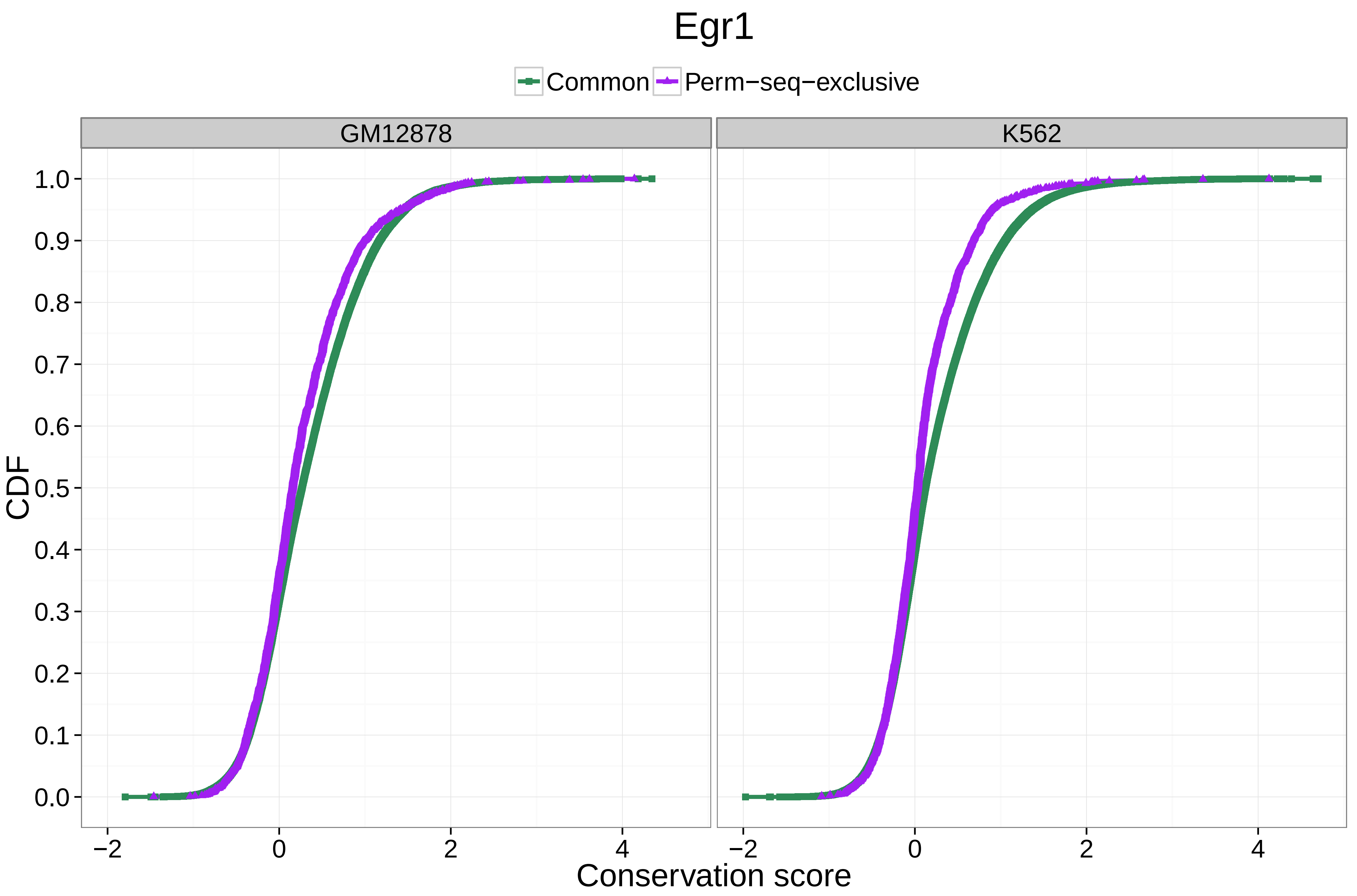
Egr1
Common
Perm−seq−exclusive
GM12878
K562
CDF
1.0
0.9
0.8
0.7
0.6
0.5
0.4
0.3
0.2
0.1
0.0
−2
0
2
4
Conservation score

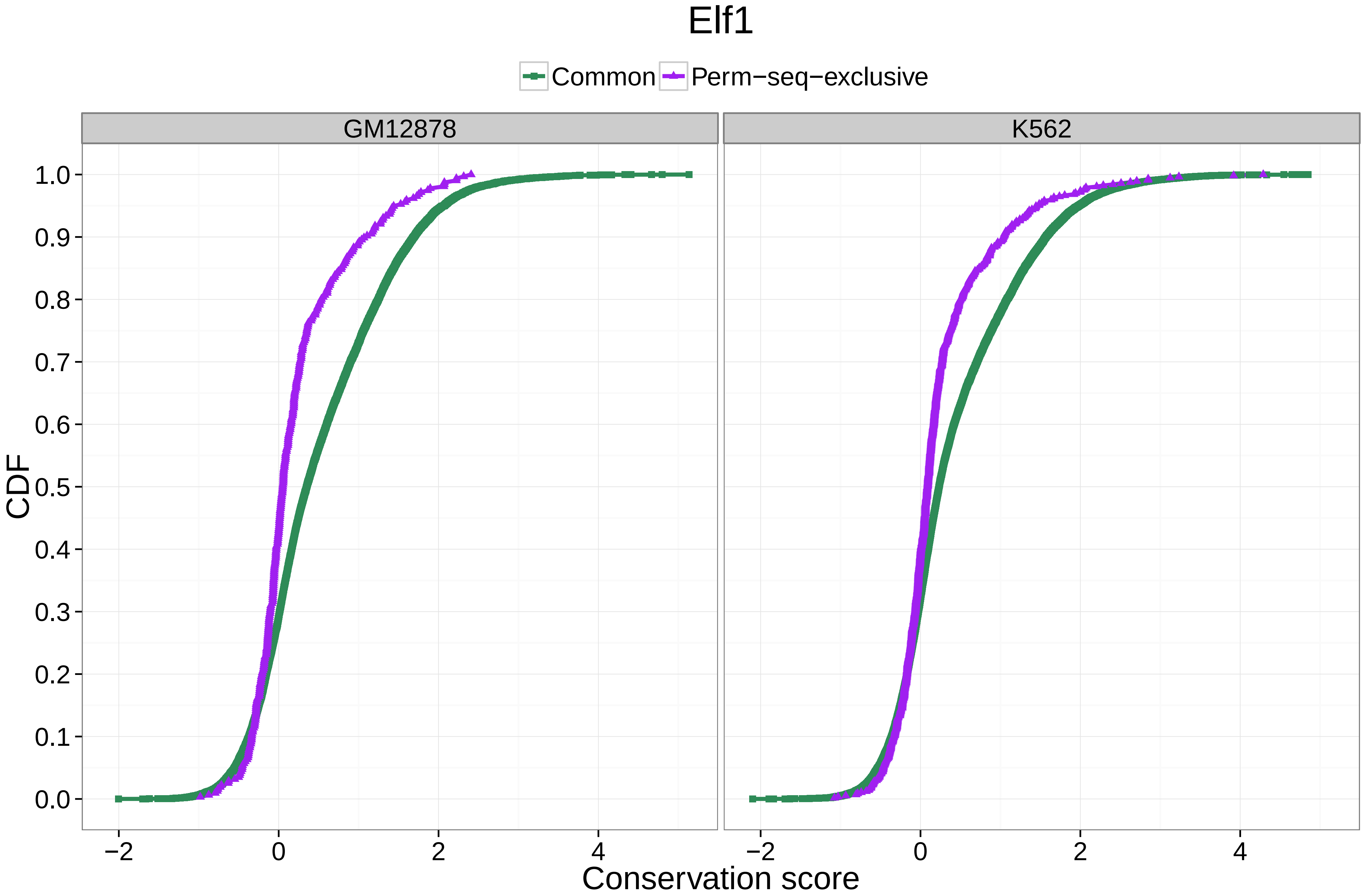
Elf1
Common
Perm−seq−exclusive
GM12878
K562
CDF
1.0
0.9
0.8
0.7
0.6
0.5
0.4
0.3
0.2
0.1
0.0
−2
0
2
4
Conservation score

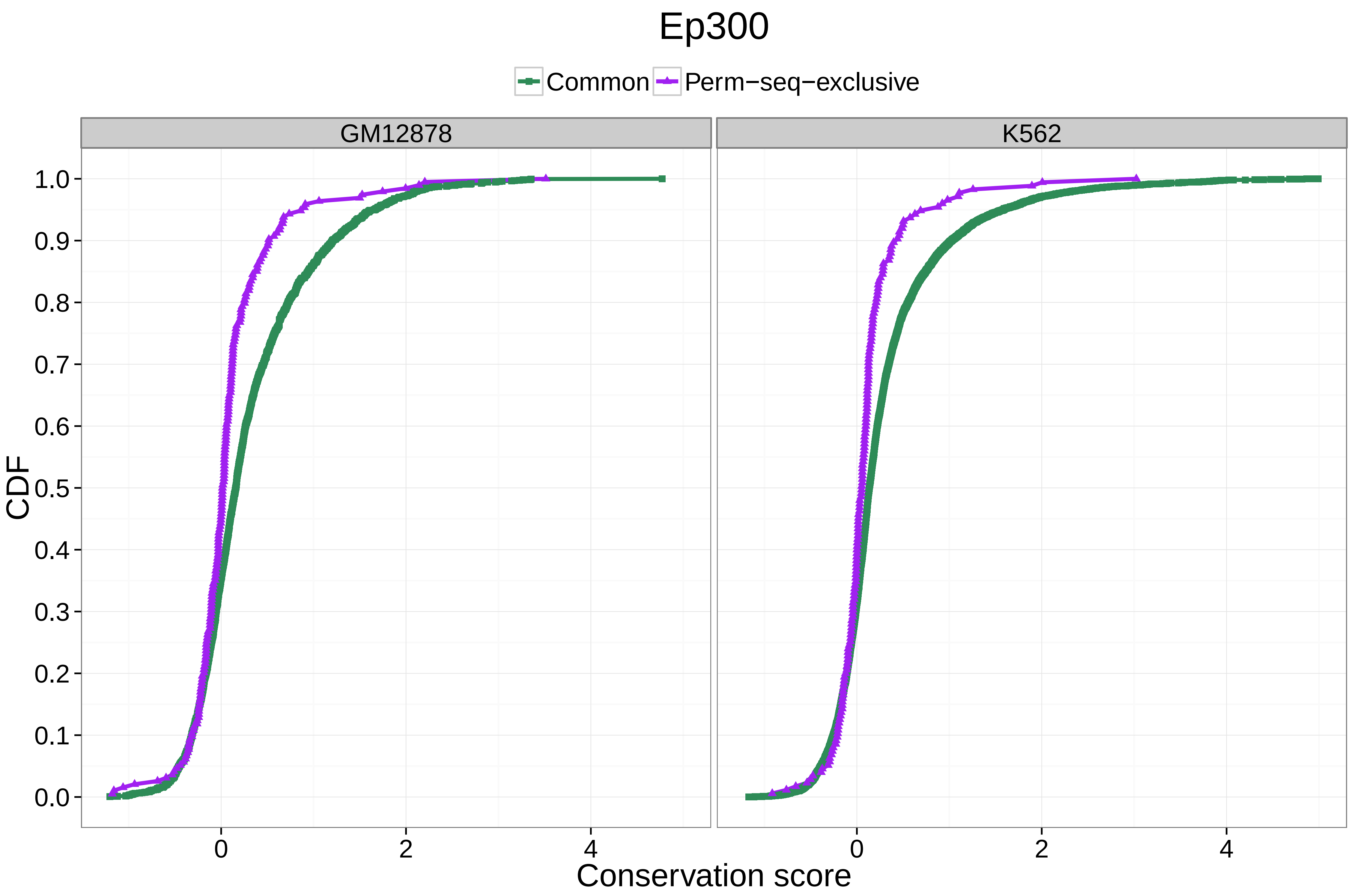
Ep300
Common
Perm−seq−exclusive
GM12878
K562
CDF
1.0
0.9
0.8
0.7
0.6
0.5
0.4
0.3
0.2
0.1
0.0
0
2
4
Conservation score

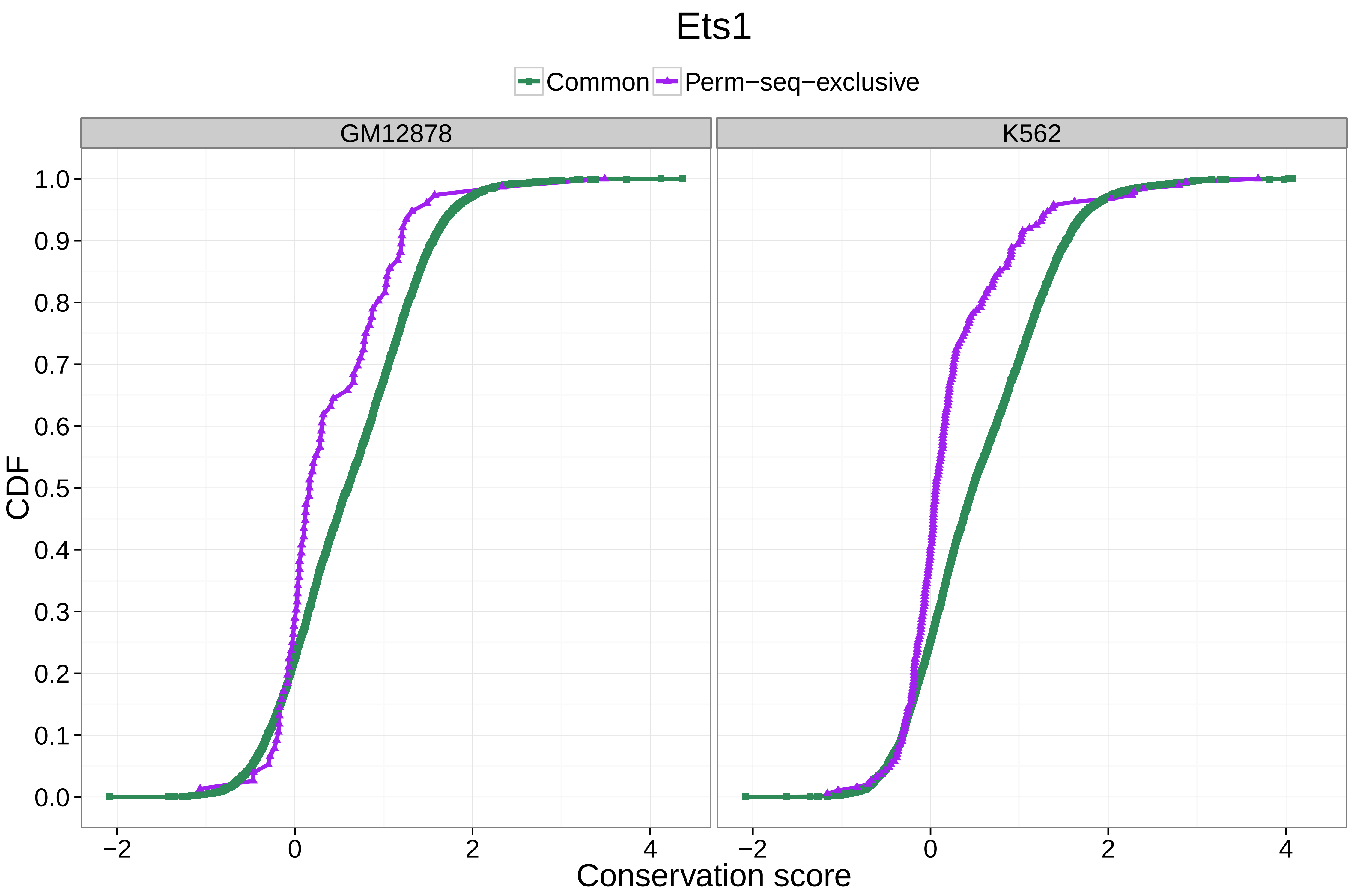

Ets1
Common
Perm−seq−exclusive
GM12878
K562
CDF
Conservation score
1.0
0.9
0.8
0.7
0.6
0.5
0.4
0.3
0.2
0.1
0.0
−2
0
2
4

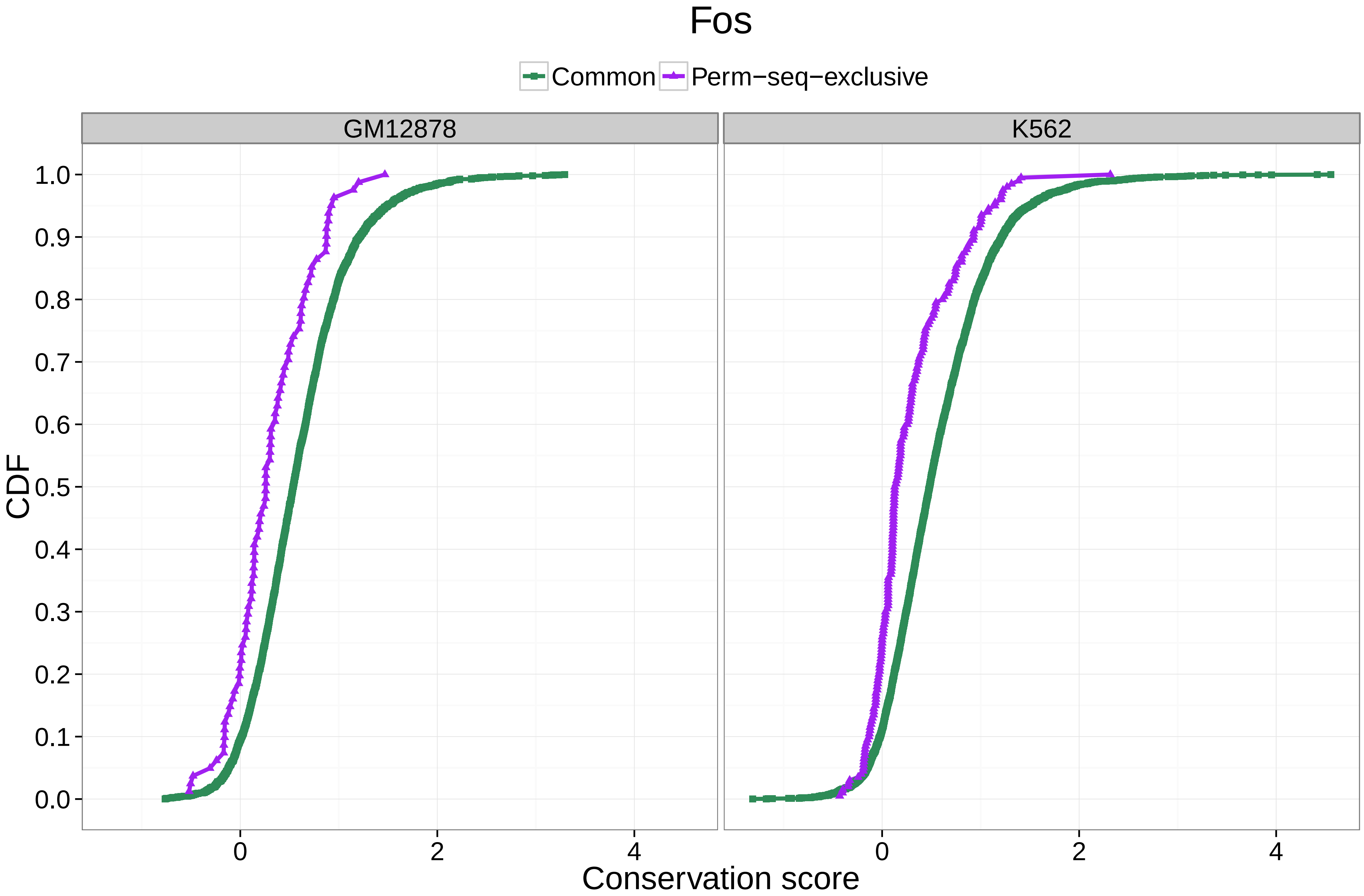
Fos
Common
Perm−seq−exclusive
GM12878
K562
CDF
1.0
0.9
0.8
0.7
0.6
0.5
0.4
0.3
0.2
0.1
0.0
0
2
4
0
2
4
Conservation score

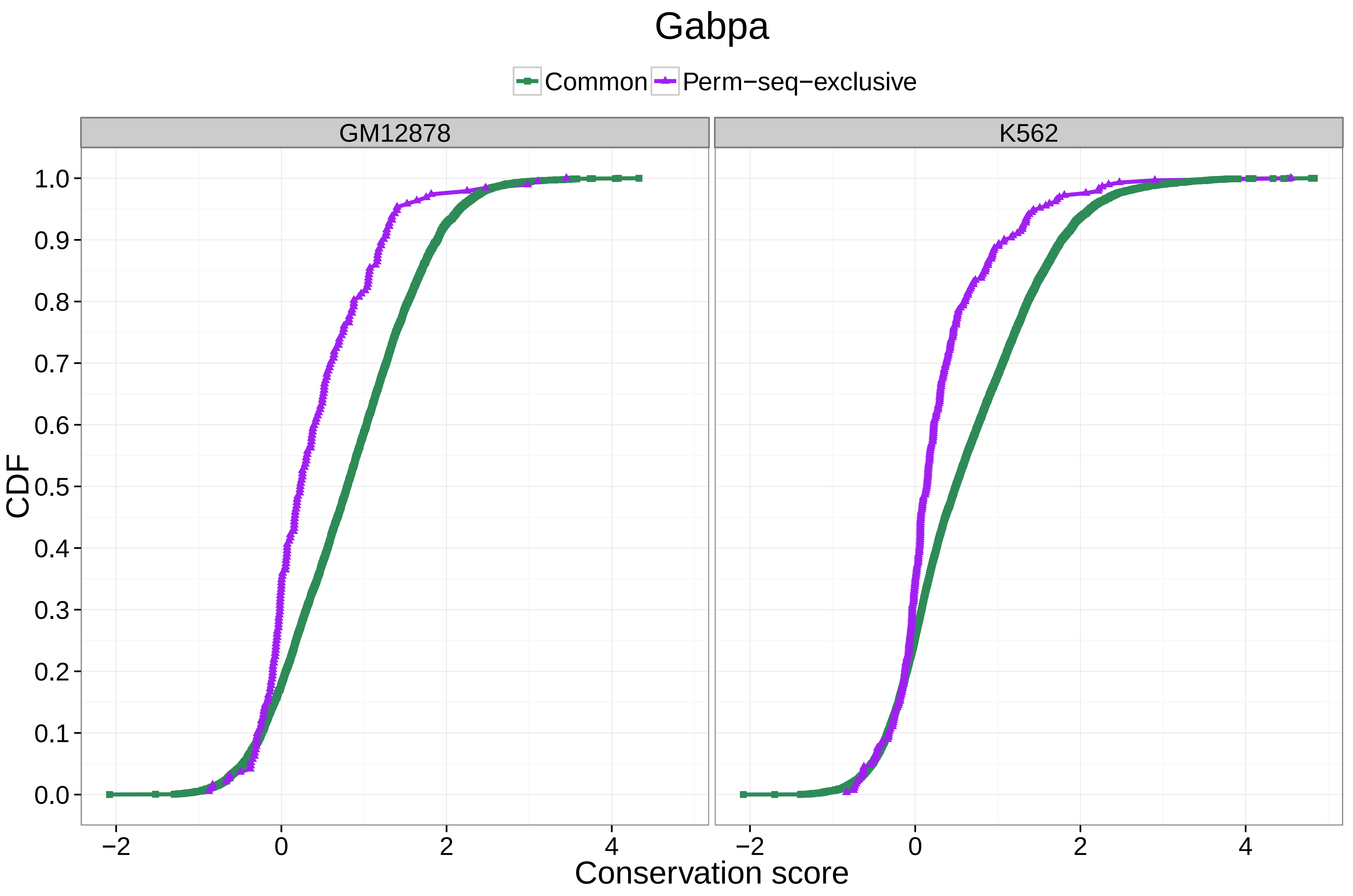

Gabpa
Common
Perm−seq−exclusive
GM12878
K562
CDF
1.0
0.9
0.8
0.7
0.6
0.5
0.4
0.3
0.2
0.1
0.0
−2
0
2
4
Conservation score

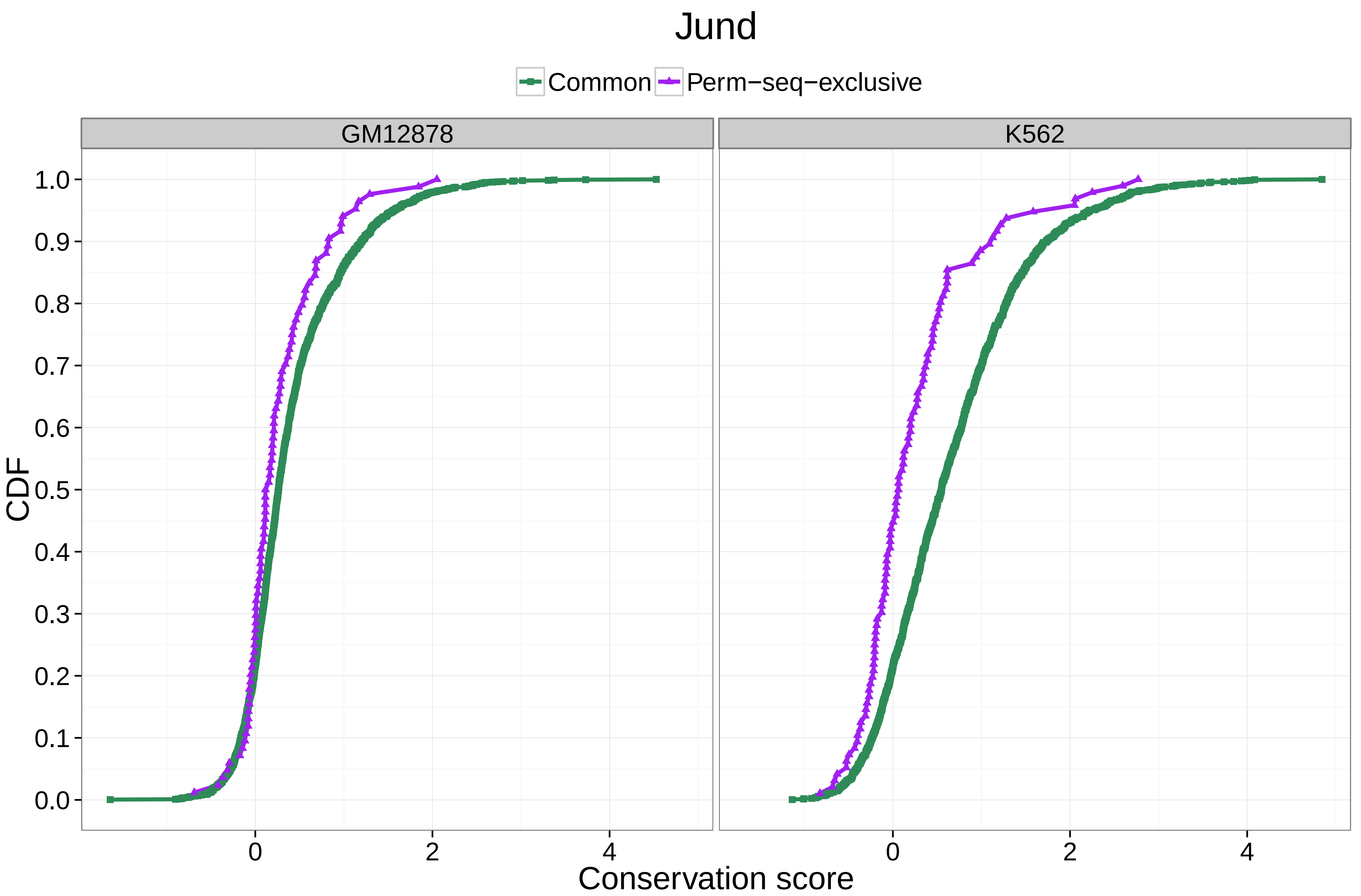
Jund
Common
Perm−seq−exclusive
GM12878
K562
CDF
1.0
0.9
0.8
0.7
0.6
0.5
0.4
0.3
0.2
0.1
0.0
0
2
4
Conservation score

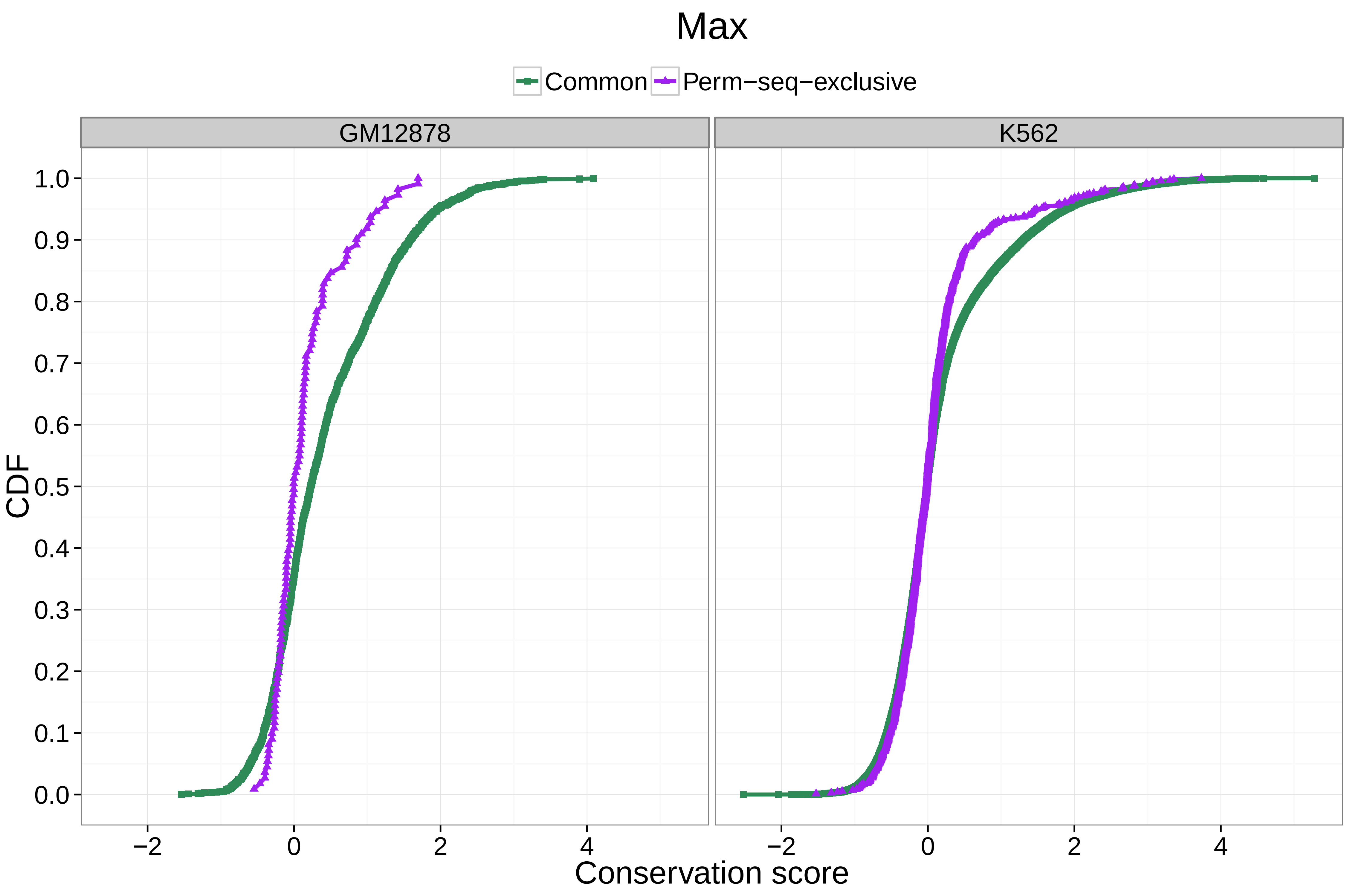

Max
Common
Perm−seq−exclusive
GM12878
K562
CDF
0.0
0.1
0.2
0.3
0.4
0.5
0.6
0.7
0.8
0.9
1.0
−2
0
2
4
Conservation score

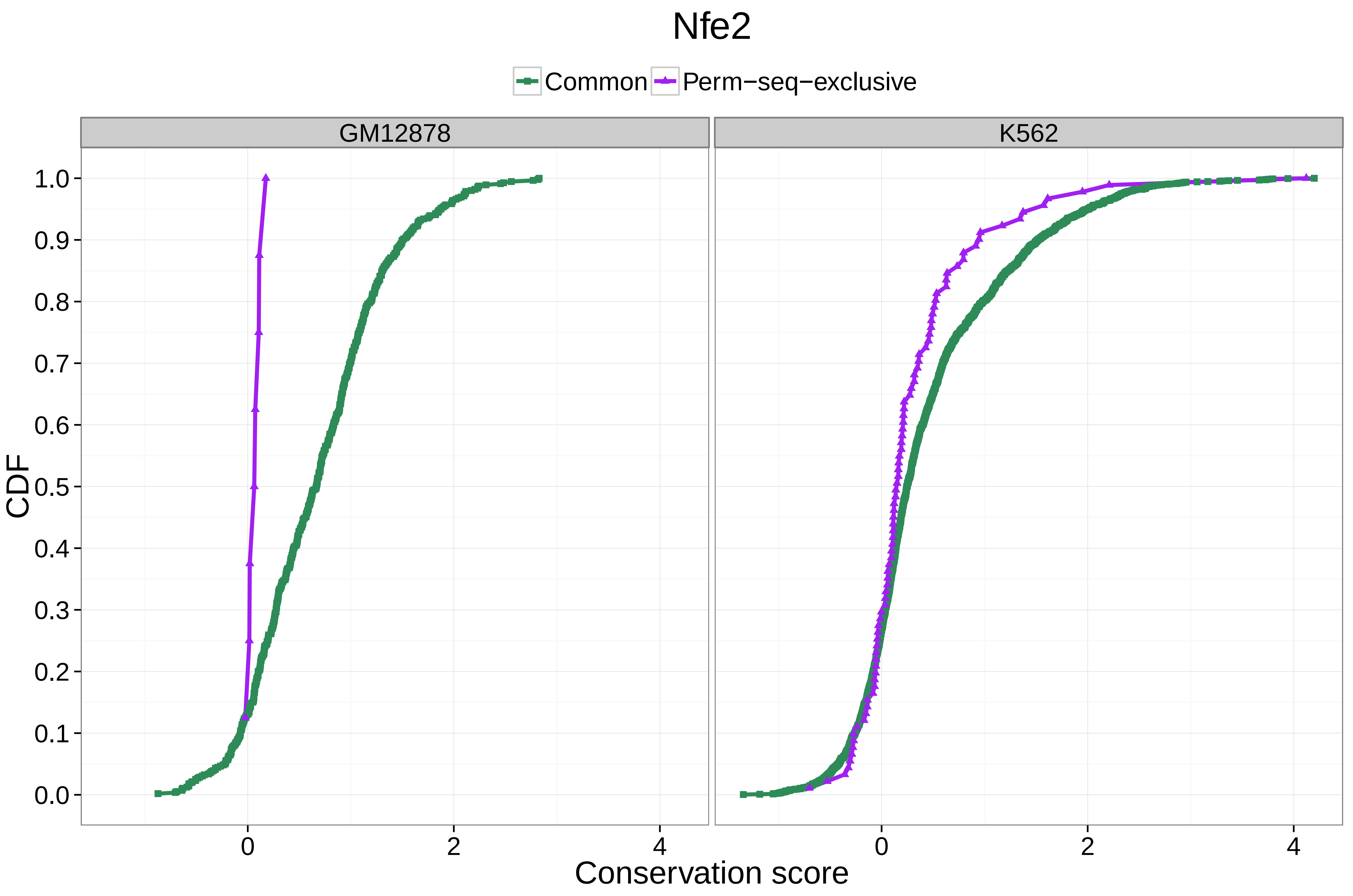

Nfe2
Common
Perm−seq−exclusive
GM12878
K562
CDF
1.0
0.9
0.8
0.7
0.6
0.5
0.4
0.3
0.2
0.1
0.0
0
2
4
Conservation score

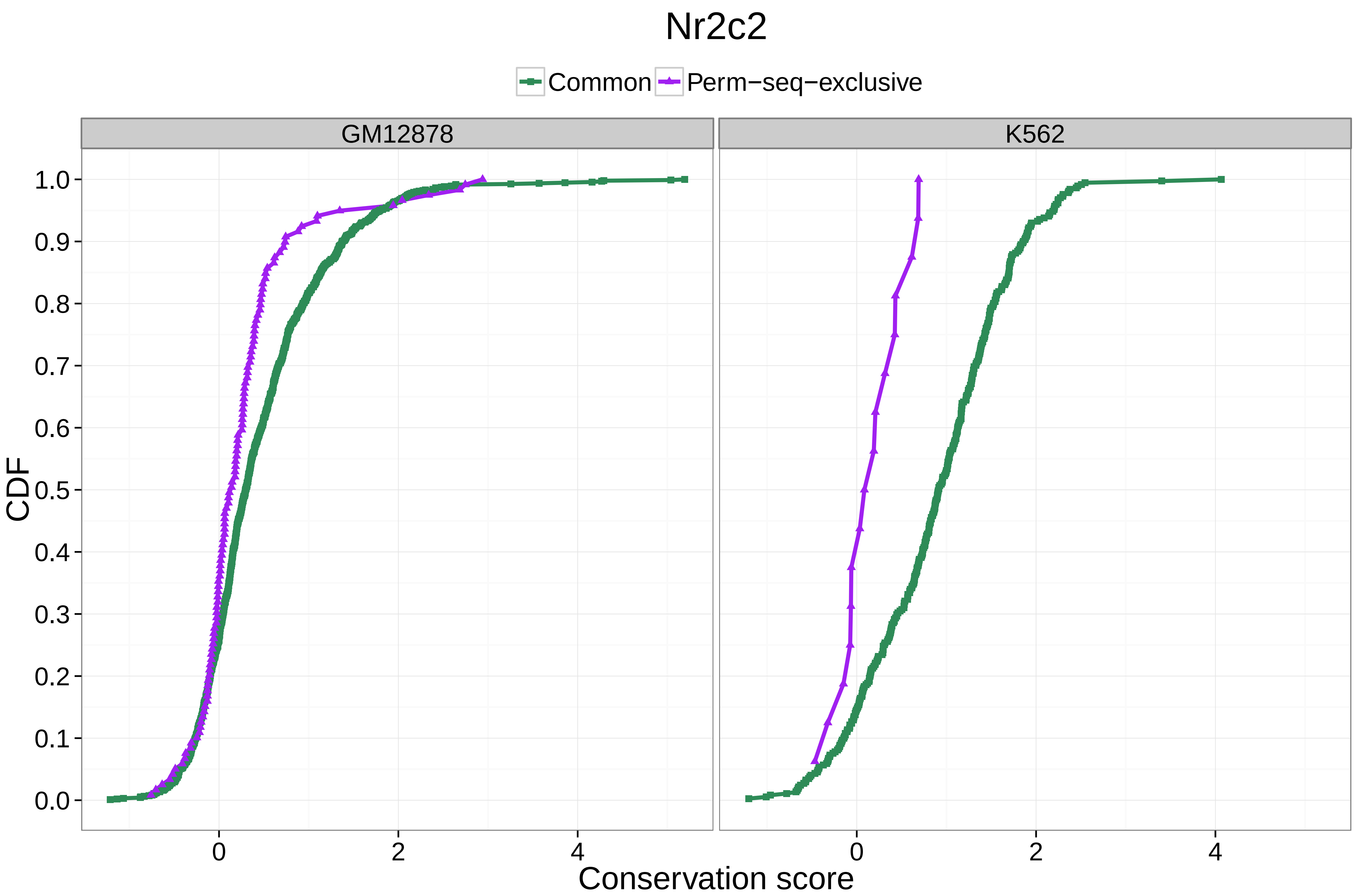
Nr2c2
Common
Perm−seq−exclusive
GM12878
K562
CDF
1.0
0.9
0.8
0.7
0.6
0.5
0.4
0.3
0.2
0.1
0.0
0
2
4
Conservation score

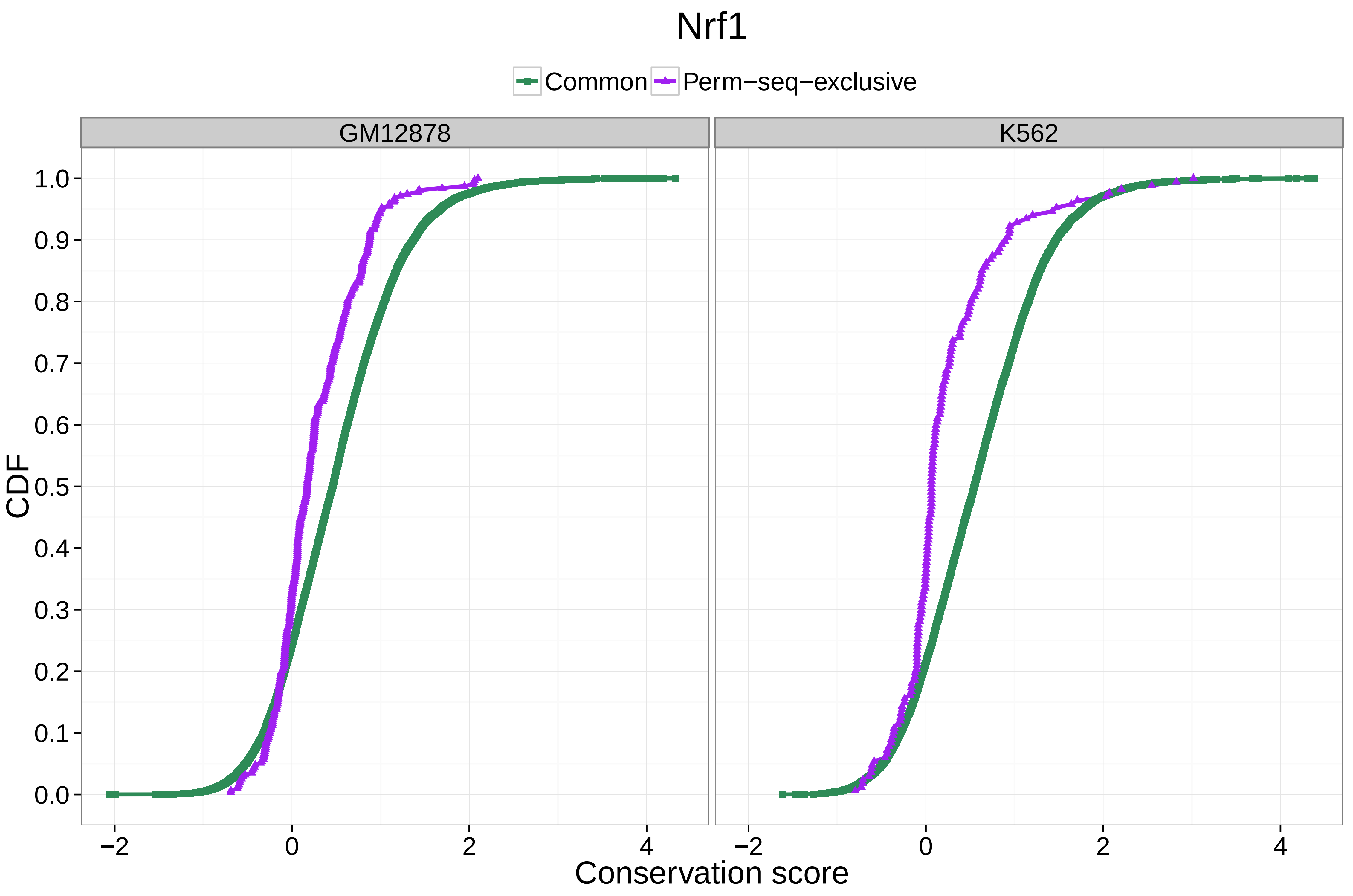
Nrf1
Common
Perm−seq−exclusive
GM12878
K562
CDF
1.0
0.9
0.8
0.7
0.6
0.5
0.4
0.3
0.2
0.1
0.0
−2
0
2
4
Conservation score

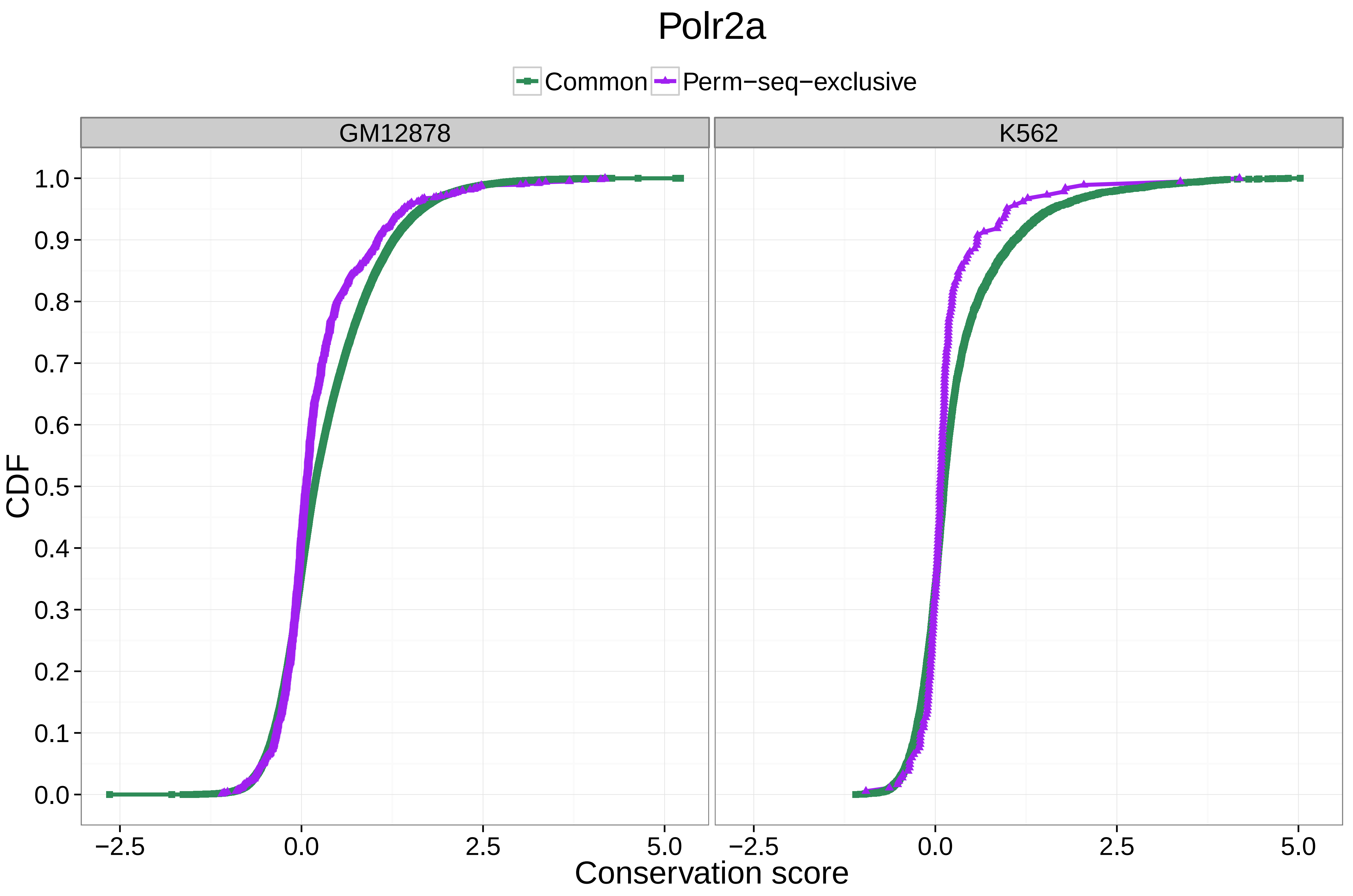

Polr2a
Common
Perm−seq−exclusive
GM12878
K562
CDF
1.0
0.9
0.8
0.7
0.6
0.5
0.4
0.3
0.2
0.1
0.0
−2.5
0.0
2.5
5.0
Conservation score

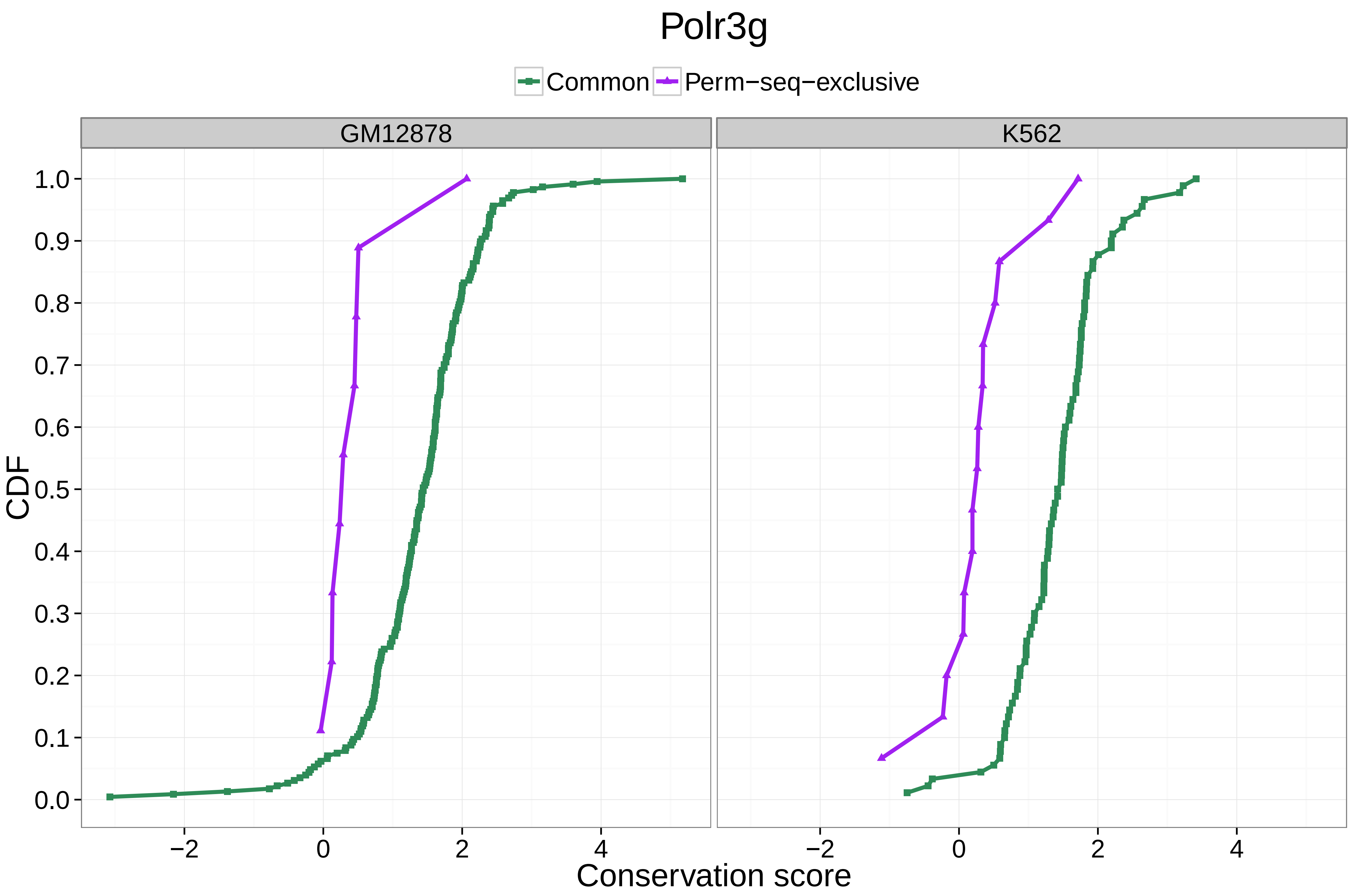
Polr3g
Common
Perm−seq−exclusive
GM12878
K562
CDF
1.0
0.9
0.8
0.7
0.6
0.5
0.4
0.3
0.2
0.1
0.0
−2
0
2
4
Conservation score

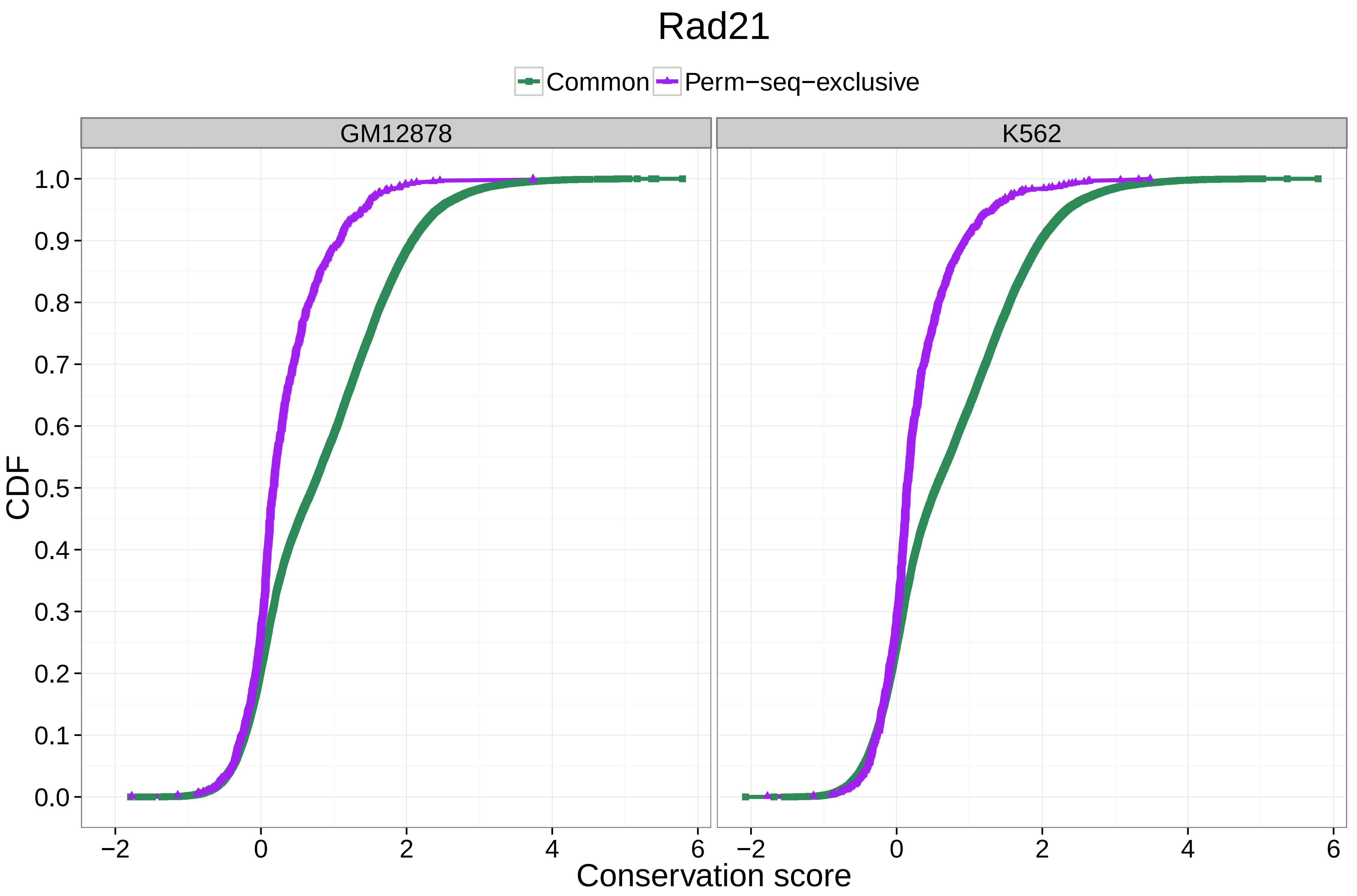

Rad21
Common
Perm−seq−exclusive
GM12878
K562
CDF
1.0
0.9
0.8
0.7
0.6
0.5
0.4
0.3
0.2
0.1
0.0
−2
0
2
4
6
Conservation score
