# Supplementary material for: Perm-seq: Mapping Protein-DNA Interactions in Segmental Duplication and Highly Repetitive Regions of Genomes with Prior-Enhanced Read Mapping
Source: PLoS Comput Biol. 2015 Oct 20;11(10):e1004491. doi: 10.1371/journal.pcbi.1004491 (PMC4618727; doi:10.1371/journal.pcbi.1004491)
Supplement: S5 Text — (PDF) [file pcbi.1004491.s005.pdf]

Supplementary File 5 for "Mapping protein-DNA interactions in segmental duplication and highly repetitive regions of genomes with prior-enhanced read mapping"

Xin Zeng[1], Bo Li[3], Rene Welch[1], Colin Dewey[2] & Sündüz Keleş[1,2]
[1] Department of Statistics, University of Wisconsin, Madison, WI, U.S.A.
[2] Department of Biostatistics and Medical Informatics, University of Wisconsin, Madison, WI, U.S.A.
[3] California Institute for Quantitative Biosciences, University of California, Berkeley, CA, U.S.A.

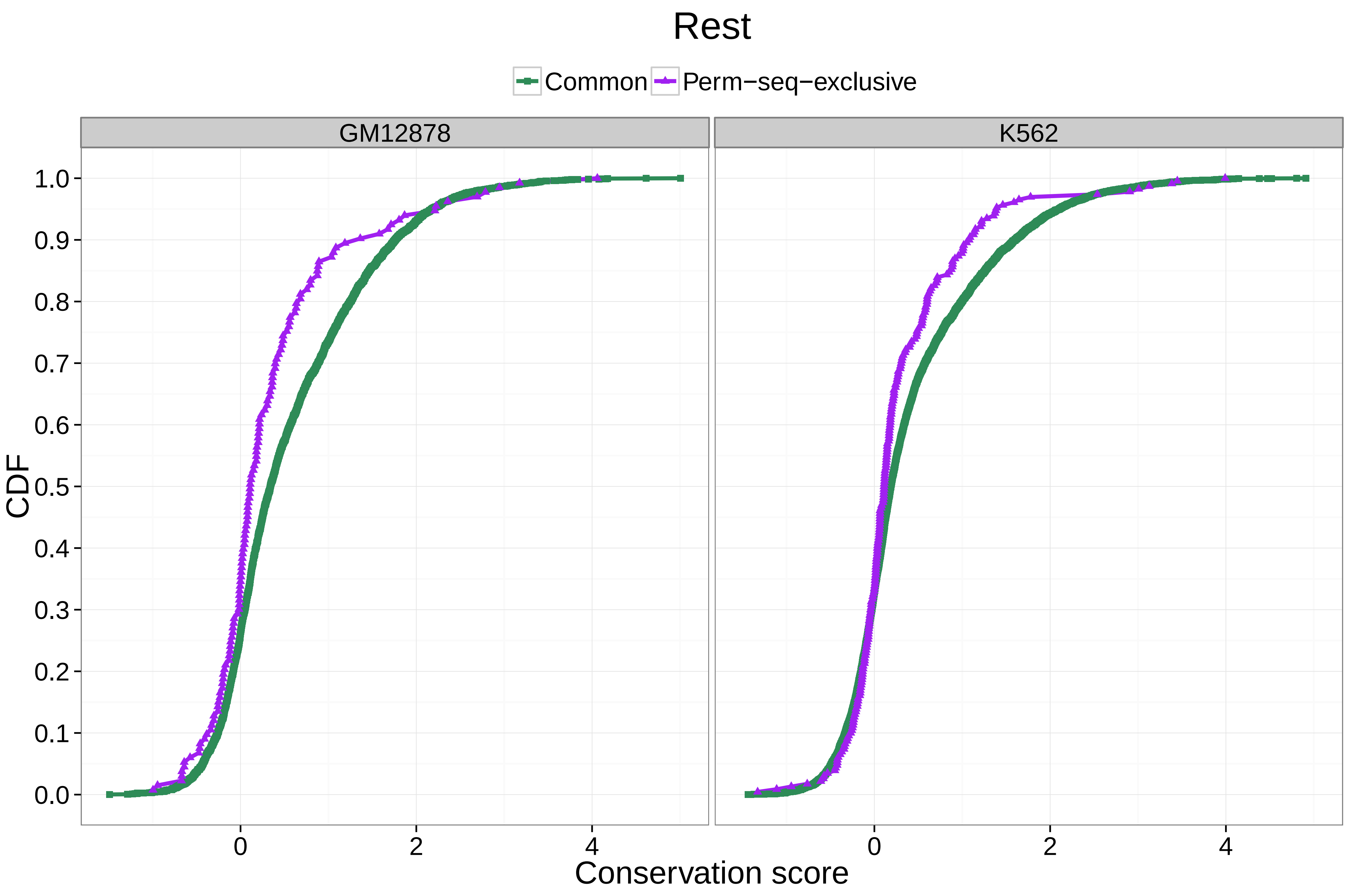

Rest
Common
Perm−seq−exclusive
GM12878
K562
CDF
1.0
0.9
0.8
0.7
0.6
0.5
0.4
0.3
0.2
0.1
0.0
0
2
4
Conservation score

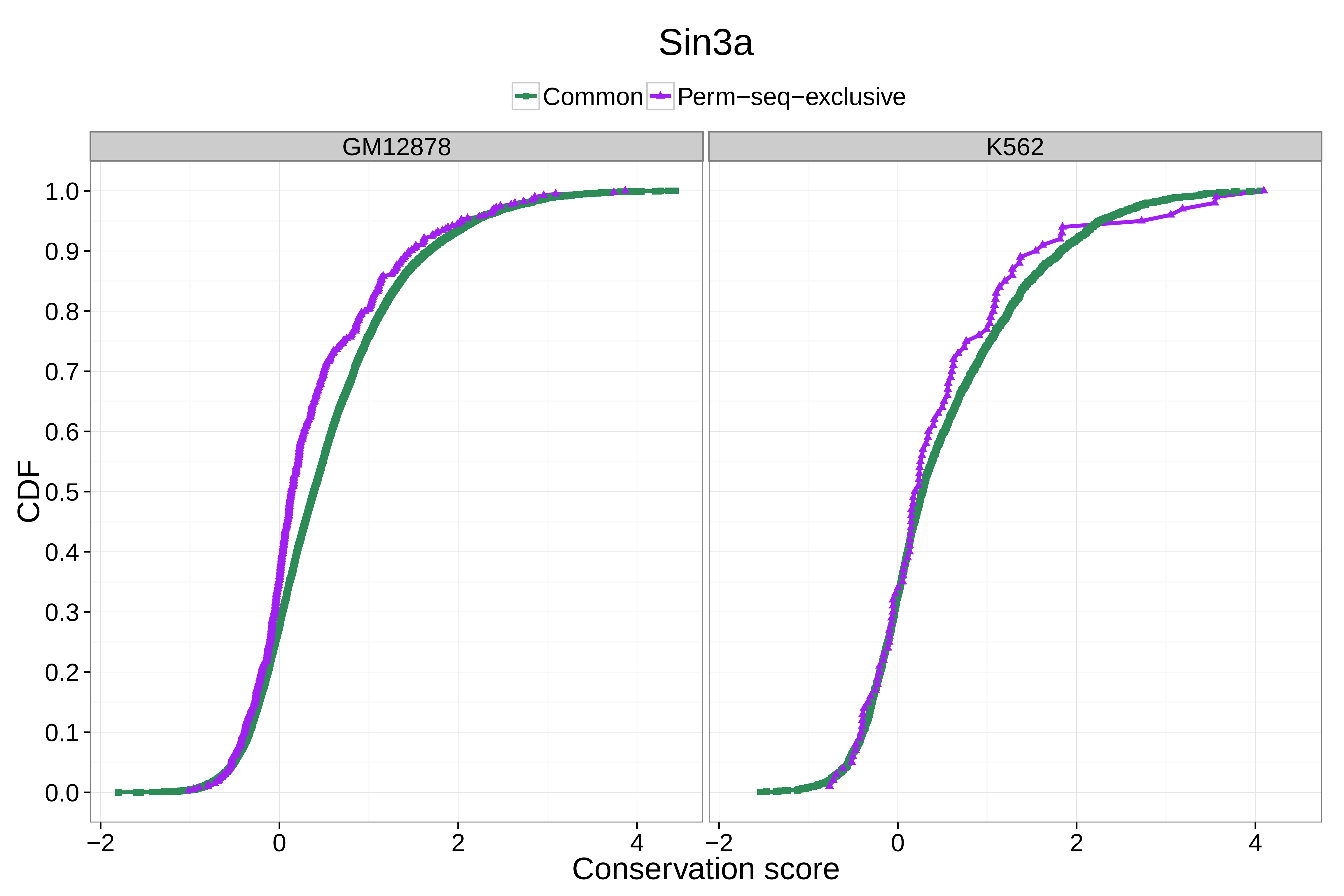
Sin3a
Common
Perm−seq−exclusive
GM12878
K562
CDF
Conservation score
1.0
0.9
0.8
0.7
0.6
0.5
0.4
0.3
0.2
0.1
0.0
−2
0
2
4

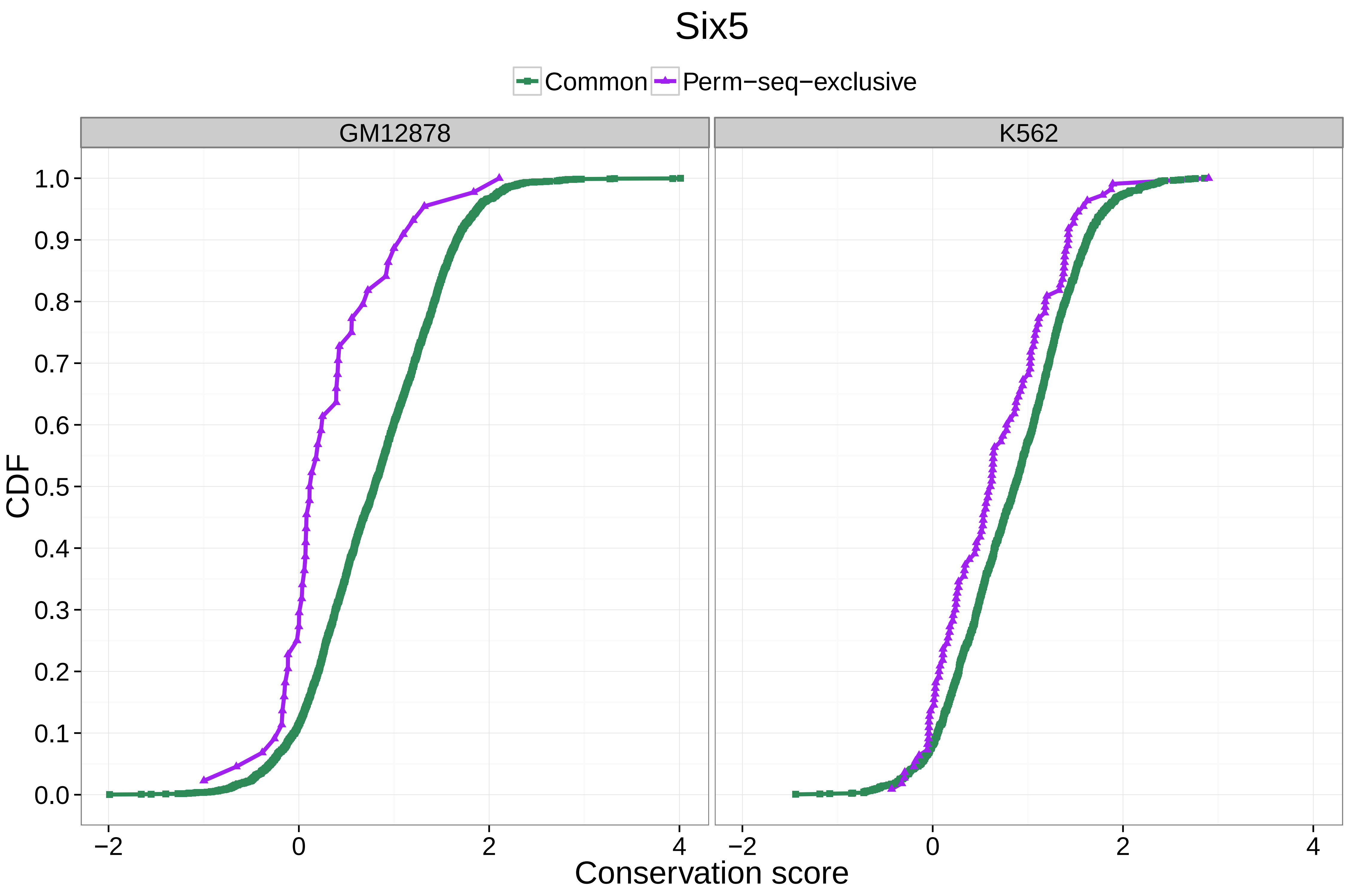

Six5
Common
Perm−seq−exclusive
GM12878
K562
CDF
1.0
0.9
0.8
0.7
0.6
0.5
0.4
0.3
0.2
0.1
0.0
−2
0
2
4
Conservation score

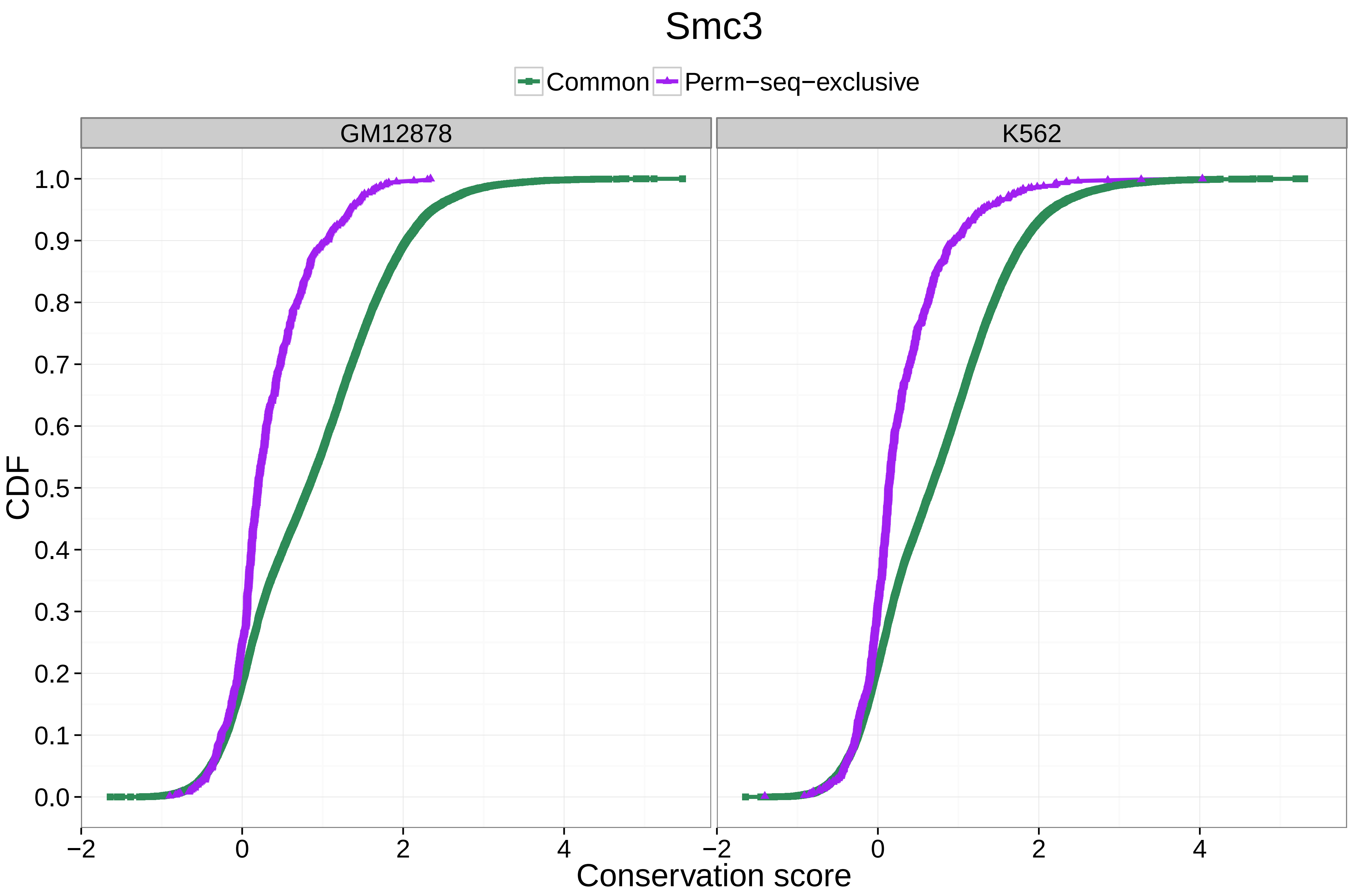

Smc3
Common
Perm−seq−exclusive
GM12878
K562
CDF
1.0
0.9
0.8
0.7
0.6
0.5
0.4
0.3
0.2
0.1
0.0
−2
0
2
4
−2
0
2
4
Conservation score

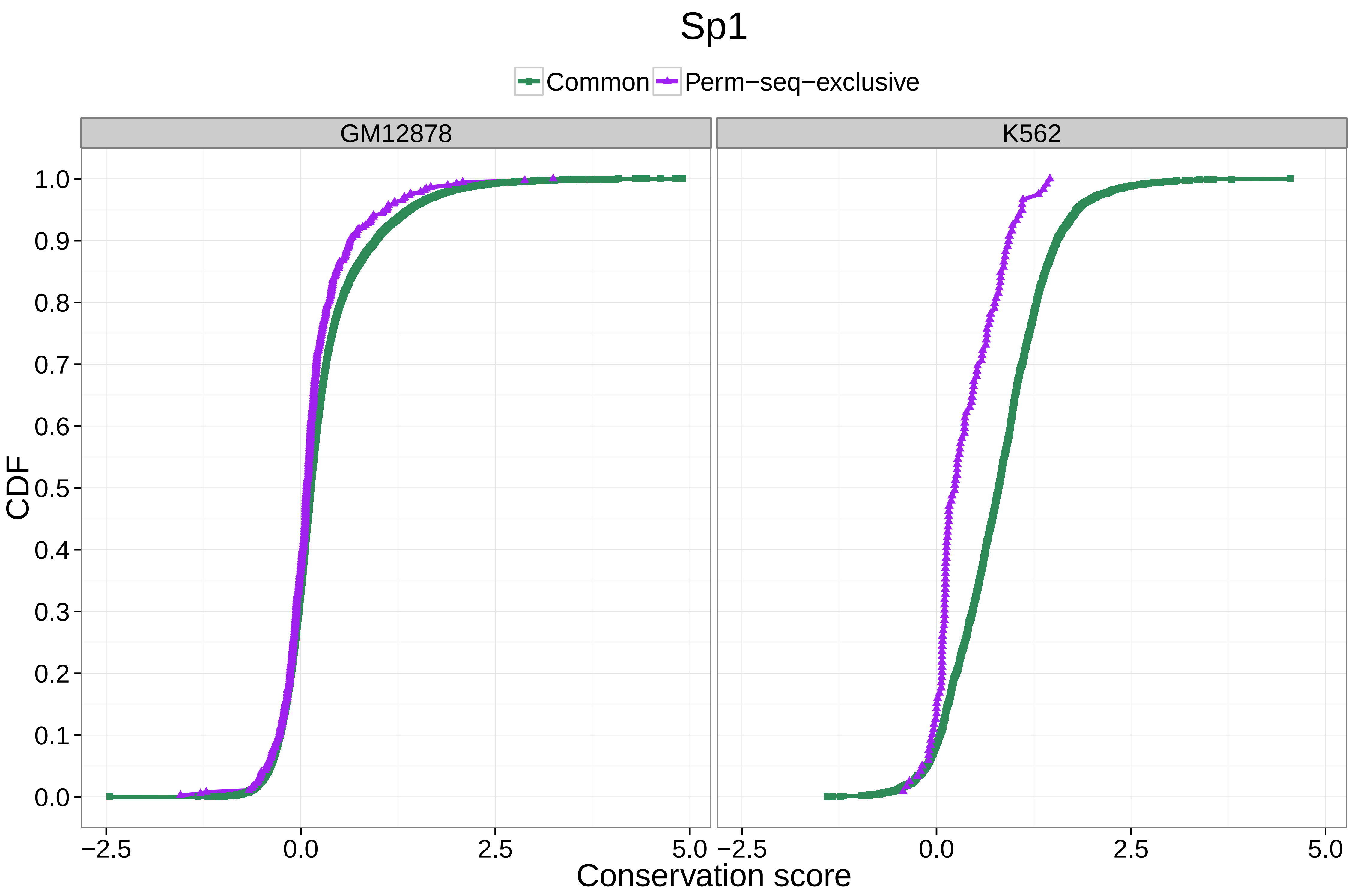
Sp1
Common
Perm−seq−exclusive
GM12878
K562
CDF
1.0
0.9
0.8
0.7
0.6
0.5
0.4
0.3
0.2
0.1
0.0
−2.5
0.0
2.5
5.0
Conservation score

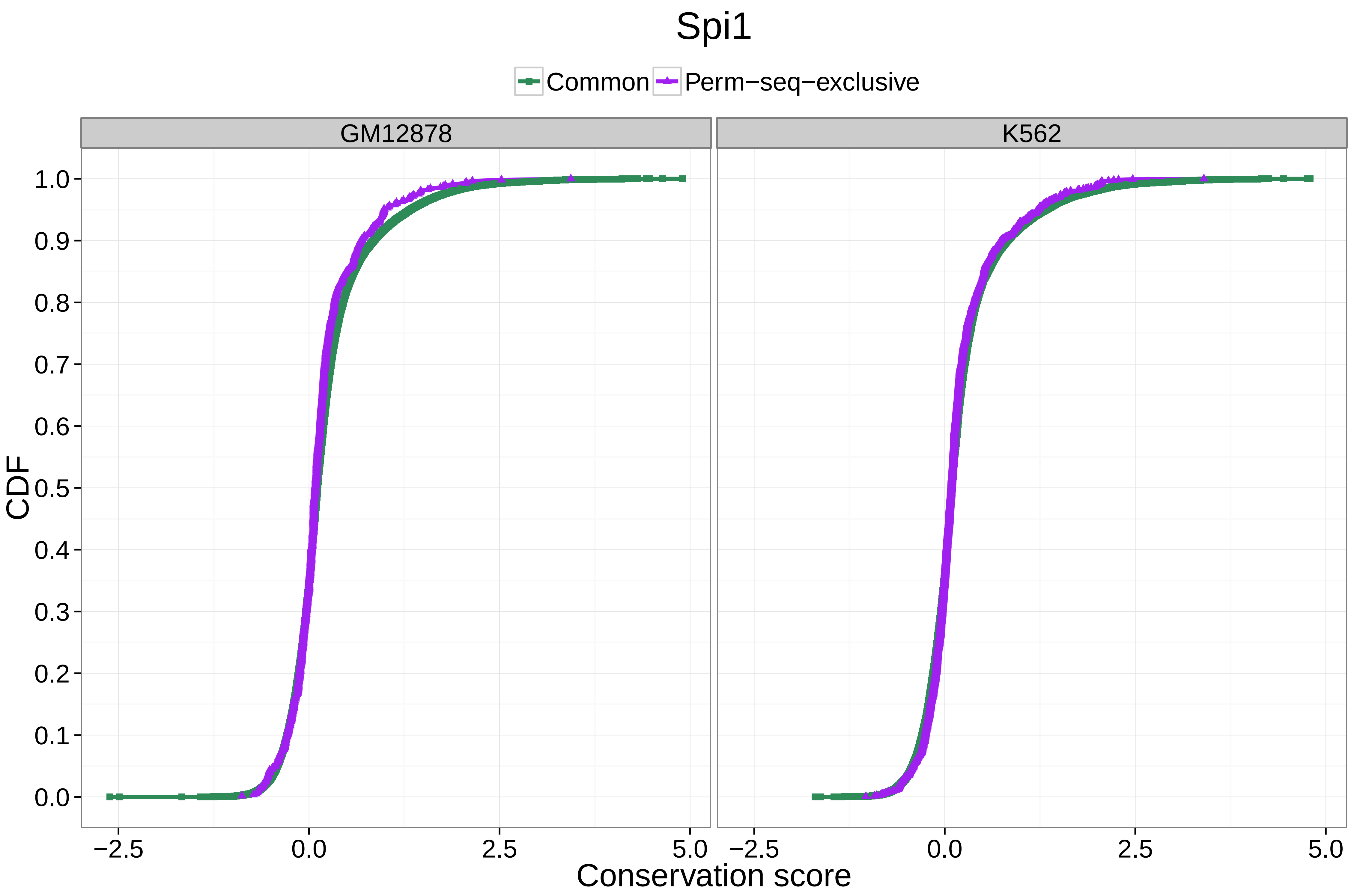

Spi1
Common
Perm−seq−exclusive
GM12878
K562
CDF
1.0
0.9
0.8
0.7
0.6
0.5
0.4
0.3
0.2
0.1
0.0
−2.5
0.0
2.5
5.0
Conservation score

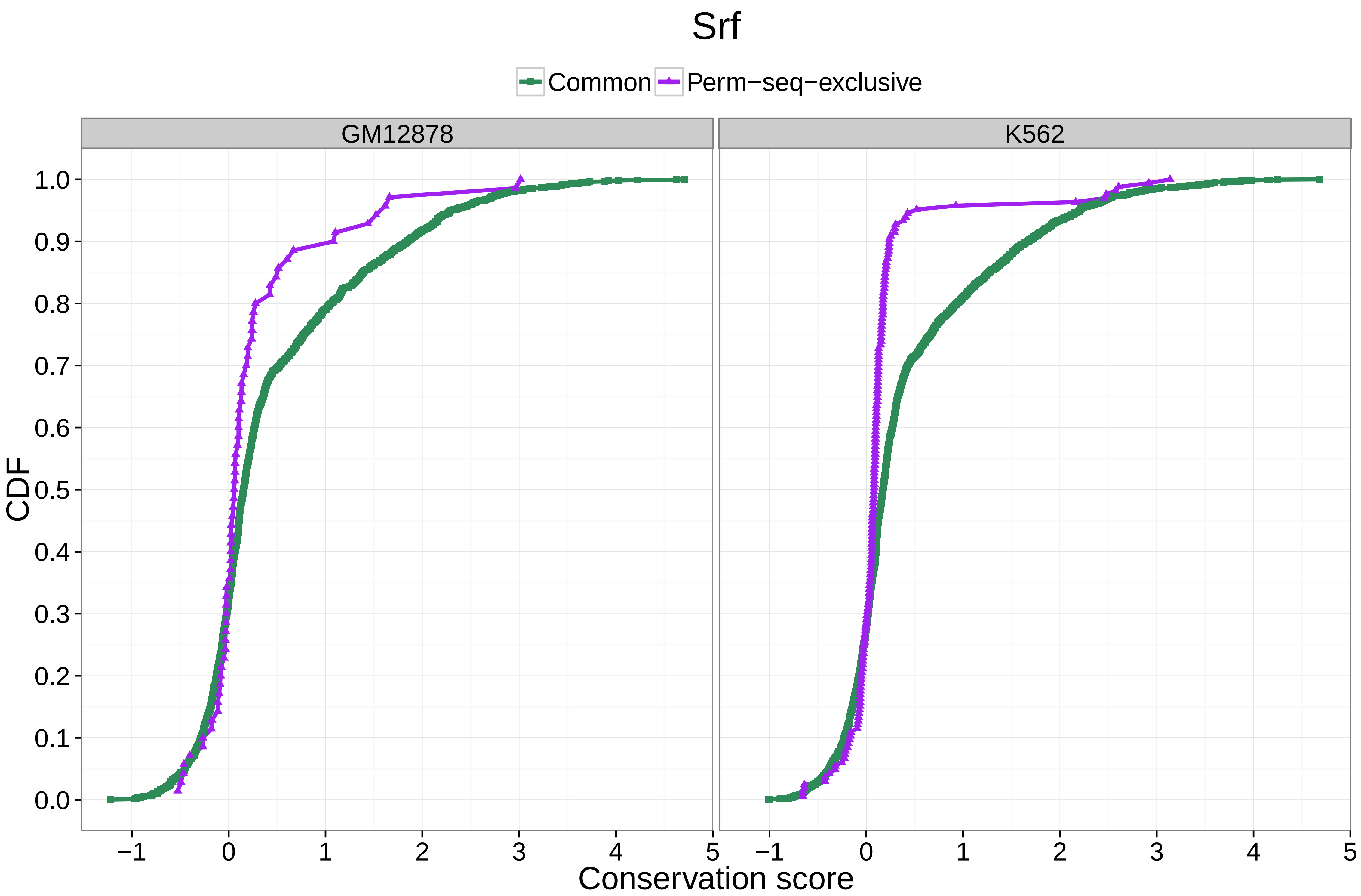

Srf
Common
Perm−seq−exclusive
GM12878
K562
CDF
1.0
0.9
0.8
0.7
0.6
0.5
0.4
0.3
0.2
0.1
0.0
−1
0
1
2
3
4
5
Conservation score

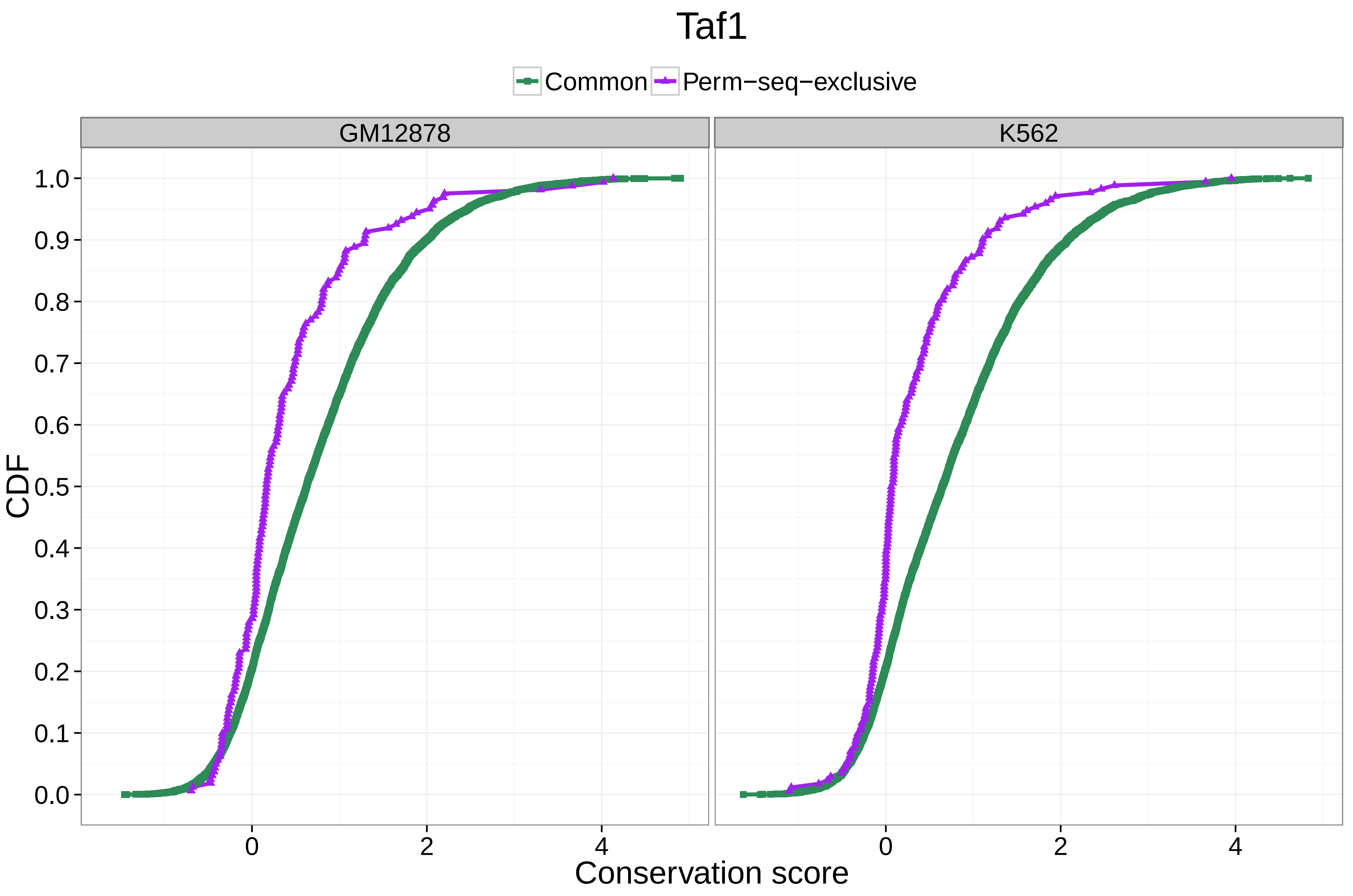

Taf1
Common
Perm−seq−exclusive
GM12878
K562
CDF
1.0
0.9
0.8
0.7
0.6
0.5
0.4
0.3
0.2
0.1
0.0
0
2
4
Conservation score

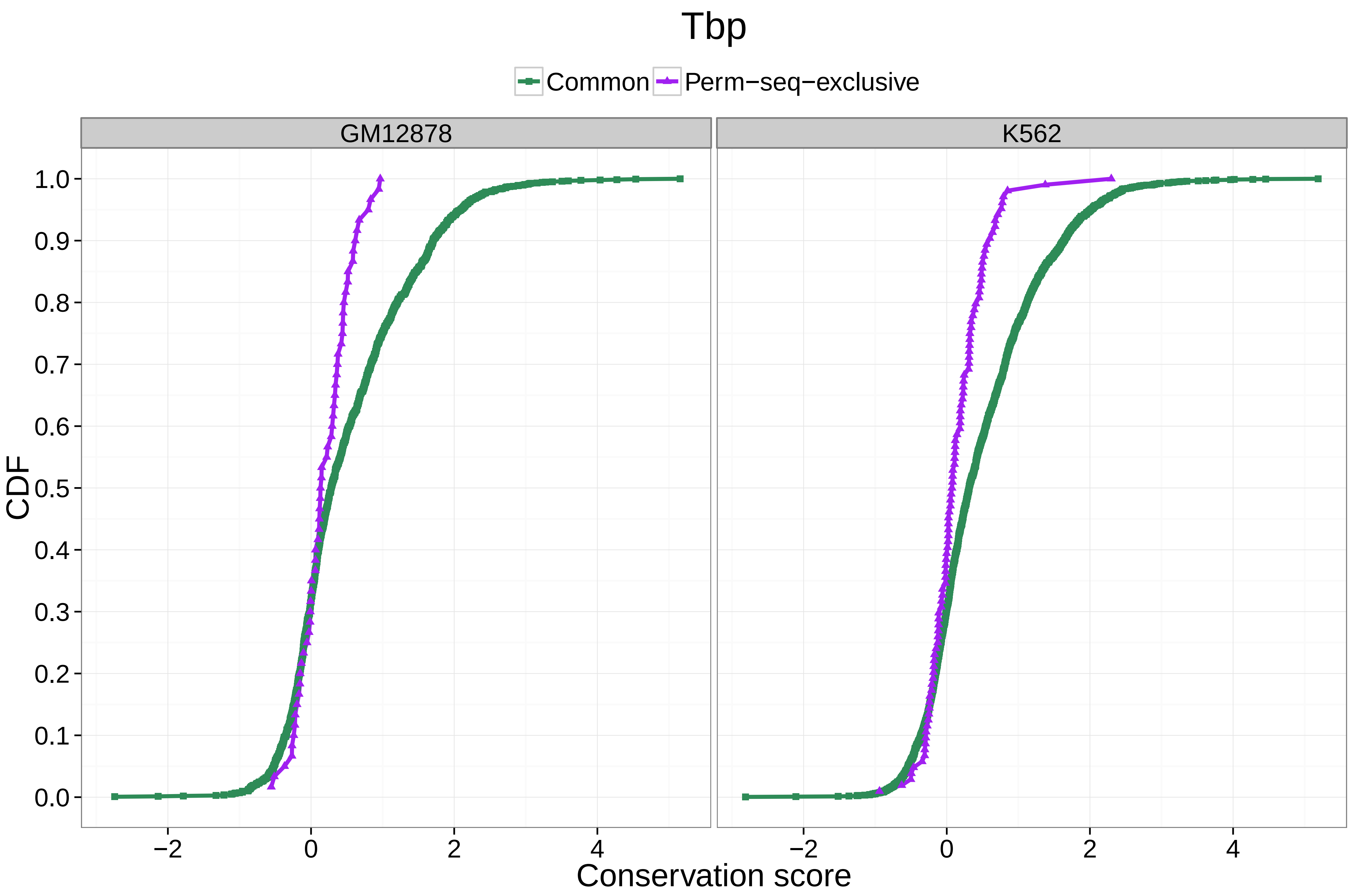

Tbp
Common
Perm−seq−exclusive
GM12878
K562
CDF
1.0
0.9
0.8
0.7
0.6
0.5
0.4
0.3
0.2
0.1
0.0
−2
0
2
4
Conservation score

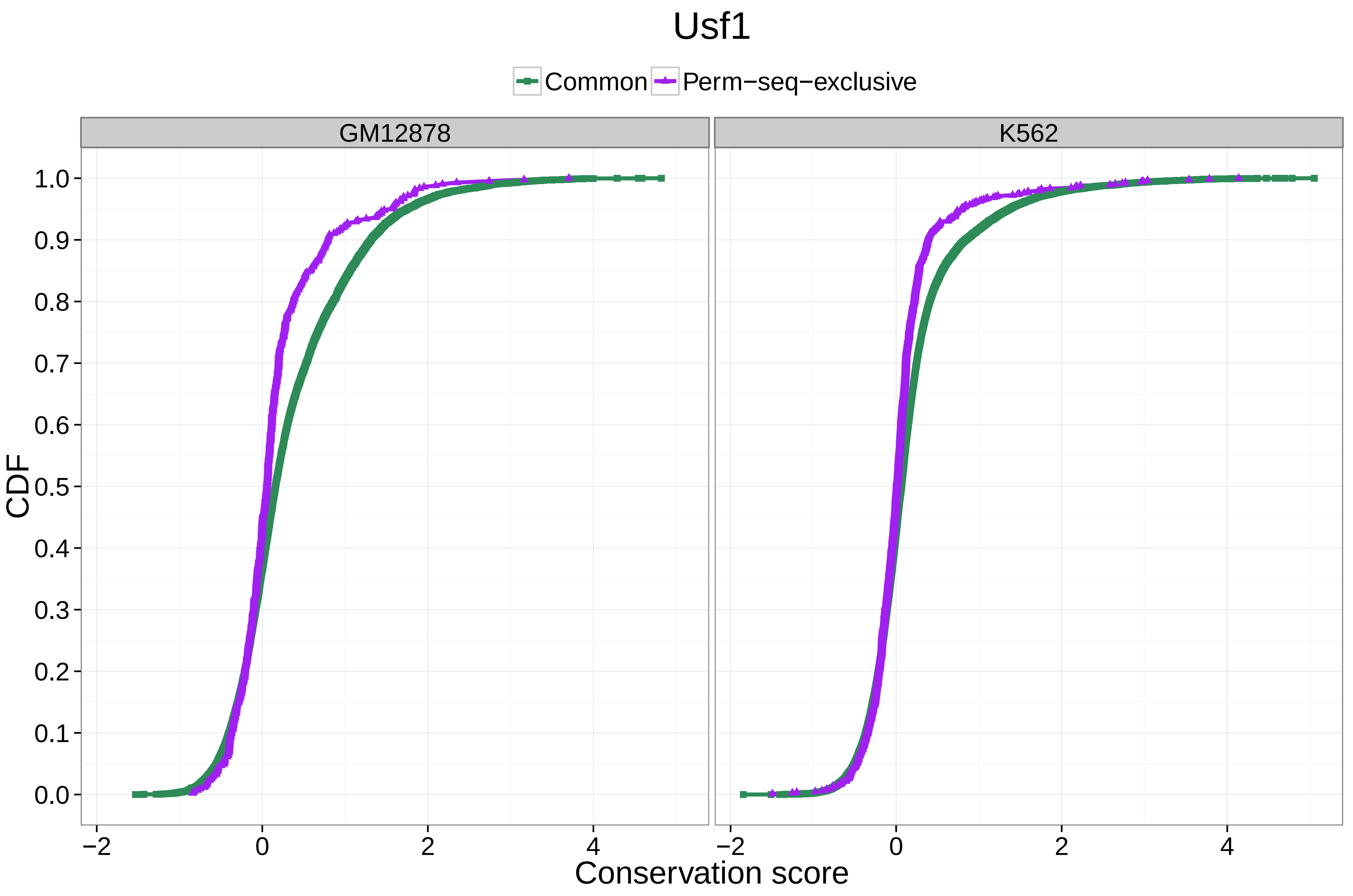
Usf1
Common
Perm−seq−exclusive
GM12878
K562
CDF
1.0
0.9
0.8
0.7
0.6
0.5
0.4
0.3
0.2
0.1
0.0
−2
0
2
4
Conservation score

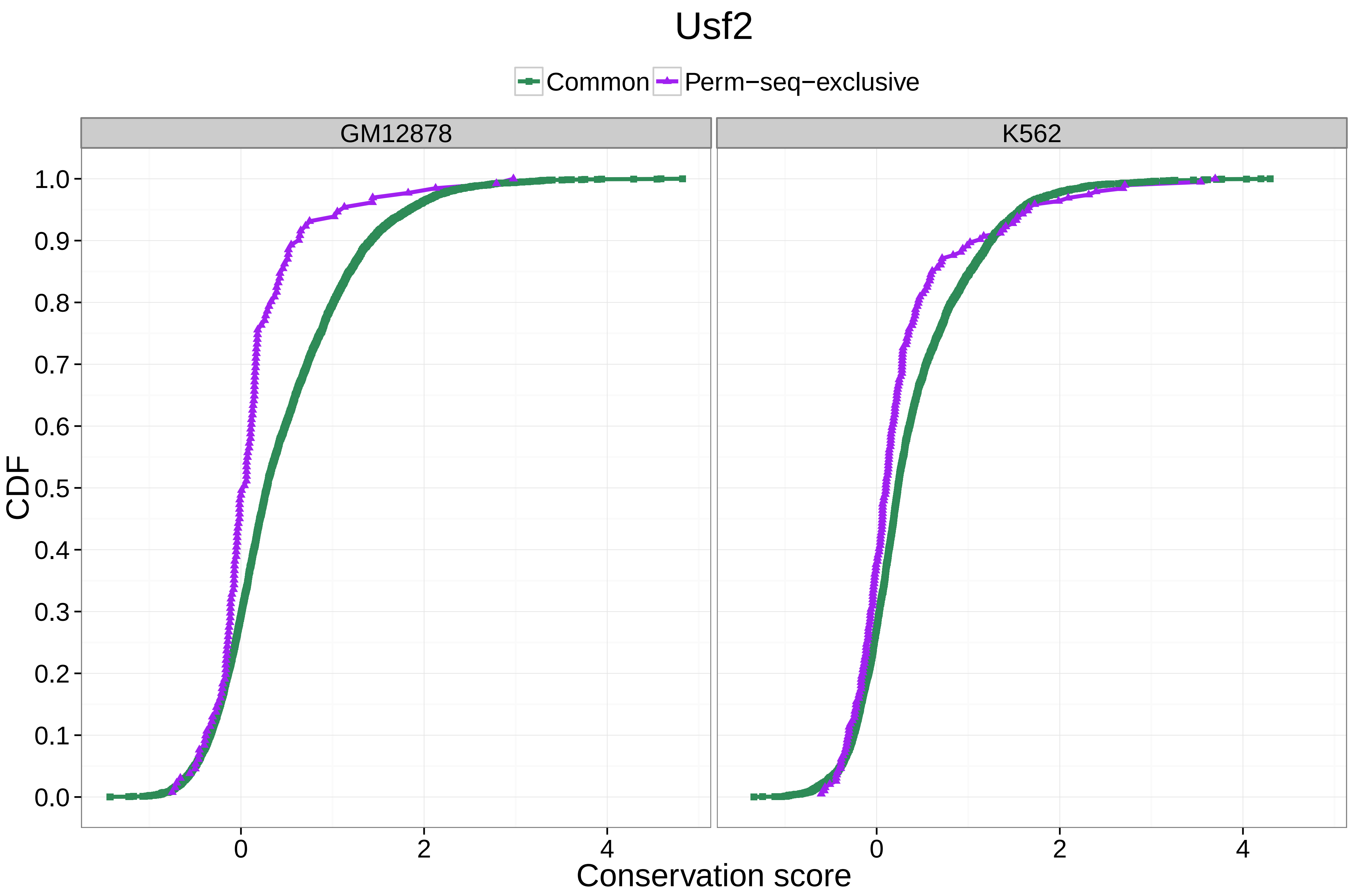

Usf2
Common
Perm−seq−exclusive
GM12878
K562
CDF
1.0
0.9
0.8
0.7
0.6
0.5
0.4
0.3
0.2
0.1
0.0
0
2
4
Conservation score

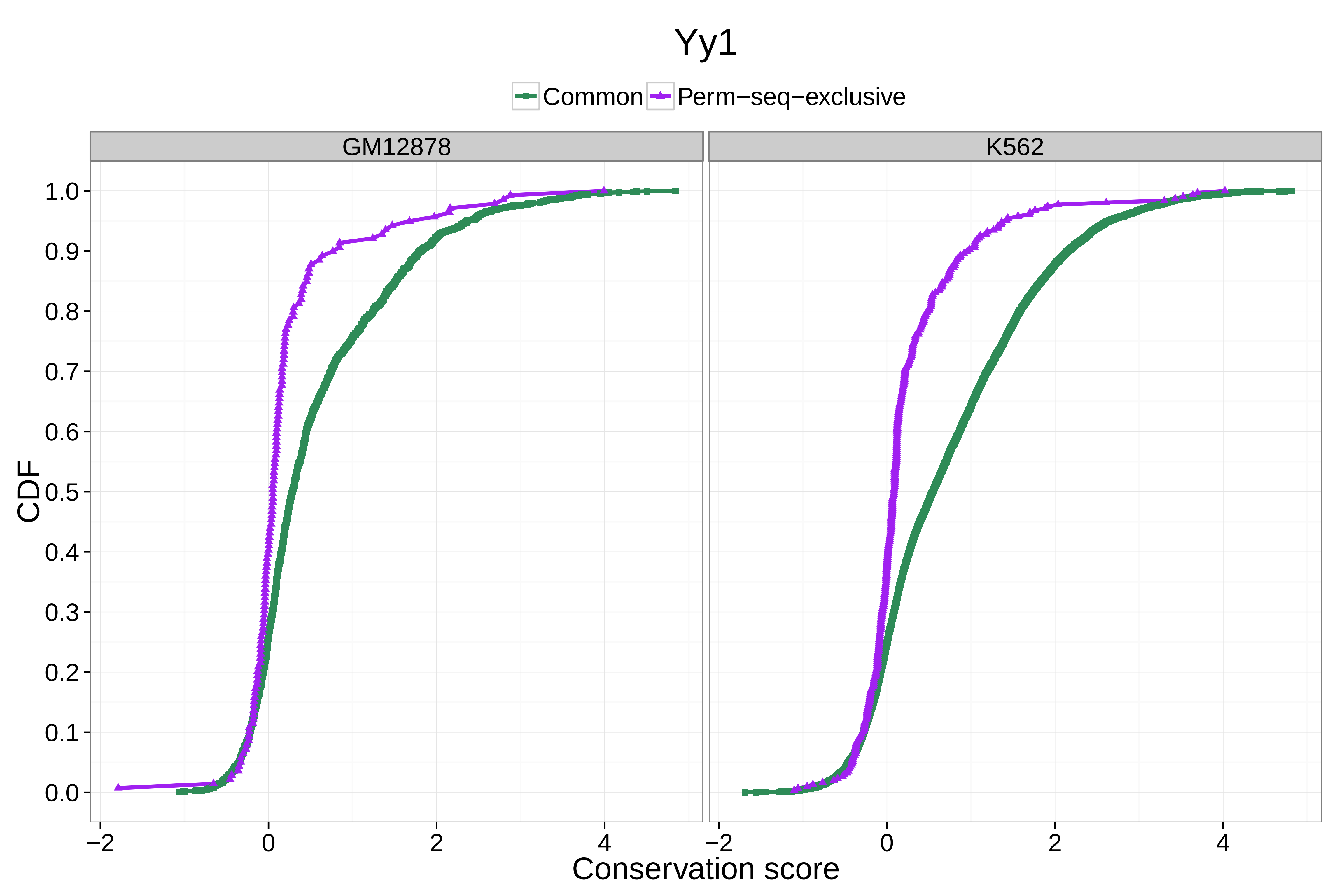

Yy1
Common
Perm−seq−exclusive
GM12878
K562
CDF
1.0
0.9
0.8
0.7
0.6
0.5
0.4
0.3
0.2
0.1
0.0
−2
0
2
4
Conservation score

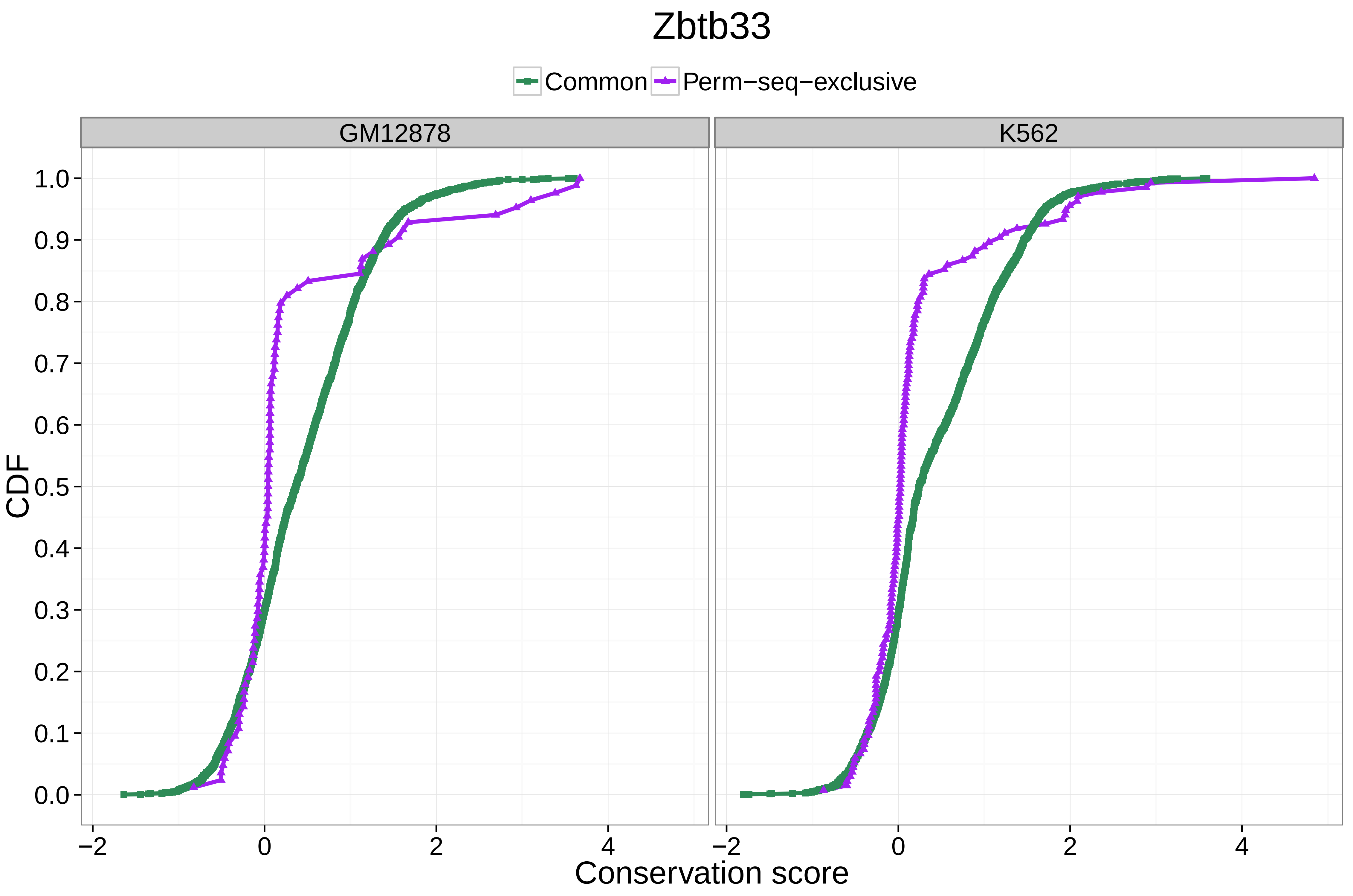
Zbtb33
Common
Perm−seq−exclusive
GM12878
K562
CDF
1.0
0.9
0.8
0.7
0.6
0.5
0.4
0.3
0.2
0.1
0.0
−2
0
2
4
Conservation score
